# A pyridinic Fe-N_4_ macrocycle models the active sites in Fe/N-doped carbon electrocatalysts

**DOI:** 10.1038/s41467-020-18969-6

**Published:** 2020-10-19

**Authors:** Travis Marshall-Roth, Nicole J. Libretto, Alexandra T. Wrobel, Kevin J. Anderton, Michael L. Pegis, Nathan D. Ricke, Troy Van Voorhis, Jeffrey T. Miller, Yogesh Surendranath

**Affiliations:** 1 Department of Chemistry, Massachusetts Institute of Technology, Cambridge, MA 02139 USA; 2 Davidson School of Chemical Engineering, Purdue University, West Lafayette, IN 47097 USA; 3 Chemical Science and Engineering Division, Argonne National Laboratory, Argonne, Illinois, 60439 USA; 4 Department of Chemistry and Chemical Biology, Harvard University, Cambridge, MA 02138 USA

**Keywords:** Electrocatalysis, Electrocatalysis

## Abstract

Iron- and nitrogen-doped carbon (Fe-N-C) materials are leading candidates to replace platinum catalysts for the oxygen reduction reaction (ORR) in fuel cells; however, their active site structures remain poorly understood. A leading postulate is that the iron-containing active sites exist primarily in a pyridinic Fe-N_4_ ligation environment, yet, molecular model catalysts generally feature pyrrolic coordination. Herein, we report a molecular pyridinic hexaazacyclophane macrocycle, (phen_2_N_2_)Fe, and compare its spectroscopic, electrochemical, and catalytic properties for ORR to a typical Fe-N-C material and prototypical pyrrolic iron macrocycles. N 1s XPS and XAS signatures for (phen_2_N_2_)Fe are remarkably similar to those of Fe-N-C. Electrochemical studies reveal that (phen_2_N_2_)Fe has a relatively high Fe(III/II) potential with a correlated ORR onset potential within 150 mV of Fe-N-C. Unlike the pyrrolic macrocycles, (phen_2_N_2_)Fe displays excellent selectivity for four-electron ORR, comparable to Fe-N-C materials. The aggregate spectroscopic and electrochemical data demonstrate that (phen_2_N_2_)Fe is a more effective model of Fe-N-C active sites relative to the pyrrolic iron macrocycles, thereby establishing a new molecular platform that can aid understanding of this important class of catalytic materials.

## Abstract (web summary)

Iron- and nitrogen-doped carbon materials are effective catalysts for the oxygen reduction reaction whose active sites are poorly understood. Here, the authors establish a new pyridinic iron macrocycle complex as a more effective active site model relative to legacy pyrrolic model complexes.

## Introduction

The four-electron, four-proton reduction of molecular oxygen to water is the efficiency-limiting half reaction in low-temperature fuel cells^1^. Regardless of the fuel source used, the current density output is primarily limited by the slow electron transfer kinetics of the oxygen reduction reaction (ORR) taking place at the cathode^2^. The prototypical material for catalyzing this reaction in commercial fuel cells is platinum metal (Pt) supported on carbon. However, the high cost and scarcity of Pt impedes the large-scale deployment of fuel cell devices and motivates the development of Earth-abundant electrocatalysts for oxygen reduction. These catalysts must operate at low overpotentials^3–5^ and with high selectivity for the four-electron reduction of oxygen to water instead of the two-electron reduction process to generate hydrogen peroxide^6–9^. Since early reports of oxygen reduction catalyzed by macrocyclic first-row transition metal complexes^10,11^, there has been a global effort to develop selective and efficient ORR catalysts featuring base metal active sites^12^.

Pyrolyzed iron- and nitrogen-doped (Fe-N-C) materials are leading Earth-abundant alternatives to Pt-based ORR electrocatalysts^13^, however marked increases in catalyst performance are needed to make these materials technologically viable. Recent years have witnessed significant improvements in their performance^14^, however systematic development of these materials is hampered by limited molecular-level understanding or control of the iron active sites^15^. Fe-N-C materials are typically prepared by the high-temperature pyrolysis of finely dispersed iron salts^16^, porphyrins^17^, or phthalocyanines^18^ along with a metal-organic framework (MOF)^19^ or carbon-based support^20^. The uncontrolled nature of pyrolysis leads to a wide diversity of iron environments as well as extended solid iron phases in the resulting Fe-N-C materials^21,22^. This poor control, combined with the wide variability in preparative procedures, has led to longstanding uncertainty about the local structure of the iron active sites responsible for ORR, thereby impeding systematic enhancements to catalytic performance^23^.

**Fig. 1:**
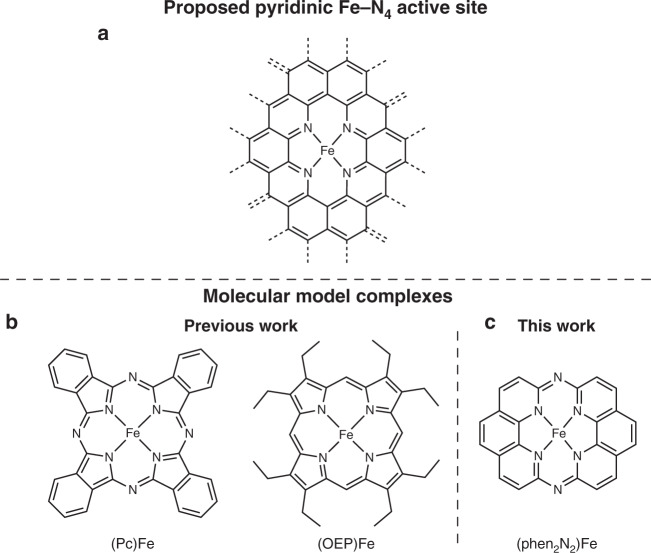
Putative Fe-N_4_ ORR active site and molecular model structures. Proposed Fe-N_4_ active site in Fe-N-C materials (**a**). Molecular structures of (Pc)Fe and (OEP)Fe (**b**) and (phen_2_N_2_)Fe (**c**) model compounds.

Numerous recent studies have provided significant insight into possible Fe-N-C active site structures. While metallic iron active sites have been postulated^24,25^, mononuclear Fe-N_4_ active sites are more commonly invoked to explain correlations between X-ray absorption spectroscopy (XAS) or ^57^Fe Mössbauer spectroscopy with ORR activity^16^. Despite the growing consensus that Fe-N_4_ sites are essential for ORR, the ligation environment of these iron sites remains uncertain^26,27^. Indeed, even though iron porphyrin and phthalocyanine complexes can be used as precursors to Fe-N-C materials, there is evidence that the core pyrrolic ligation environment of these precursors changes substantially upon pyrolysis^28–31^. In particular, the first shell Fe–N bond lengths in Fe-N-C materials have been reported to be shorter than the macrocyclic iron complex precursor used in the material synthesis^17^. Furthermore, X-ray photoelectron spectroscopy (XPS) results have pointed to the presence of metal-coordinated pyridinic nitrogen moieties as opposed to metal-bound pyrrolic nitrogens in Fe-N-C materials^32^. ^57^Fe Mössbauer spectra of many iron porphyrin^33^ and phthalocyanine^34^ complexes differ dramatically from the main ^57^Fe Mössbauer doublets assigned to the putative Fe-N_4_ active sites in Fe-N-C materials^35–37^. In addition, atomic-resolution electron microscopy data indicate the presence of mono-dispersed iron atoms bound within the plane of graphitic carbon^38–42^ suggesting that the ligating groups are six-membered heterocycles rather than the five-membered rings found in pyrrolic macrocycles. Based on these spectroscopic and imaging results, there is a growing body of evidence that the Fe-N_4_ sites in Fe-N-C materials are ligated by pyridinic moieties fused within graphitic sheets (Fig. 1a).

Despite substantial evidence for pyridinic Fe-N_4_ sites in Fe-N-C materials, nearly all molecular Fe-based ORR catalysts that are used to model the iron-containing active sites of Fe-N-C are macrocyclic complexes that feature pyrrolic coordination environments (Fig. 1b). Notwithstanding evident differences between the spectroscopic features of Fe-N-C and pyrrolic-type macrocycles, such macrocyclic complexes are routinely used as models during the analysis of X-ray absorption data to build computational fits to the experimental Fe-N-C data in order to construct toy models of the apparent Fe-N_4_ sites. Such an approach could be strengthened by the creation of a pyridinic Fe-N_4_ model architecture that is better able to model the average Fe-N_4_ sites in Fe-N-C relative to pyrrolic iron complexes. Owing to the inherent multi-scale complexity of Fe-N-C materials, no molecular complex will ever be able to perfectly reproduce their spectroscopic signatures or catalytic function. Pyrrolic model compounds have offered valuable spectroscopic and mechanistic insights and such molecular-level studies would benefit from new model complexes that feature the pyridinic ligation motifs that are now thought to exist in Fe-N-C materials.

Based on the aggregate spectroscopic evidence on Fe-N-C materials, we envisioned that an improved model complex would feature: (a) a tetrapyridinic coordination environment, (b) relatively short Fe–N bond lengths^17^, (c) an extended ligand π system^29,30^ capable of stabilizing the Fe(II) state^43,44^, and (d) a relatively high Fe(III)-OH/Fe(II)-OH_2_ redox potential. Towards realizing the above goal of synthesizing a pyridinic Fe-N_4_ model complex, we searched the literature for pyridinic N_4_ macrocyclic ligands. Notably, the aza-bridged bis-1,10-phenanthroline hexaazamacrocycle, (phen_2_N_2_)H_2_ (phen_2_N_2_ = 1,14:7,8-diethenotetrapyrido[2,1,6-*de*:2′,1′,6′-*gh*:2″,1″,6″-*kl*:2″′,l″′,6″′-*na*][1,3,5,8,10,12]hexaazacyclotetradecine), as well as its cobalt(II), nickel(II) and copper(II) complexes have been reported^45^ and applied to DNA binding studies^46^ and carbon dioxide reduction catalysis^47^. Despite this precedent, the synthesis and ORR activity of the corresponding (phen_2_N_2_)Fe fragment^44^ has, to the best of our knowledge, remained unexplored.

Herein, we report the synthesis and characterization of this pyridinic Fe-N_4_ macrocyclic fragment (Fig. 1c) and compare its ^57^Fe Mössbauer, XPS, and XAS features and ORR performance to those of prototypical Fe-N-C, iron octaethylporphyrin, (OEP)Fe, and iron phthalocyanine, (Pc)Fe, catalysts. Through these studies, we demonstrate that the iron coordination environment in [(phen_2_N_2_)Fe]_2_O displays greater spectroscopic similarity to Fe-N-C materials than [(OEP)Fe]_2_O and [(Pc)Fe]_2_O. Furthermore, we find that (phen_2_N_2_)FeCl displays superior catalytic activity and selectivity relative to (OEP)FeCl and (Pc)FeCl, approaching the performance metrics of Fe-N-C materials. These studies establish (phen_2_N_2_)Fe complexes as better structural and functional mimics of Fe-N_4_ active sites in Fe-N-C materials than legacy pyrrolic macrocycles.

## Results and discussion

### Synthesis of Fe-N-C and (phen_2_N_2_)Fe complexes

A prototypical Fe-N-C material was synthesized by a combination of literature methods^19,48^. In brief, Fe-N-C was prepared by pyrolysis of a mixture of iron(II) acetate, 1,10-phenanthroline, and a zinc imidazolate MOF (ZIF-8) under a reducing atmosphere (5% H_2_ in Ar). After pyrolysis, the sample was washed with 0.1 M H_2_SO_4_ to remove trace Fe(0). The ^57^Fe Mössbauer spectrum of this material (Supplementary Fig. 1, top and Supplementary Table 1) displays two quadrupole doublets in line with previously reported Fe-N-C materials.

**Fig. 2:**
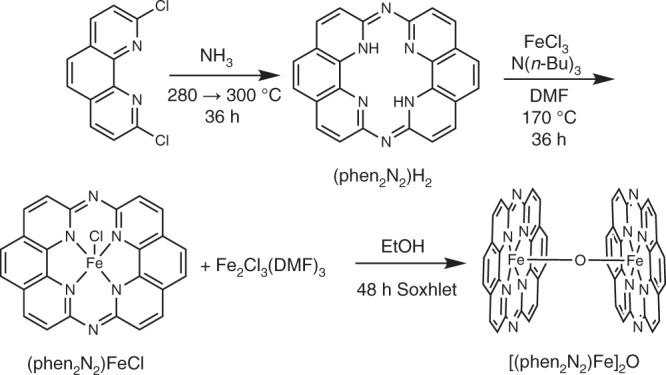
Syntheses of (phen_2_N_2_)H_2_, (phen_2_N_2_)FeCl, and [(phen_2_N_2_)Fe]_2_O. The (phen_2_N_2_)H_2_ macrocycle was synthesized by the reaction of 2,9-dichloro-1,10-phenanthroline with anhydrous ammonia. The (phen_2_N_2_)FeCl model complex was obtained by treatment of (phen_2_N_2_)H_2_ with FeCl_3_ in the presence of N(*n*-Bu)_3_ under an inert atmosphere. Continuously washing (phen_2_N_2_)FeCl with hot ethanol for 48 hours using a Soxhlet apparatus yielded the [(phen_2_N_2_)Fe]_2_O model complex.

The bis-phenanthroline macrocycle, (phen_2_N_2_)H_2_^49^, was prepared by exposure of 2,9-dichlorophenanthroline (Supplementary Fig. 2) to anhydrous ammonia in a Parr reactor that was held at 280 °C for 24 h and then ramped to 300 °C for 12 h (Fig. 2). Metalation with iron was achieved by treating (phen_2_N_2_)H_2_ with anhydrous FeCl_3_ in DMF or HMPA in the presence of excess tributylamine under an N_2_ atmosphere. To remove unreacted FeCl_3_ and other reaction byproducts, the crude reaction mixture was washed with copious amounts of diethyl ether and dichloromethane. Due to the extremely low solubility of the (phen_2_N_2_)FeCl complex, we found that residual Fe_2_Cl_6_•(DMF)_3_ (~16%) remained in the sample despite exhaustive washing with anhydrous solvents^50^. HR-MS analysis of the material reveals a prominent peak at 440.1061 *m/z* and a smaller peak at 475.1023 *m/z*, in line with the expected masses of [(phen_2_N_2_)Fe]^+^ (440.0473 *m/z*) and [(phen_2_N_2_)FeCl]^+^ (475.0161 *m/z*), respectively. UV–Vis data also support formation of the metalated complex with a bathochromic shift of the ligand π → π* transitions and the appearance of shoulders on the main π → π* peak (Supplementary Fig. 3). The ^57^Fe Mössbauer spectrum of (phen_2_N_2_)FeCl (Supplementary Fig. 1, middle and Supplementary Table 2) reveals a broad quadrupole doublet assigned to (phen_2_N_2_)FeCl in the *S* = 3/2 state^51–54^, which differs dramatically from the parameters for (OEP)FeCl and (Pc)FeCl (Supplementary Figs. 4 and 5 and Supplementary Table 2). This assignment is consistent with our calculations on (phen_2_N_2_)FeCl, which predict an intermediate-spin ground state and is consistent with a compressed N_4_-binding pocket that stabilizes the *S* = 3/2 spin state (see Supplementary Note 1 for calculated energies and geometries).

To better mimic the axial O-ligation present in the material, we Soxhleted the (phen_2_N_2_)FeCl sample with ethanol. This served to generate the μ-oxo [(phen_2_N_2_)Fe]_2_O via reaction with adventitious water and base. Similar conversions are precedented for sterically unprotected porphyrin complexes^55^. While this species is catalytically inactive in the acidic electrolyte (Supplementary Fig. 6)^56,57^, it provides a good spectroscopic model for Fe-N-C materials and the analogous [(OEP)Fe]_2_O and [(Pc)Fe]_2_O were synthesized for comparison^55,58,59^. The ^57^Fe Mössbauer spectrum of the [(phen_2_N_2_)Fe]_2_O complex is a narrow doublet (Supplementary Fig. 1, bottom and Supplementary Table 2), distinct from the spectra of [(OEP)Fe]_2_O and [(Pc)Fe]_2_O (Supplementary Figs. 7 and 8 and Supplementary Table 2). The vast differences in the ^57^Fe Mössbauer spectra of (phen_2_N_2_)Fe, (OEP)Fe, and (Pc)Fe upon exchange of the axial ligand suggest that ^57^Fe Mössbauer spectroscopy alone may be insufficient to distinguish pyrrolic from pyridinic ligation at putative Fe-N_4_ sites in Fe-N-C materials.

### XPS reveals nitrogen and iron environments similar to Fe-N-C

**Fig. 3:**
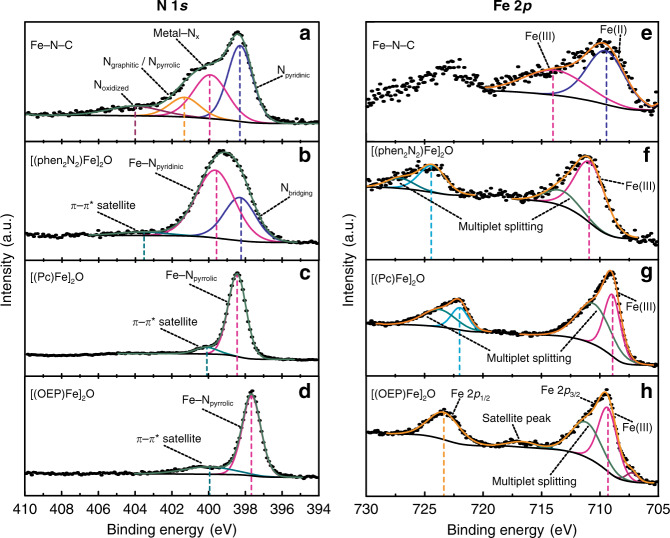
High-resolution N 1 s and Fe 2p XPS spectra. Fe-N-C (**a** and **e**), [(phen_2_N_2_)Fe]_2_O (**b** and **f**), [(Pc)Fe]_2_O (**c** and **g**), and [(OEP)Fe]_2_O (**d** and **h**). In the N 1s spectra, pyridinic and bridging nitrogens are depicted in blue, metal-coordinated nitrogens are depicted in magenta, graphitic/pyrrolic nitrogens are depicted as yellow, oxidized nitrogens are shown in purple and the π–π* satellites are shown in dark cyan. In the Fe 2p spectra, the 2p_3/2_ Fe(II) peak is shown in blue and the 2p_3/2_ Fe(III) peak is given in magenta. The Fe 2p_1/2_ peak is given in aqua blue. Multiplet peaks for the 2p_3/2_ and 2p_1/2_ peaks are given in green and dark cyan, respectively.

The nitrogen environments present in Fe-N-C, [(phen_2_N_2_)Fe]_2_O, (phen_2_N_2_)H_2_, [(Pc)Fe]_2_O, and [(OEP)Fe]_2_O were analyzed by XPS (see Supplementary Figs. 9 and 10 for XPS survey spectra of all compounds and Supplementary Tables 3–5 for N:Fe ratios of all samples, component decomposition of the N 1s manifold for Fe-N-C, and N 1s peaks, assignments, and parameters for all samples). For Fe-N-C, we observed a broad N 1s XPS signal similar to that reported for other Fe-N-C materials (Fig. 3a). The N peak envelope for Fe-N-C spans 396–407 eV and can be deconvoluted into four peaks, previously assigned to oxidized (404.0 eV), graphitic/pyrrolic (401.3 eV), metal-coordinated (399.9 eV), and pyridinic nitrogen (398.3 eV)^60,61^. We note that since this sample was prepared by the pyrolysis of ZIF-8, we expect the metal-coordinated N peak to result from both iron and zinc coordination. Notably, comparing N 1s XPS binding energies for Zn^II^ porphyrins^62^ and Fe^III^ porphyrins (Fig. 3d and Supplementary Fig. 11) reveal N XPS peaks within 0.2 eV, suggesting that residual Zn does not dramatically convolute the N XPS envelope of this Fe-N-C preparation.

The broad peak envelope for Fe-N-C contrasts with the well-defined nitrogen environments in [(Pc)Fe]_2_O, [(OEP)Fe]_2_O, and [(phen_2_N_2_)Fe]_2_O. For [(phen_2_N_2_)Fe]_2_O, high-resolution N 1s spectra (Fig. 3b) reveal a single peak centered at 399.3 eV that can be deconvoluted into two components centered at 398.3 and 399.6 eV with a ratio of peak area values of 1:2. Based on the integration ratio and the symmetry of the (phen_2_N_2_)Fe subunits in [(phen_2_N_2_)Fe]_2_O, we assign the 398.3 peak to the bridgehead nitrogens and the 399.6 peak to the coordinated pyridinic nitrogens. In comparison, the N 1s XPS spectrum (Supplementary Fig. 12, bottom) of (phen_2_N_2_)H_2_ is dominated by two peaks corresponding to bridging and pyridinic nitrogen atoms at 397.9 and 399.5 eV, respectively, in a 1:2 ratio. This indicates that both bridging and pyridinic nitrogen atoms shift to higher binding energies upon metalation with iron. Similar changes in the N 1s spectrum have been reported for free-base porphyrins upon metalation^63^. In contrast, the XPS N 1s spectra of [(Pc)Fe]_2_O and [(OEP)Fe]_2_O differ markedly from that of [(phen_2_N_2_)Fe]_2_O. The spectrum of [(Pc)Fe]_2_O is composed of a sharp peak at 398.4 eV with a satellite peak centered at 400.1 eV (Fig. 3c). Analogously, [(OEP)Fe]_2_O consists of a sharp peak at 397.6 eV with a broad satellite peak centered at 399.9 eV^64,65^ (Fig. 3d). While the main peak in [(Pc)Fe]_2_O appears in the region assigned to N_pyridinic_ for Fe-N-C, it is best fit with a single component, indicating that the coordinating and non-coordinating N atoms have comparable binding energies. Thus, the exclusive presence of phthalocyanine environments in Fe-N-C would be insufficient to account for the large metal-N_x_ peak in Fe-N-C. Notably, N 1 s peaks for (phen_2_N_2_)FeCl, (Pc)FeCl, and (OEP)FeCl (Supplementary Fig. 11) appear within 0.1–0.4 eV of the corresponding peaks for the μ-oxo compounds, indicating that the identity of the axial ligand does not impact the N 1s binding energy to the same extent as does changing the metal-coordinated nitrogen atoms from pyrrolic to pyridinic. Importantly, the N 1s peaks for the coordinated pyridinic nitrogens in the (phen_2_N_2_)Fe complexes match well with the peak corresponding to the metal-coordinated pyridinic nitrogen component (399.9 eV) of Fe-N-C, whereas the N 1s peaks of the coordinated pyrrolic nitrogens in (Pc)Fe and (OEP)Fe complexes appear at significantly lower binding energies. Together, the XPS data indicate that the iron-coordinated N environment in [(phen_2_N_2_)Fe] species are electronically very similar to the metal-coordinated N environments in Fe-N-C materials.

The iron environments present in Fe-N-C, [(phen_2_N_2_)Fe]_2_O, [(Pc)Fe]_2_O, and [(OEP)Fe]_2_O were also examined by XPS (Supplementary Table 6). Consistent with previously reported data for Fe-N-C materials^60,66^, we observe Fe 2p_3/2_ peaks at 709.5 and 713.9 eV (Fig. 3e) assigned to Fe(II) and Fe(III) formal oxidation states, respectively (Supplementary Table 7)^60^. In contrast, [(Pc)Fe]_2_O (Fig. 3g) exhibits Fe 2p_3/2_ and 2p_1/2_ peaks at 708.9 and 722.0 eV, respectively, and [(OEP)Fe]_2_O (Fig. 3h) displays Fe 2p_3/2_ and 2p_1/2_ peaks at 709.2 and 723.2 eV, respectively. As reported for iron porphyrin and phthalocyanine samples, we also observe significant asymmetry on the main Fe 2p_3/2_ peak for both [(OEP)Fe]_2_O and [(Pc)Fe]_2_O compounds that is attributed to multiplet splitting arising from core-hole interactions with the open-shell electronic structure of the high-spin ferric centers^67,68^. Similar features are observed for (OEP)FeCl and (Pc)FeCl, albeit with a ~0.5–1.5 eV shift to higher binding energy (Supplementary Figs. 13 and 14). These XPS features are distinct from those observed for [(phen_2_N_2_)Fe]_2_O (Fig. 3f) which displays Fe 2p_3/2_ and 2p_1/2_ peaks at 710.8 and 724.3 eV. We note that (phen_2_N_2_)FeCl shows Fe 2p_3/2_ and 2p_1/2_ peaks at similar binding energies, 710.5 and 723.9 eV, respectively (Supplementary Fig. 15), and that residual FeCl_3_ salts are likely subsumed into these peaks^69^. As with [(OEP)Fe]_2_O and [(Pc)Fe]_2_O, we also observe significant asymmetry in the Fe 2p_3/2_ and Fe 2p_1/2_ peaks of [(phen_2_N_2_)Fe]_2_O that we also attribute to core-hole interactions. Importantly, we observe a ~1.5–1.9 eV shift to higher binding energy for the main Fe 2p_3/2_ peak for [(phen_2_N_2_)Fe]_2_O relative to both [(OEP)Fe]_2_O and [(Pc)Fe]_2_O. This observation is consistent with increased π-acidity of the pyridinic macrocycle in [(phen_2_N_2_)Fe]_2_O, which withdraws electron density from the iron center and is mirrored by the positions of the Fe–O–Fe peaks evident in the high-resolution O 1 s spectra of the μ-oxo complexes (Supplementary Fig. 16 and Supplementary Table 8). Notably, the peak in Fe-N-C assigned to Fe(III) is 2.7 eV positive of the corresponding Fe(III) peak for [(phen_2_N_2_)Fe]_2_O but even more positive than the same peak in [(OEP)Fe]_2_O and [(Pc)Fe]_2_O. These data suggest that while the Fe(III) centers in Fe-N-C may exist in an even more withdrawing ligation environment, a pyridinic macrocycle complex such as [(phen_2_N_2_)Fe]_2_O provides a better structural model for Fe-N-C than pyrrolic macrocycles such as [(OEP)Fe]_2_O and [(Pc)Fe]_2_O. Indeed, the strongly electron-withdrawing environment in the material helps explain the positive shift of the putative Fe(II) component of Fe-N-C, which makes it appear close in energy to the main Fe 2p_3/2_ peaks of the Fe(III) complexes. Overall, the XPS data are consistent with nitrogen and iron environments in [(phen_2_N_2_)Fe]_2_O that are more electropositive than the pyrrolic macrocycles and more similar to the metal-N_x_ sites in Fe-N-C.

### XAS results demonstrate a strong similarity with Fe-N-C

**Fig. 4:**
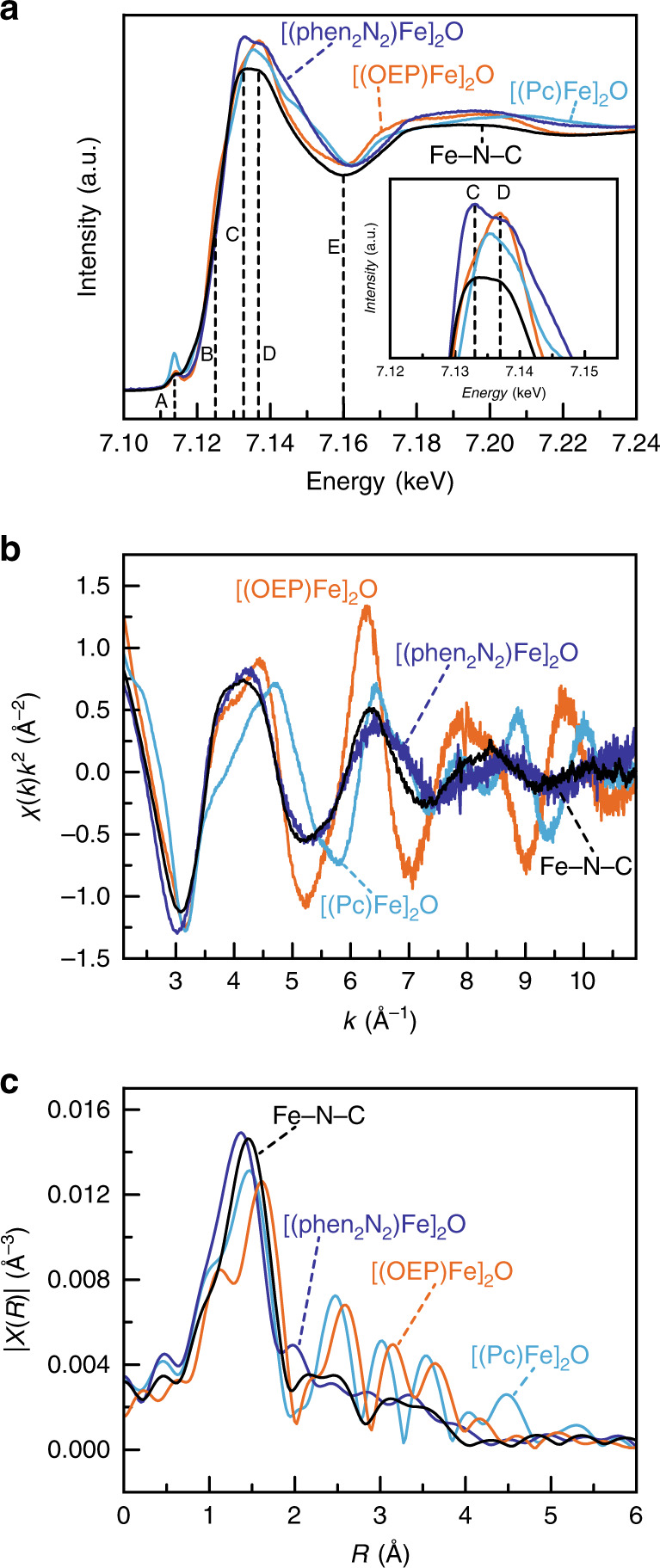
X-ray absorption spectroscopy data. XANES spectra (**a**) and expanded main-edge region (inset), *k*^2^-weighted Fe k-edge EXAFS spectra (**b**) and *k*^2^-weighted Fourier transform Fe EXAFS spectra (**c**). The energy values of the vertical lines and the alphabetical labels in (**a**) are taken from reference^70^. Fe-N-C is shown in black, [(phen_2_N_2_)Fe]_2_O in blue, [(OEP)Fe]_2_O in orange and [(PcFe)]_2_O in aqua.

We next examined the electronic structure and coordination environment of the iron centers in Fe-N-C, [(phen_2_N_2_)Fe]_2_O, [(OEP)Fe]_2_O, and [(Pc)Fe]_2_O by XAS. The XANES spectrum of our Fe-N-C preparation (Fig. 4a, black) matches those reported in the literature^70^. While a rigorous analysis of the X-ray absorption near edge structure (XANES) spectra for Fe-N-C materials is challenging due to the presumed presence of a mixture of Fe(II) and Fe(III) (Supplementary Table 7) as well as an unknown degree of heterogeneity in the local coordination environment of each iron, the XANES line shapes have been examined for a wide variety of pyrolyzed Fe-N-C catalysts, including Fe-N-C derived from iron pyrrolic macrocycles, and features of the XANES have been correlated to pyrolysis temperature and ORR activity^26,70,71^. In particular, the intensity of the first main-edge peak, C, has been found to positively correlate with increased pyrolysis temperature as well as improved ORR activity, whereas the intensities of the main-edge shoulder, B, and the higher energy main-edge peak, D, have been shown to anticorrelate with both pyrolysis temperature and ORR activity (Fig. 4a, dotted vertical lines)^70^. Notably, the XANES spectra of [(OEP)Fe]_2_O (Fig. 4a, orange) and [(Pc)Fe]_2_O (Fig. 4a, aqua) show enhanced intensity for D with [(OEP)Fe]_2_O also showing enhanced intensity for B. These features contrast with the XANES spectrum of [(phen_2_N_2_)Fe]_2_O (Fig. 4a, blue), which shows a higher intensity for C, and lower intensities for B and D. The intensity of pre-edge peak, A, has also been shown to positively correlate with ORR activity^70^. We find that the pre-edge peak line shape for [(phen_2_N_2_)Fe]_2_O matches closely with that of Fe-N-C, but that the peak intensity for both is lower than that for [(OEP)Fe]_2_O and dramatically lower than the sharp pre-edge feature observed for [(Pc)Fe]_2_O (Supplementary Fig. 17). Given the high sensitivity of the pre-edge peak to the identity of the axial ligand in similar tetrapyrrolic complexes^72^ as well as distortions in the Fe-N_4_ plane resulting from variation of the Fe-O-Fe angle in [(OEP)Fe]_2_O^55,73^ and [(Pc)Fe]_2_O^59^, we refrain from overinterpreting the pre-edge peak shapes or intensity^74^. Nonetheless, the suppressed intensity at B and D and increased intensity at C for [(phen_2_N_2_)Fe]_2_O suggest that the (phen_2_N_2_)Fe molecular analogue is a superior model for the active sites in Fe-N-C materials relative to pyrrolic macrocycles.

Extended X-ray absorption fine structure (EXAFS) data provide further evidence in support of the structural similarity between the iron coordination environments in [(phen_2_N_2_)Fe]_2_O and Fe-N-C. The *k*^2^-weighted EXAFS oscillations for Fe-N-C (Fig. 4b, black) exhibit remarkably similar amplitudes and periods to the EXAFS of [(phen_2_N_2_)Fe]_2_O (Fig. 4b, blue) out to ~10.0 Å^−1^ with only a small phase shift beyond ~6.0 Å^−1^. In contrast, the *k*^2^-weighted EXAFS for [(OEP)Fe]_2_O (Fig. 4b, orange) and [(Pc)Fe]_2_O (Fig. 4b, aqua) displays amplitudes and periods that differ dramatically from the EXAFS of [(phen_2_N_2_)Fe]_2_O and Fe-N-C beyond ~3.5 Å^−1^. For example, whereas traces for both Fe-N-C and [(phen_2_N_2_)Fe]_2_O pass through χ(*k*)*k*^2^ = 0 at eight different points between 2.5 and 10 Å^−1^, [(OEP)Fe]_2_O and [(Pc)Fe]_2_O do so nine and eleven times, respectively. Furthermore, the similarity between Fe-N-C and [(phen_2_N_2_)Fe]_2_O is also reflected in the *k*^2^-weighted Fourier transform EXAFS radial distribution spectra (Fig. 4c). The [(phen_2_N_2_)Fe]_2_O complex displays a broad primary coordination shell scattering peak at 1.36 Å apparent distance, similar to the first shell peak for Fe-N-C at 1.46 Å apparent distance. In contrast, the first shell scattering peaks for [(OEP)Fe]_2_O and [(Pc)Fe]_2_O appear at 1.62 and 1.47 Å apparent distance and display prominent shoulders at lower apparent distances that are not observed in Fe-N-C or [(phen_2_N_2_)Fe]_2_O. Likewise, both [(phen_2_N_2_)Fe]_2_O and Fe-N-C have no prominent scattering interactions beyond 2.25 Å, whereas significant  higher-order scattering is observed for [(OEP)Fe]_2_O and [(Pc)Fe]_2_O. Taken together, the raw EXAFS data for Fe-N-C mostly match the spectra of [(phen_2_N_2_)Fe]_2_O and are quite distinct from those of either [(OEP)Fe]_2_O or [(Pc)Fe]_2_O.

The EXAFS peaks for all four materials are well modeled with a five-coordinate Fe center bearing four equatorial Fe-N scatterers and one axial Fe-O scatterer (Supplementary Table 9). The fits (Supplementary Figs. 18 and 19) from modeling the data return Fe-N scattering paths of 1.94, 1.97, 2.06, and 1.94 Å for Fe-N-C, [(phen_2_N_2_)Fe]_2_O, [(OEP)Fe]_2_O, and [(Pc)Fe]_2_O, respectively. The Fe-N distances for both [(OEP)Fe]_2_O and [(Pc)Fe]_2_O agree with the corresponding distances extracted from crystal structures^55,75,76^. Importantly, these Fe–N bond lengths reflect not only the N–N separation in the ligand, but also the degree of pucker of the iron center out of the equatorial plane. Thus, this distance alone cannot distinguish pyridinic from pyrrolic ligation and, indeed, we see similar Fe–N vectors for [(phen_2_N_2_)Fe]_2_O and [(Pc)Fe]_2_O. Furthermore, for [(phen_2_N_2_)Fe]_2_O, the 1.97 Å Fe-N distance extracted from EXAFS modeling is substantially higher than the 1.86 Å distance predicted from the calculation if the iron were to reside in the N_4_ plane for the phen_2_N_2_ ligand (Supplementary Fig. 20), further evincing that the iron center is puckered. This reasoning is also in line with a computational XAS study that showed an enhancement of the main-edge peak C (Fig. 4a, blue) as the displacement of iron from the N_4_ plane was increased^70^. Consistent with the Fe-N-C material having a putative OH_x_ axial ligand, the EXAFS fits return an Fe–O scattering path of 2.04 Å, slightly larger than the range of Fe–O scattering paths, 1.73–1.79 Å, extracted for the molecular μ-oxo complexes.

Taking the raw *k*^2^-weighted EXAFS oscillations, the FT-EXAFS spectra, and the degree of iron puckering into account, the XAS data highlight that, relative to (Pc)Fe and (OEP)Fe, (phen_2_N_2_)Fe is an improved model of the iron sites in Fe-N-C catalysts. We stress that the similarity in the *k*-space EXAFS data should not be taken to mean that (phen_2_N_2_)Fe has an identical structure to Fe-N-C, but that the aggregate scattering environment of the iron centers in (phen_2_N_2_)Fe is qualitatively similar to the average cumulative scattering environment of the iron centers in Fe-N-C. We acknowledge this resemblance could be coincidental and introduced by countervailing changes in axial and equatorial scattering interactions. Nonetheless, the data indicate that the iron centers in (phen_2_N_2_)Fe exist in a qualitatively similar average environment to the iron centers in Fe-N-C.

### Fe(III/II) potentials are defined by ligating and proximal N atoms

All three model compounds as well as the Fe-N-C material were evaluated electrochemically as thin films supported on glassy carbon electrodes. In a typical preparation, Fe-N-C powders were dispersed in a 7:2:1 combination of CH_2_Cl_2_, ethanol, and 5 wt% Nafion solution (75 wt% ethanol and 20 wt% water), respectively. The resulting inks were dropcast onto electrodes and allowed to dry in air to generate a well-adhered catalyst film. A similar procedure was used for (phen_2_N_2_)FeCl, (OEP)FeCl, and (Pc)FeCl with inclusion of Vulcan carbon powder to enhance film conductivity. We expect that any residual FeCl_3_ salts present in the (phen_2_N_2_)FeCl sample will readily dissolve into the acidic electrolyte and will not impact the electrochemical results. In line with this expectation, we find that FeCl_3_/Vulcan inks are inactive for ORR (Supplementary Fig. 21).

**Fig. 5:**
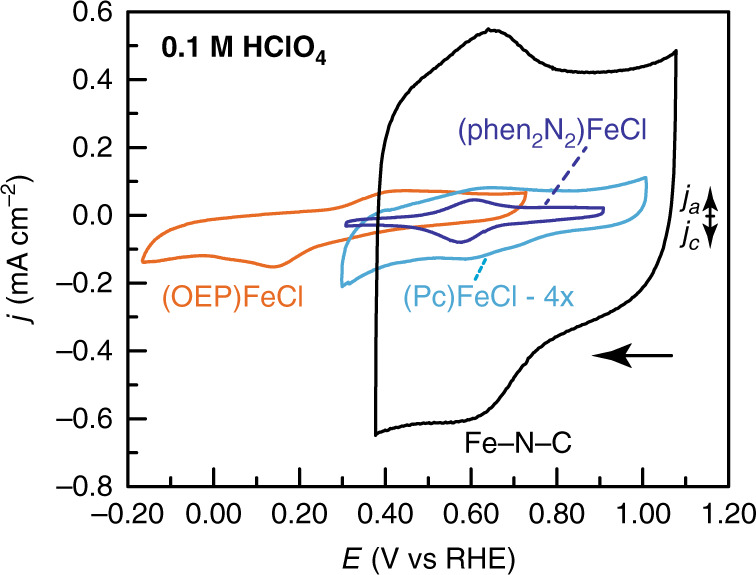
Cyclic voltammograms of adsorbed catalysts. Rotating glassy carbon disk electrodes are shown modified with catalyst films containing Fe-N-C (black), (phen_2_N_2_)FeCl (blue), (Pc)FeCl (aqua, 4× vertical expansion), and (OEP)FeCl (orange). Data were recorded at 5 mV s^−1^ and 2000 rpm rotation rate in O_2_-free 0.1 M HClO_4_ electrolyte.

Cyclic voltammograms of Fe-N-C, (phen_2_N_2_)FeCl, (OEP)FeCl, and (Pc)FeCl recorded in the absence of O_2_ provide insight into the redox potential of the metal center. For (OEP)FeCl (Fig. 5, orange), we observe a redox feature with *E*_1/2_ = 0.27 V (all potentials are reported vs the reversible hydrogen electrode, RHE). We note that the Fe(III/II) redox couple of (OEP)FeCl is quite broad with a peak-to-peak separation of 315 mV. We attribute this to sluggish Fe(III/II) redox self-exchange through the film, but nonetheless, we note that the observed *E*_1/2_ value is comparable to other carbon-adsorbed iron porphyrin compounds^17,56,77,78^. For (Pc)FeCl (Fig. 5, aqua), we observe a broad redox feature with *E*_1/2_ = 0.61 V (Supplementary Fig. 22), similar to reported values for carbon-supported iron phthalocyanine complexes^79^. For (phen_2_N_2_)FeCl (Fig. 5, blue), we observe a reversible redox wave at 0.59 V. Based on the known Nernstian pH dependence of Fe(III/II) couples for adsorbed porphyrin complexes^80^ as well as the quasi-Nernstian behavior of the redox wave for (phen_2_N_2_)Fe (Supplementary Fig. 23), we assign this redox process to the one-electron, one-proton reduction of Fe(III)-OH to Fe(II)-OH_2_.

For Fe-N-C (Fig. 5, black), we observe a large double-layer capacitance, consistent with the high surface area of the material, and a pronounced redox wave at 0.63 V (Fig. 5). Although in situ spectroscopic data are required for an unambiguous assignment, we tentatively attribute this redox wave to proton-coupled electron transfer (PCET) reactions of edge-localized quinone and hydroxyl moieties, rather than to an Fe(III/II) redox process. Although this assignment may seem counterintuitive, we favor it for several reasons. First, quinone and hydroxyl PCET reactions are known to occur in the ~0.6 V potential region on carbon powders^81^. Second, integrating the observed 0.6 V redox wave yields a charge that is 3.4 times larger than the expected charge for a 1-electron redox process of the surface exposed iron centers in the Fe-N-C material^82^. Third, the potential of the wave is comparable to the value for (Pc)Fe and (phen_2_N_2_)Fe and this is inconsistent with the expectation that the extended π framework in Fe-N-C should raise the Fe(III/II) potential^83^. Fourth, Fe-N-C catalyzes ORR at potentials ~0.2 V positive of the observed redox wave. Thus, if the observed 0.6 V redox wave were assigned to the Fe(III/II) couple, the equilibrium fraction of catalytically active Fe(II)-N_4_ sites at catalytic onset would be very small. Finally, other reports have observed waves for Fe-N-C materials at ~0.8 V^84–86^, in line with the onset of ORR catalysis at Fe-N-C materials (see below). We postulate that the actual Fe(III)-OH/Fe(II)-OH_2_ redox process for this Fe-N-C material is obscured by the large double-layer charging background and the likely distribution of iron redox potentials. We also note that some iron redox centers may not engage in PCET reactivity and would not be expected to display a redox wave^87^.

The redox potentials  extracted from the model compounds provide valuable information about the factors that may contribute to the redox potentials of the iron centers in Fe-N-C materials. Despite the fact that both (Pc)Fe and (OEP)Fe have pyrrolic ligating atoms, the redox potential for (Pc)Fe is ~0.3 V positive of the redox potential of (OEP)FeCl. The principle structural differences between (Pc)Fe and (OEP)Fe are the presence of N vs C atoms in the meso positions and the presence of a more extended π system in (Pc)Fe. One study has found that iron octaethyltetraazaporphyrin possesses a similar Fe(III/II) redox potential to (Pc)Fe^88^, suggesting that the presence of electronegative meso-N atoms is the principal contributor to the dramatic positive shift observed. Notably, whereas (phen_2_N_2_)FeCl possesses two non-ligating nitrogen atoms in the local environment, (Pc)FeCl has four, yet their Fe(III/II) potentials are comparable. This highlights that the less electron-donating pyridinic coordination environment in (phen_2_N_2_)FeCl is an ideal structural motif for pushing the Fe(III/II) positive. Together, the data suggest that for the iron centers in the Fe-N-C material, the effective redox potential is due, not only to the ligating heterocycles, but also the nitrogen population near the Fe center^16,89,90^. Thus, even Fe-N_4_ sites with identical primary coordination spheres may display a distribution of redox properties depending on the population and position of non-ligating proximal nitrogen atoms.

The observed Fe(III/II) redox waves result from only a fraction of the catalyst loaded into the dropcast film. The integrated charge in the Fe(III/II) redox waves of (phen_2_N_2_)FeCl, (OEP)FeCl, and (Pc)FeCl catalyst film corresponds to 7%, 43%, and 2% of the total catalyst loading for each compound, respectively (Supplementary Table 10). We attribute the higher electroactive percentage in (OEP)FeCl film to the improved solubility of the porphyrin catalyst, which leads to superior catalyst dispersion within the film. Nonetheless, these PCET redox potentials provide a good indication that the iron centers in (phen_2_N_2_)Fe and (Pc)Fe are more electropositive than those in (OEP)Fe.

### (Phen_2_N_2_)FeCl catalyzes ORR with similar activity to (Pc)FeCl

**Fig. 6:**
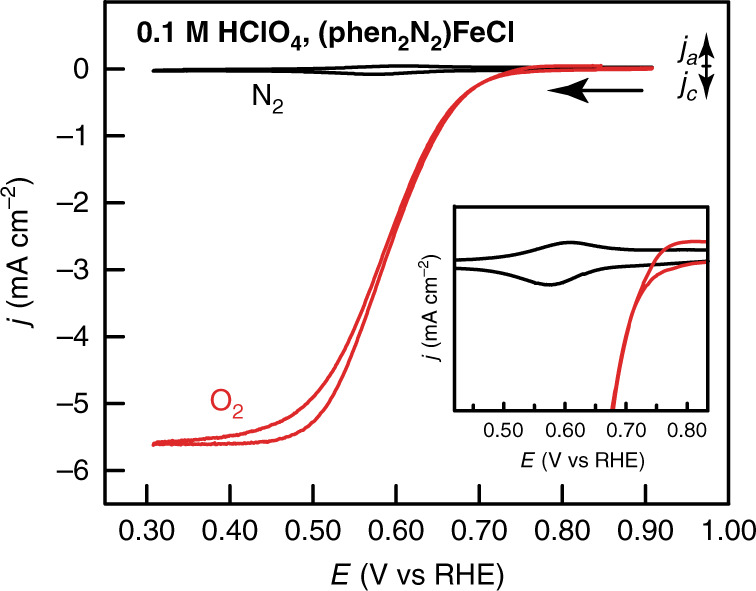
Cyclic voltammograms of adsorbed (phen_2_N_2_)FeCl. A rotating glassy carbon disk electrode modified with a catalyst film containing (phen_2_N_2_)FeCl in N_2_- (black) and O_2_- (red) saturated 0.1 M HClO_4_ aqueous electrolyte. Data were recorded at 5 mV s^−1^ and 2000 rpm rotation rate.

**Fig. 7:**
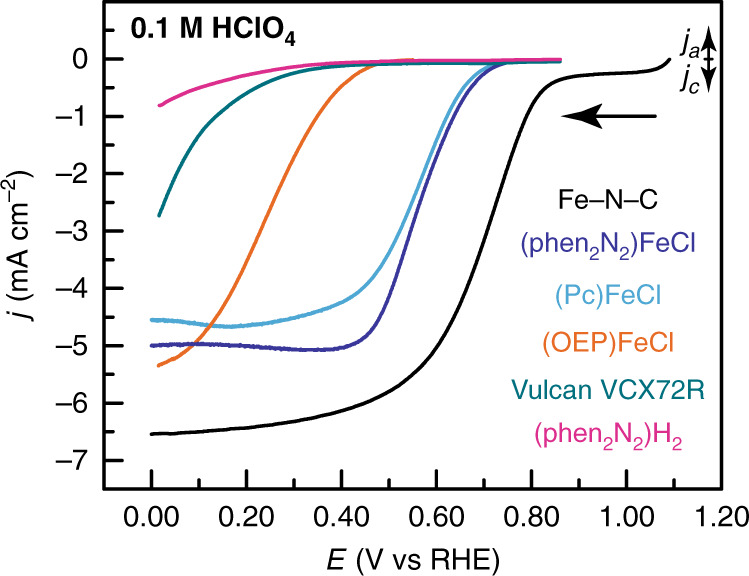
Linear sweep ORR voltammograms of adsorbed catalysts. Glassy carbon disk electrodes modified with catalyst films containing Fe-N-C (black), (phen_2_N_2_)FeCl (blue), (Pc)FeCl (aqua), (OEP)FeCl (orange), Vulcan carbon (green), and (phen_2_N_2_)H_2_ (magenta) in O_2_-saturated 0.1 M HClO_4_ electrolyte. Data were recorded at 5 mV s^−1^ and 2000 rpm rotation rate.

(Phen_2_N_2_)FeCl is a potent catalyst for the ORR. In O_2_-saturated 0.1 M HClO_4_, the reversible surface redox wave at 0.59 V (Fig. 6, black) is replaced by a large catalytic wave that displays an onset potential of 0.75 V (all onset potentials correspond to a current density of –0.1 mA cm^−2^ and all electrochemical performance parameters are collected in Supplementary Table 11) and reaches a current plateau at ~0.5 V (Fig. 6, red). This catalytic wave spans the same potential range as the reversible redox feature in the absence of O_2_ suggesting that ORR catalysis is mediated by the Fe(III/II) redox process (Fig. 6, inset). We also find that the Fe center is critical for activity. The unmetalated (phen_2_N_2_)H_2_ ligand has activity comparable to the carbon background (Fig. 7, green) in acidic media with an onset potential of 0.34 V (Fig. 7, magenta) and activity slightly higher than background in alkaline media with an onset potential of 0.75 V, 140 mV negative of (phen_2_N_2_)FeCl (Supplementary Figs. 24 and 25). Catalytic onset for (phen_2_N_2_)FeCl (Fig. 7, blue) is 320 and 30 mV positive of the onset observed for (OEP)FeCl and (Pc)FeCl, respectively. Catalytic activity is improved in basic media with an onset of 0.89 V in 0.1 M KOH (Supplementary Fig. 24, blue) for (phen_2_N_2_)FeCl, similar to the onset observed for (Pc)FeCl and 150 mV positive of the onset for (OEP)FeCl.

The above qualitative activity trends are also reflected in the per site TOF values for the three model complexes. TOF values, in electrons passed per site per second, were extracted as a function of potential by dividing the current density by the integrated charge passed in the Fe(III/II) redox feature. In accordance with the raw voltammogram data in Fig. 6, (phen_2_N_2_)FeCl displays a 1000-fold higher TOF than (OEP)FeCl in the activation-controlled region at 0.55 V in 0.1 M HClO_4_, but comparable TOF values to that of (Pc)FeCl (Supplementary Fig. 26a and Supplementary Table 12). In 0.1 M NaOH electrolyte, (phen_2_N_2_)FeCl and (Pc)FeCl display TOF values that are 280- and 1100-fold higher than (OEP)FeCl at 0.80 V (Supplementary Fig. 26b and Supplementary Table 12).

This positive shift in onset potential and the corresponding increase in TOF for (phen_2_N_2_)FeCl and (Pc)FeCl relative to (OEP)FeCl is consistent with the positive shift of the corresponding Fe(III/II) redox potentials for the former compounds. These results are in line with prior work that identified scaling relationships in ORR activity among adsorbed porphyrins and phthalocyanines, and showed that catalytic activity is positively correlated with the Fe(III/II) potential^91,92^. While a detailed mechanistic analysis of the ORR profile of these catalysts is beyond the scope of this work, the observed correlation among adsorbed Fe-N_4_ compounds could be rationalized by the following simplified mechanistic sequence:

1 $${\mathrm{Fe}}^{III} - {\mathrm{OH}} + e^ - + {\mathrm{H}}^ + \,\rightleftarrows\, {\mathrm{Fe}}^{II} - {\mathrm{OH}}_2\quad \quad K_1$$

2 $${\mathrm{Fe}}^{II}-{\mathrm{OH}}_2 + {\mathrm{O}}_{2} \to {\mathrm{Fe}}^{III} - {\mathrm{O}}_2 + {\mathrm{OH}}_2\quad \quad k_2\quad {\mathrm{RLS}}$$

In this sequence, at any given *E*, the rate of the reaction will be given by the product, *K*_1_ × *k*_2_, of the equilibrium constant, *K*_1_, for the Fe(III)-OH to Fe(II)-OH_2_ conversion, Eq. (1), and the rate constant, *k*_2_, for rate-limiting O_2_ binding, Eq. (2). If *k*_2_ is roughly insensitive to the local structure of the Fe center, then the reaction rate will be predominantly gated by the Fe(III/II) redox process and the higher redox potentials will correlate with more available Fe(II) at a given *E*, resulting in faster catalysis. We acknowledge that the above mechanistic picture is speculative and will require more studies to validate, but it nonetheless provides a simple framework for rationalizing the observed reactivity trend. Furthermore, our data provide the first experimental evidence that changing from pyrrolic to pyridinic coordination does not dramatically alter the scaling relationship between ORR activity and Fe(III/II) potential. This provides further support for the notion that Fe-N-C materials contain pyridinic Fe-N_4_ sites with sufficiently high redox potentials to mediate ORR at relatively low overpotentials.

The electrocatalytic data establish that (phen_2_N_2_)FeCl and (Pc)FeCl have superior activity relative to (OEP)FeCl, whereas both remain at a deficit to Fe-N-C. While the Fe(III/II) redox potential is difficult to discern for Fe-N-C, the positive shift of the Fe(III) peak in the XPS suggests that the iron centers in Fe-N-C are even more electropositive than those in (phen_2_N_2_)FeCl and (Pc)FeCl. This change may contribute to the higher onset potentials of 0.85 and 0.90 V observed for the Fe-N-C catalyst in acidic and alkaline media, respectively^17^. We stress that although (Pc)FeCl and (phen_2_N_2_)FeCl display comparable activity for ORR, (Pc)FeCl is an inferior structural model on the basis of the spectroscopic results presented above. In aggregate, the spectroscopic as well as electrochemical results strongly suggest that (phen_2_N_2_)FeCl is the more effective model complex for mimicking iron-containing active sites in Fe-N-C relative to pyrrolic complexes such as (Pc)FeCl. Furthermore, it follows that observing high catalytic ORR performance is insufficient evidence to establish a molecular complex as an accurate and comprehensive molecular model for the active sites in Fe-N-C materials. The deficit in activity relative to Fe-N-C may also result, in part, from weak interaction between the molecular orbitals of the model and the band states of the carbon host. Notwithstanding, the fact that a discrete pyridinic molecular complex such as (phen_2_N_2_)FeCl displays an ORR onset within 150 mV of champion Fe-N-C materials suggests enormous opportunities for rational molecular design of highly active Fe-based ORR catalysts based on pyridinic ligation motifs that structurally model Fe-N-C.

### (Phen_2_N_2_)FeCl mirrors the selectivity trends of Fe-N-C

**Fig. 8:**
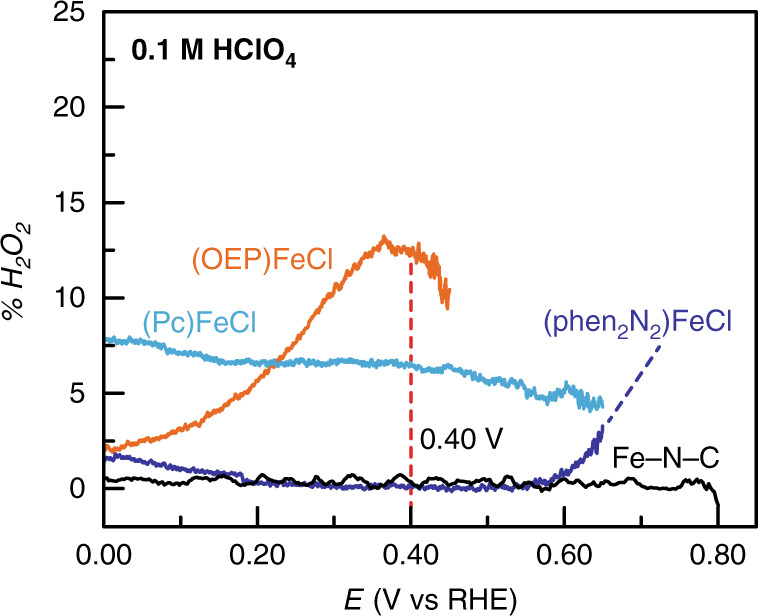
%H_2_O_2_ vs potential for adsorbed catalysts. All data were recorded in O_2_-saturated 0.1 M HClO_4_ electrolyte at 5 mV s^−1^ and 2000 rpm rotation rate. (phen_2_N_2_)FeCl is shown in blue, (OEP)FeCl is shown in orange, (Pc)FeCl is shown in aqua, and Fe-N-C is shown in black.

Furthermore, (phen_2_N_2_)FeCl displays high selectivity for the four-electron reduction of O_2_ that is comparable to Fe-N-C and greater than that of (OEP)FeCl or (Pc)FeCl. Using rotating ring disk electrode (RRDE) voltammetry (Supplementary Figs. 27 and 28) we determined the percent H_2_O_2_ produced and electrons transferred as a function of applied potential for all four catalysts (Fig. 8 and Supplementary Figs. 29 and 30). For (phen_2_N_2_)FeCl, H_2_O_2_ production stabilizes rapidly, reaching values of 0.1% and 2.7% in acid and alkaline media at 0.40 V, respectively. These values correspond to 3.99 and 3.95 electrons transferred in each condition, respectively. Indeed, in both acidic and alkaline media, the selectivity of (phen_2_N_2_)FeCl in the transport-limited region surpasses that of Fe-N-C at the same potential. Further, in stark contrast to (phen_2_N_2_)FeCl, both (Pc)FeCl and (OEP)FeCl display significantly lower selectivity in acid media, generating 6.6% and 12.5% H_2_O_2_, respectively, at 0.40 V. In 0.1 M NaOH, (Pc)FeCl displays similar selectivity relative to (phen_2_N_2_)FeCl; however, both are slightly more selective than (OEP)FeCl (Supplementary Fig. 29). We stress that the RRDE results are best interpreted in qualitative rather than quantitative terms because apparent H_2_O_2_ yields are strongly dependent on catalyst loading, film morphology, ink composition, and carbon support identity^93–95^. Nevertheless, these results highlight the ability of a carbon-adsorbed pyridinic Fe-N_4_ coordination environment to facilitate selective O_2_-reduction pathways in both acidic and alkaline media.

Whereas Fe-N-C materials are relatively stable, we observe limited stability of the (phen_2_N_2_)FeCl catalyst with activity decaying in acidic media over the course of several slow-scan cyclic voltammograms (15–20 min). Analogous deactivations are also observed for the pyrrolic complexes and are documented in the literature^96–98^. These observations highlight the important role of the carbon framework in increasing the relative stability of Fe-N_4_ sites in Fe-N-C materials against oxidative and protolytic decomposition induced by the acidic conditions and the presence of parasitic amounts of H_2_O_2_^99^. Indeed, we posit that extending the aromatic periphery around the (phen_2_N_2_)FeCl active site could enhance stability and allow for the bottom-up synthesis of robust Fe-based ORR catalysts.

The results reported herein provide a molecular perspective on the structure and oxygen reduction reactivity of Fe-N_4_ active sites in Fe-N-C materials. We have synthesized a new (phen_2_N_2_)Fe fragment and demonstrated that it is a superior molecular model for the putative Fe-N_4_ active sites in Fe-N-C materials compared to exhaustively studied iron porphyrin and phthalocyanine complexes. Electrochemical studies establish that ORR activity is positively correlated to the Fe(III/II) potential and that, while (OEP)Fe has too low of a potential to mediate efficient catalysis, both phthalocyanine- and pyridine-based ligation environments in (Pc)Fe and (phen_2_N_2_)Fe can generate a positive shift of the Fe(III/II) potential and a corresponding increase in ORR activity. Among the model complexes, the (phen_2_N_2_)Fe platform is unique in displaying both high activity and near 100% selectivity for four-electron oxygen reduction in acidic media, the very characteristics found in Fe-N-C materials. Since high electrochemical performance does not necessarily imply structural similarity, our spectroscopic results provide additional discrimination. In particular, the pyridinic ligation environment in (phen_2_N_2_)Fe leads to XPS and XAS signatures that are remarkably similar to those of Fe-N-C materials and notably distinct from those in either (OEP)Fe or (Pc)Fe. Taken in aggregate, the spectroscopic similarity and the electrochemical activity and selectivity trends indicate that, relative to (OEP)Fe or (Pc)Fe, (phen_2_N_2_)Fe is a significantly improved structural and functional model of Fe-N-C materials. Since the (phen_2_N_2_)Fe core can be readily derivatized^100,101^ and used as the building block for functional catalytic materials, the molecular model complex developed here provides a powerful platform with which to advance the synthesis and understanding of single-site heterogeneous electrocatalysts for critical energy conversation reactions.

## Methods

### General synthetic considerations

Where indicated, synthetic procedures were carried out under an inert atmosphere in a nitrogen-filled glovebox. All other synthetic transformations were carried out on the benchtop without effort to exclude dioxygen or moisture. Anhydrous 1,10-phenanthroline (99%), 1,3-dibromopropane (98%), *tert*-butanol (99%), phosphorus pentachloride (98%), and phthalonitrile (98%) were obtained from Alfa Aesar and used as received. Potassium *tert*-butoxide (97%) and phosphoryl chloride (99%) were obtained from TCI and Sigma Aldrich, respectively and used as received. Iron(III) chloride (98%) was purchased from Strem Chemicals and stored in a nitrogen-filled glovebox. Compressed anhydrous ammonia (99.995%) was purchased from Airgas. *N*,*N*-dimethylformamide was deoxygenated and dried using a Glass Contour System (SG Water USA, Nashua, NH) and stored in the glovebox over 4 Å molecular sieves. D_2_O, CDCl_3_ and *d*-TFA were purchased from Sigma-Aldrich or Cambridge Isotope Laboratories and were used without further purification. 2, 3, 7, 8, 12, 13, 17, 18-Octaethyl-21*H*, 23*H*-porphine iron(III) chloride, (OEP)FeCl, was purchased from Frontier Scientific and used without further purification. Iron(II) phthalocyanine for use as a model complex in XAS data analysis was purchased from Alfa Aesar and sublimed at 600 °C under reduced pressure in a single-zone tube furnace for 3 h. Routine NMR spectra were recorded on Varian Mercury 300, Bruker Avance III 400, and Varian Inova 500 spectrometers. Chemical shifts for ^1^H and ^13^C{^1^H} spectra are reported in ppm downfield of TMS, with spectra referenced using the chemical shifts of the solvent residuals. Spectra recorded in *d*-TFA were referenced to a benzene solvent residual by including a small capillary of C_6_D_6_ in the sample tube. UV–Visible spectra were measured in a 1-cm quartz cell on a Varian Cary 50 spectrophotometer. MALDI-TOF mass spectra were obtained using Bruker Autoflex LRF and Bruker Omniflex instruments operating in reflectron mode, and the peaks reported are the mass of the most intense peak in the isotope envelope. MALDI-TOF samples consisted of analyte mixed with 1,4-bis(5-phenyl-2-oxazolyl)benzene (POPOP) as a desorption matrix. Elemental analyses and inductively coupled plasma mass spectra (ICP-MS) were performed by Robertson Microlit Laboratories (Ledgewood, NJ).

### Synthesis of Fe-N-C

Fe-N-C was synthesized by a hybrid method adapted from previously published procedures^19,48^. 0.019 g (0.11 mmol), Fe(OAc)_2_, 0.206 g (1.14 mmol), 1,10-phenanthroline, and 0.804 g (3.53 mmol) ZIF-8 MOF were mixed together in an agate mortar for 15 min. To remove excess water and oxygen, the prepyrolysis mixture was placed under vacuum for 30 min at 90 °C and subsequently held under vacuum for a further 12 h at room temperature. 0.856 g of the precatalyst powder was weighed into an alumina boat which was positioned at the midpoint of an alumina tube centered in a single-zone tube furnace. The alumina tube was sealed with steel endcaps, evacuated briefly, and then continuously purged with argon. The furnace was heated to 1000 °C at a ramp rate of 10 °C min^−1^ and maintained at 1000 °C for 60 min. The furnace was then allowed to return to room temperature. Subsequently, the alumina tube was evacuated briefly and then continuously purged with 5% hydrogen in argon. The furnace was ramped to 800 °C at a rate of 10 °C min^−1^ and maintained at 800 °C for 30 min. The sample was then allowed to return to room temperature. The resulting crude Fe-N-C powder was then sonicated in 30 mL of 0.1 M H_2_SO_4_ for 60 min. The black suspension was filtered and washed with water until the eluant pH was neutral. The sample was subsequently washed with acetone to remove excess water and the product was dried overnight at 60 °C to yield 219.2 mg of Fe-N-C as a black powder. ICP-MS: 1.07% Fe. ^57^Fe Mössbauer (90 K): [D1: (δ = 0.46 mm s^−1^, |Δ*E*_Q_| = 1.08 mm s^−1^, 71%), D2: (δ = 0.37 mm s^−1^, |Δ*E*_Q_| = 3.06 mm s^−1^, 29%)].

### Synthesis of 6,7-dihydro-5*H*-1,4-diazepino[1,2,3,4-*Imn*][1,10]phenanthroline-4,8-diium bromide (2)

Compound **2** was synthesized via alkylation of 1,10-phenanthroline using an excess of 1,3-dibromopropane in refluxing toluene. 10.787 g (59.86 mmol) 1,10-phenanthroline was suspended in 105 mL toluene. The mixture was stirred and heated to 70 °C and, subsequently, 28 mL (55.47 g, 275 mmol, 4.59 equiv.) 1,3-dibromopropane was added. The temperature was increased to 120 °C and the mixture was refluxed for 4 h. After allowing the reaction vessel to cool, the mixture was filtered and washed with hexanes to yield 18.688 g (82%) of compound **2** as a yellow powder. The observed NMR peaks in D_2_O matched the literature^102^.

### Synthesis of 6,7-dihydro-5*H*-1,4-diazepino[1,2,3,4-*Imn*][1,10]phenanthroline-3,9-dione (3)

Compound **3** was prepared using the modified workup described in the literature^103^. 5.061 g (13.24 mmol) of compound **2** was suspended in 85 mL *tert*-butanol and sonicated for 15 min. The mixture was then stirred while 6.026 g (53.7 mmol, 4.06 equiv.) KO^*t*^Bu was added in small portions over the course of 15 min. The reaction mixture was then heated to 40 °C and stirred for 12 h. The solvent was removed by rotary evaporation and the crude material was suspended in 100 mL of water. The product was extracted with 100 mL of chloroform and 5% methanol by volume was added to the chloroform solution. The mixture was then filtered through a plug of silica on a glass frit and the silica was washed with chloroform until the eluant was colorless. The combined washings were concentrated to dryness by rotary evaporation to yield 1746 g (52%) of compound **3** as a brown solid. The observed NMR peaks in CDCl_3_ matched those reported in the literature^102^.

### Synthesis of 2,9-dichloro-1,10-phenanthroline (4)

Under inert atmosphere, a mixture of 2.29 g (9.06 mmol) **3** and 3.77 g (18.1 mmol, 2.00 equiv.) PCl_5_ was suspended in 35 mL POCl_3_. The mixture was then refluxed under inert atmosphere for 14 h. After cooling to room temperature, the solution was poured onto 600 mL of ice and the resulting mixture was neutralized with aqueous ammonium hydroxide (30%) to a pH > 8. After neutralization, the resulting precipitate was collected by filtration. The solid was washed with water and dried overnight at elevated temperature to yield 1.99 g (89%) of compound **4** as a light brown powder. The observed NMR peaks in CDCl_3_ matched those given in the literature^102^.

### Synthesis of (phen_2_N_2_)H_2_

Preparation of (phen_2_N_2_)H_2_ has been previously described in the literature^45,49,104^, however an alternate method is presented here. 0.547 g (2.19 mmol) 2,9-dichlorophenanthroline was added to a cylindrical glass insert and enclosed at room temperature in a 100 mL Parr bomb equipped with a rupture disk. The bomb was purged with nitrogen for 10 min and was then pressurized with anhydrous ammonia at 114 psi. The bomb was then immersed in a bed of aluminum beads and heated to 280–300 °C with a heating mantle for 24 h. Following additional heating for 12 h at 300 °C, the bomb was allowed to return to room temperature. The residual ammonia was vented and the crude product was dissolved in a mixture of trifluoroacetic acid, acetic acid and methanol and stirred for 30 min. The mixture was neutralized and basified with 4 M NaOH and was stirred for an additional 12 h. The precipitate was filtered and sequentially washed with ethanol and chloroform until the washings were colorless. The isolated material was then dried at 60 °C for 12 h. The filtrate was concentrated in vacuo and heated to 300 °C under inert atmosphere for a period of 24 h in a 500 mL round bottom flask affixed to a reflux condenser. After cooling to room temperature, the solid derived from the filtrate was treated with an identical dissolution, precipitation, and washing sequence as before to yield a second crop. In total, 0.280 g (0.506 mmol, 66% combined yield) of 3bH,10bH-1,14:7,8-diethenotetrapyrido[2,1,6-*de*:2′,1′,6′-*gh*:2″,1″,6″-*kl*:2″′,l″′,6″′-*na*][1,3,5,8,10,12]hexaazacyclotetradecine, (phen_2_N_2_)H_2_, was isolated as a brown solid. ^1^H NMR (C_6_D_6_ in *d*-TFA): δ 9.41 (d, 4H, 10 Hz), 8.76 (s, 4H), 8.49 (d, 4H, 10 Hz). MALDI− MS (POPOP matrix): *m/z* 386.03 ([M]^+^ calc. for C_24_H_14_N_6_: 386.13). UV–Vis (DMSO): *λ*_max_ = 287 nm (ε = 23,419 L mol^−1^ cm^−1^), 322 (8,119), 339 (6,054), 360 (4,828), 376 (4,069), 439 (550), 467 (202). Anal. Calcd. for (phen_2_N_2_)H_2_•H_2_O (C_24_H_16_N_6_O_1_): C, 71.28; H, 3.99; N, 20.78. Found: C, 70.93; H, 3.47; N, 21.12.

### Synthesis of (phen_2_N_2_)FeCl

Under inert atmosphere, 0.060 g (0.155 mmol) (phen_2_N_2_)H_2_ was added to a pressure tube charged with a stirbar. 0.036 g (0.22 mmol, 1.4 equiv.) FeCl_3_ and 0.221 mL (0.930 mmol, 6.00 equiv.) N(*n*-Bu)_3_ were added to the vessel followed by 7 mL DMF. The tube was sealed and heated to 170 °C for 36 h with vigorous stirring. The temperature was allowed to return to ambient levels and the mixture was diluted with 20 mL CH_2_Cl_2_ under inert atmosphere. The mixture was then cooled overnight at −38 °C and the resulting precipitate was filtered and washed with copious amounts of ether and CH_2_Cl_2_ to yield 54 mg (73%) 1,14:7,8-Diethenotetrapyrido[2,1,6-*de*:2′,1′,6′-*gh*:2″,1″,6″-*kl*:2″′,l″′,6″′-*na*][1,3,5,8,10,12]hexaazacyclotetradecine iron(III) chloride, (phen_2_N_2_)FeCl. HR MALDI-TOF (POPOP matrix): *m/z* 440.1061 ([M – Cl]^+^, calc. for C_24_H_12_FeN_6_: 440.0473), 475.1023 ([M]^+^, calc. for C_24_H_12_ClFeN_6_: 475.0161). UV–Vis (DMSO): *λ*_max_ = 288 nm (ε = 11,649 L mol^−L^ cm^−c^), 302 (11,932), 319 (10,284), 338 (5,892), 366 (5,411), 400 (4,477), 429 (3,105), 459 (1,942). ^57^Fe Mössbauer (90 K): δ = 0.39 mm s^−1^, |Δ*E*_Q_| = 3.06 mm s^−1^. A satisfactory elemental analysis for (phen_2_N_2_)FeCl could not be obtained due the presence of unreacted Fe salts, primarily FeCl_3_ and Fe_2_Cl_6_•(DMF)_3_^50^. Fe_2_Cl_6_•(DMF)_3_ is synthesized by exposing FeCl_3_ as a solution in ether to DMF, resulting in a solid that can be collected by filtration. The reaction conditions described above should be conducive to forming Fe_2_Cl_6_•(DMF)_3_, which explains the origin of the minor impurity. Elemental analysis results matched upon the incorporation of 16% of Fe_2_Cl_6_•(DMF)_3_: Anal. Calcd. for (phen_2_N_2_)FeCl + 0.16(Fe_2_Cl_6_•(DMF)_3_ = (C_24_H_12_ClFeN_6_ + 0.16(C_9_H_21_Cl_6_Fe_2_N_3_O_3_): C, 53.30; H, 2.76; N, 15.89. Found: C, 52.96; H, 2.77; N, 15.93. This accounts for the additional doublet observed in the ^57^Fe Mössbauer spectrum (see Supplementary Fig. 5, middle) which is similar to that of hydrated FeCl_3_ adsorbed on carbon^105,106^. Exhaustive washing with common anhydrous solvents like ether and CH_2_Cl_2_ failed to remove residual Fe salts. This is attributed to the extreme insolubility of the complex that prevents the effective removal of trapped Fe salts. Indeed, even in highly polar solvents such as DMF, DMSO, and HMPA visible dissolution of (phen_2_N_2_)FeCl is only observed at temperatures in excess of ~150 °C.

### Synthesis of [(phen_2_N_2_)Fe]_2_O

In a typical preparation, a sample of (phen_2_N_2_)FeCl was placed in a cellulose extraction thimble and loaded into a Soxhlet apparatus. After placing the apparatus under inert atmosphere by purging extensively with argon, the sample was washed continuously with hot ethanol for 48 h. Afterwards, the sample was removed from the Soxhlet apparatus and dried in an oven overnight to remove trace water and volatile solvents, furnishing (*μ*-Oxo)bis[(1,14:7,8-Diethenotetrapyrido[2,1,6-*de*:2′,1′,6′-*gh*:2″,1″,6″-*kl*:2″′,l″′,6″′-*na*][1,3,5,8,10,12]hexa-azacyclotetradecine)iron(III)], [(phen_2_N_2_)Fe]_2_O. XPS N:Fe ratio: 6.3:1. ^57^Fe Mössbauer (90 K): δ = 0.45 mm s^−1^, |Δ*E*_Q_| = 0.87 mm s^−1^. HR MALDI-TOF (POPOP matrix): *m/z* 440.0975 ([M – C_24_H_12_FeN_6_O]^+^, calc. for C_24_H_12_FeN_6_: 440.0473). A satisfactory elemental analysis for [(phen_2_N_2_)Fe]_2_O could not be obtained due to the presence of trace impurities, which were presumably carried through the metalation and Soxhlet procedures. Similar solubility issues as (phen_2_N_2_)FeCl above trap trace impurities that are not easily removed by washing.

### Synthesis of [(OEP)Fe]_2_O

Octaethylporphinatoiron(III) chloride dissolved in CH_2_Cl_2_ was vigorously shaken in a separatory funnel with aqueous 2 M NaOH. After separating the layers, the organic layer was washed with water, dried over MgSO_4_ and filtered. After washing the MgSO_4_ with CH_2_Cl_2_, the combined organic layers were concentrated to dryness, resulting in a brown microcrystalline solid which was then dried at 60 °C for several hours to give (*μ*-Oxo)bis[(octaethylporphinato)iron(III)], [(OEP)Fe]_2_O. The Fe-O-Fe unit was identified by infrared spectroscopy with characteristic bands at 870 and 832 cm^−1^. These observed values are in line with those reported in the literature for [(OEP)Fe]_2_O^55^. ^57^Fe Mössbauer (90 K): δ = 0.41 mm s^−1^, |Δ*E*_Q_| = 0.67 mm s^−1^.

### Synthesis of [(Pc)Fe]_2_O

0.502 g (0.884 mmol) iron(II) phthalocyanine was suspended in 150 mL THF and stirred open to air for 24 h. The solid is isolated by filtration and is washed with MeOH. After drying the solid in an oven at 60 °C, 0.457 g (90%) of (*μ*-Oxo)bis[(phthalocyaninato)iron(III)], [(Pc)Fe]_2_O, is obtained as a dark blue powder^58,59^. ^57^Fe Mössbauer (90 K): δ = 0.24 mm s^−1^, |Δ*E*_Q_| = 1.26 mm s^−1^.

### Synthesis of (Pc)FeCl

10 g (78.05 mmol, 3.99 equiv.) phthalonitrile and 3.173 g (19.56 mmol) FeCl_3_ were mixed with 20 mL chloronaphthalene. The mixture was brought to reflux and stirred for 18 h. Afterwards, the solid was isolated by filtration and washed with acetone. The solid was dried in an oven at 60 °C to yield 10.891 g (92%) of iron(III) phthalocyanine chloride, (Pc)FeCl, as a dark green powder^107^. ^57^Fe Mössbauer (90 K): δ = 0.28 mm s^−1^, |Δ*E*_Q_| = 2.95 mm s^−1^.

### Zero-field ^57^Fe Mössbauer spectroscopy

Zero-field ^57^Fe Mössbauer spectra were measured with a constant acceleration spectrometer (SEE Co., Minneapolis, MN) at 90 K. Solid samples (20–30 mg for molecular samples and 120 mg for Fe-N-C) were prepared by mixing each sample powder with Paratone-N oil. With the exception of the (phen_2_N_2_)FeCl sample, each sample was prepared outside the glovebox. The (phen_2_N_2_)FeCl sample was prepared similarly except the sample preparation process was carried out inside a nitrogen-filled glovebox and the sample was frozen with liquid nitrogen before handling outside the glovebox. All isomer shifts are reported relative to *α*-Fe metal at 298 K. All data were processed, fitted, and analyzed using an in-house software package for IGOR Pro 6 (Wavemetrics, Lake Oswego, OR).

### X-ray photoelectron spectroscopy

XPS experiments were performed on ThermoFisher Aluminum K Alpha+ (ESCA) or ThermoFisher Nexsa X-ray spectrometer systems with monochromatic aluminum K_α_ X-ray sources (1486.68 eV). Samples for analysis were prepared by distributing a small amount of Au powder (for referencing) on conductive carbon tape and spreading the analyte on top. The analyte was mixed with the Au powder to allow for simultaneous detection of analyte and the Au powder. All experiments were run with the flood gun on to prevent sample charging. All data were collected using a 400 μm, 72 W focused X-ray beam at a base pressure of 2  × 10^−7^ millibar or lower. Survey scans were collected at a pass energy of 200 eV and step size of 1 eV. High-resolution scans were collected with a pass energy of 50 eV and a step size of 0.1 eV. All data were analyzed with the Thermo Avantage software package (v5.987). The Au 4f_7/2_ peak arising from the Au powder was assigned an energy of 84.0 eV and used as an internal binding energy reference for all spectra. In all cases the recorded spectral data were not modified with a smoothing algorithm. High-resolution spectra were fit by application of a Shirley-type (Smart) background and Gaussian/Lorentzian line-shapes of 30% Gaussian shape. The Simplex fitting algorithm was used in all cases.

XPS ratios were taken from survey spectra. We expect these values to be reliable because survey spectra were collected with a high pass energy (200 eV), relatively high step size (1 eV) and because the spectra display a high signal to noise ratio. The [(phen_2_N_2_)Fe]_2_O sample shows a substantial amount of residual chloride relative to iron. This is attributed to variation caused by the insoluble nature of (phen_2_N_2_)Fe complexes which allows small amounts of chloride salts to be carried through the metalation and Soxhlet procedures. A possible assignment in this case is a small amount of triethylammonium chloride, [Et_3_NH]Cl. For the O 1s XPS, the carbon tape gives rise to a set of background oxygen peaks including aromatic C = O (~531 eV, green), aliphatic C–O (~532 eV, blue) and aromatic C–O (~533 eV, yellow)^108^ that dominates the O 1s manifold. This background results from gaps in the microcrystalline molecular film that exposes the background carbon support.

### X-ray absorption spectroscopy

XAS experiments were performed at the 10-BM beamline at the Advanced Photon Source (APS) at Argonne National Laboratory. All measurements were performed at the Fe K edge (7.112 keV) in transmission mode in fast scan from 250 eV below the edge to 550 eV above the edge. Samples were pressed into a stainless-steel sample holder. For catalysts containing 1 wt% Fe, ~12 mg was loaded. For catalysts containing 8–10 wt% Fe, ~1 mg diluted in boron nitride to reach a total of ~10 mg was loaded. The sample holder was placed in a leak-tight sample cell with gas flow capabilities and purged with He at room temperature. The cell was sealed and transferred to the beamline to be scanned.

The data were interpreted using WinXAS 3.1 software to find the coordination number (CN) and bond distance (R) using standard procedures. The phase and amplitude functions for Fe–N and Fe–O were extracted from theoretical Feff6 calculations. Theoretical phase and amplitude files were created for the Fe–N (CN = 1, *R* = 1.94 Å) and Fe–O (CN = 1, *R* = 1.99 Å) scattering pairs obtained from known reference compounds. Least squared fits of the first shell of r-space and isolated q-space were performed on the *k*^2^-weighted Fourier transform data over the range 2.71–10 Å^−1^ in each spectrum to fit the magnitude and imaginary components. The fits were calibrated using the Fe foil (CN = 8, *R* = 2.54 Å). During the fit of the Fe foil, the CN was fixed to 8 and an amplitude reduction factor (S_o_^2^) of 0.64 was obtained. The S_o_^2^ was fixed as 0.64 to fit all other EXAFS data. WinXAS software provides error calculations for each parameter, generated from a correlation matrix during the least squared fit. These values are on the order of +/−10% for the CN, +/−5% for R, and +/−0.0001 for the Debye-Waller factor (s^2^). The s^2^ stands for the mean square variation in the bond distances.

### Computational details

All DFT calculations were performed using the ab-initio software package Q-Chem^109^, the meta-hybrid functional TPSSh^110^ and the basis set 6–31+G*^111–113^. To model solvation, the implicit solvation model IEF-PCM with a dielectric constant of 78.4 was used^114^. All molecular images were generated using the software package VESTA^115^. The geometries of (phen_2_N_2_)Fe(III)Cl and [(phen_2_N_2_)Fe(III)]^+^ were optimized with spin states *S* = 1/2, 3/2, and 5/2. The most stable spin state was found to be *S* = 3/2 in both cases.

### General electrochemical methods

Sodium hydroxide (99.99%, semiconductor grade), potassium ferricyanide (ACS grade) and potassium sulfate (99.99%, semiconductor grade) were obtained from Sigma Aldrich and used as received. 5 wt% perfluorinated Nafion® resin solution was obtained from Ion Power Inc. and used as received. Aqueous electrolyte solutions were prepared with deionized water purified to a resistivity of 18.2 M Ω-cm using a Milliq water purification system (Millipore Corporation). Vulcan VXC72R carbon powders were obtained from Cabot Corporation and used as received. Glassy carbon disk electrodes were obtained from Pine Research Instrumentation, Inc. Hg/HgO and Hg/Hg_2_SO_4_ reference electrodes were obtained from CH Instruments, Inc. A titanium mesh was used as the counter electrode for all electrochemical experiments. The titanium counter was constructed of titanium wire (99.9%) and titanium gauze (40 mesh) obtained from Alfa Aesar and was treated with aqua regia prior to use.

All electrochemical experiments were conducted at ambient temperature using a Biologic VSP 16-channel potentiostat and a three-electrode electrochemical cell. In all cases, the Ti counter electrode was isolated from the working compartment by a porous glass frit. Hg/HgO and Hg/Hg_2_SO_4_ reference electrodes were used for all experiments conducted in alkaline and acidic aqueous electrolytes, respectively. The Hg/HgO reference was stored in 3 M NaOH solution between measurements. Electrode potentials measured in aqueous alkaline media were converted to the RHE scale using the following relationship: *E*(RHE) = *E*(Hg/HgO) + 0.094 V + 0.059*(pH) V. The Hg/Hg_2_SO_4_ electrode was stored in saturated K_2_SO_4_ solution between measurements. Electrode potentials measured in aqueous acidic media were converted to the RHE scale using the relationship: *E*(RHE) = *E*(Hg/Hg_2_SO_4_) + 0.65 V + 0.059*(pH) V. Reference electrodes were periodically checked against a pristine reference to ensure against potential drift.

Routine electrochemical measurements were performed with a 5 mm diameter glassy carbon disk working electrode rotated at 2000 rpm. All scan rates were 5 mV s^−1^ to allow for equilibration and to approximate steady state catalytic operation. Cyclic and linear sweep voltammograms were initiated at the open circuit potential. In all cases, the potential was swept in the negative direction. Solution resistance values were <10 Ω and were ignored. All voltammograms were recorded without *i*R compensation. Solutions were sparged for 10 min before each experiment with either N_2_ or O_2_ and for another 10 min upon changing between gases. During an experiment, the headspace of the cell was continually flushed with the working gas to minimize solution contamination by atmospheric gases. All experiments were conducted using 10 μL of catalyst ink dropcast onto a polished glassy carbon RDE. Catalytic onset potentials were taken to be the potential at which the observed current density reached –0.1 mA cm^−^^2^ . Buffers used for the pH-dependence study of the (phen_2_N_2_)FeCl ink consisted of a modified Britton-Robinson buffer (50 mM each of NaHSO_4_, Na_2_HPO_4_ and B(OH)_3_) that was adjusted to the desired pH value with concentrated HClO_4_ or NaOH.

### Preparation of Fe-N-C ink

Fe-N-C inks for use in electrochemical studies were prepared by sequentially adding CH_2_Cl_2_, EtOH, and 5 wt% Nafion solution to 11 mg Fe-N-C catalyst in a ratio of 7:2:1 to give a final volume of 1 mL and an Fe-N-C concentration of 11 mg mL^−1^. The resulting mixture was sonicated for 16 min to give the product ink. Inks were produced in 1 mL batches. Superior results in terms of carbon powder dispersion and ORR activity were obtained when CH_2_Cl_2_ was added to the precursor powder first, followed by EtOH and Nafion solution. The Fe-N-C data presented in Fig. 5 and Supplementary Fig. 25 was collected at an Fe-N-C concentration of 4.7 mg mL^−1^ in order to limit the double-layer capacitance, which can obscure redox peaks. See Supplementary Table 10 for nominal dropcast film mass loadings.

### Preparation of (phen_2_N_2_Fe)Cl, (Pc)FeCl and (OEP)FeCl Inks

Inks for use in electrochemical studies were prepared by suspending ~1.1 mg mL^−1^ catalyst (either porphyrin- or (phen_2_N_2_)-based) in ~2.5 mg mL^−1^ Vulcan VXC72R carbon. CH_2_Cl_2_, EtOH and 5 wt% Nafion solution are added sequentially in a ratio of 7:2:1. The resulting mixtures were sonicated for 16 min to give the product inks. The inks were produced in 1 mL batches in all cases. Note that superior results in terms of carbon powder dispersion and ORR activity were obtained when CH_2_Cl_2_ was added to the solid precursor mixture first followed by EtOH and Nafion solution. See Supplementary Table 10 for nominal dropcast film mass loadings.

### Rotating ring disk electrode voltammetry

RRDE voltammetry was performed with a Pine rotator using a ChangeDisk tip. RRDE experiments conducted in acidic media were performed with a platinum ring while experiments conducted in alkaline media were performed with a gold ring^116^. The Pt and Au rings were cleaned by cyclic voltammetry conducted at 50 mV s^−1^ over potential ranges of 1 to −0.8 V and 1.1 to −0.7 V, respectively, vs the Hg/Hg_2_SO_4_ reference in 0.1 M HClO_4_. The potential was swept over the appropriate range until the cyclic voltammograms overlaid exactly.

The proportion of H_2_O_2_ produced during ORR was quantified using the following relationship^95^:

3 $$\% {\mathrm{H}}_{2}{\mathrm{O}}_{2} = \frac{{100\left( {\frac{{2i_{{\mathrm{ring}}}}}{N}} \right)}}{{i_{{\mathrm{disk}}} + \left( {\frac{{i_{{\mathrm{ring}}}}}{N}} \right)}}$$

In this case, *i*_ring_ and *i*_disk_ are the currents at the ring and disk electrodes, respectively and *N* is the collection efficiency. The number of electrons transferred as a function of applied potential was calculated using the following relationship^117^:

4 $$n_{{\mathrm{e}}-} = \frac{{4i_{{\mathrm{disk}}}}}{{i_{{\mathrm{disk}}} + \left( {\frac{{i_{{\mathrm{ring}}}}}{N}} \right)}}$$

The collection efficiency constant, *N*, was calculated at the end of each group of experiments under a given set of conditions by examining the Fe(III/II) couple via chronoamperometry. The ring was polarized at 1.5 V vs RHE while the disk was held at 0.1 V vs RHE and currents were monitored for 60 seconds. The current values over the last 10 s were averaged and taken as the values for *i*_ring_ and *i*_disk_. A 60 s chronoamperogram was also run with the ring as the working electrode to give the background oxidative current at 1.5 V vs RHE. Averaging the last 10 s of the background trace gave the value of *i*_ring0_. Collection efficiency measurements were conducted using a blank glassy carbon disk. Fe_2_(SO_4_)_3_ and K_3_Fe(CN)_6_ were used as the redox agents for the collection efficiency measurements in acid and base, respectively. The collection efficiency was calculated using Eq. (5) and found to be 10–11% at the Pt ring and 22–23% at the Au ring^116^:

5 $$N = \frac{{i_{{\mathrm{ring}}}-i_{{\mathrm{ring}}0}}}{{i_{{\mathrm{disk}}}}}$$

Since calculated %H_2_O_2_ values are unreliable when disk currents are small (near the ORR onset region), each %H_2_O_2_ trace is shown starting where dependable data are available and noise in the measurement has been minimized. Although each catalyst is distinct, this point was located between 50–100 mV past the ORR onset potential in all cases.

## Supplementary information

Supplementary Information

## Data Availability

Data generated or analysed during this study are included in this article and/or its supplementary information files. Raw data are available from the authors on request.
