# Supplementary material for: A pyridinic Fe-N4 macrocycle models the active sites in Fe/N-doped carbon electrocatalysts
Source: Nat Commun. 2020 Oct 19;11:5283. doi: 10.1038/s41467-020-18969-6 (PMC7572418; doi:10.1038/s41467-020-18969-6)
Supplement: Supplementary file 1 — Supplementary Information [file 41467_2020_18969_MOESM1_ESM.pdf]

Supplementary Information

A Pyridinic Fe-N_4 Macrocycle Models the Active Sites in Fe/N-Doped Carbon Electrocatalysts

Marshall-Roth *et al.*

Supplementary Figures

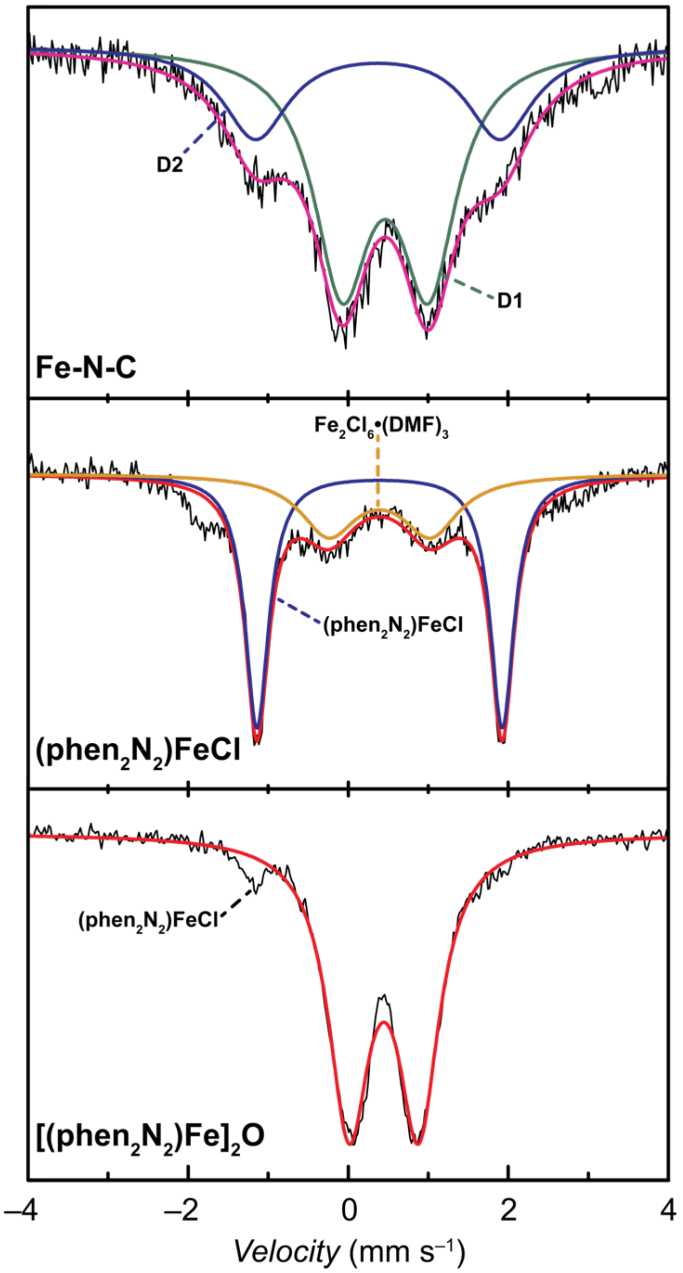

Supplementary Figure 1. Zero-field ^{57}Fe Mössbauer spectra of Fe-N-C and (phen$_2$N$_2$) complexes. Fe-N-C: [D1: (δ = 0.46 mm s^{-1}, $|\Delta E_Q|$ = 1.08 mm s^{-1}) 71%, D2: (δ = 0.37 mm s^{-1}, $|\Delta E_Q|$ = 3.06 mm s^{-1}) 29%, top], (phen$_2$N$_2$)FeCl: [(δ = 0.39 mm s^{-1}, $|\Delta E_Q|$ = 3.06 mm s^{-1}), middle] and [(phen$_2$N$_2$)Fe]$_2$O: [(δ = 0.45 mm s^{-1}, $|\Delta E_Q|$ = 0.87 mm s^{-1}), bottom] recorded at 90 K. The spectrum of (phen$_2$N$_2$)FeCl was fit to two quadrupole doublets assigned to (phen$_2$N$_2$)FeCl (blue) and a residual Fe$_2$Cl$_6$•(DMF)$_3$ impurity [(δ = 0.39 mm s^{-1}, $|\Delta E_Q|$ = 1.27 mm s^{-1}), yellow]. The D1 and D2 features have been postulated to arise from Fe-N$_4$ sites in high-spin Fe(III) and low-spin Fe(II) states, respectively,[1] although other assignments have been made previously, including D1 as a low spin Fe(II) site and D2 as an intermediate spin Fe(II) site.[2–4] Although the ^{57}Fe Mössbauer parameters for (phen$_2$N$_2$)FeCl are similar to those of the D2 doublet in Fe-N-C, we caution against overinterpreting this similarity because ^{57}Fe Mössbauer spectra are highly sensitive to axial ligation (see below) and the axial Cl in (phen$_2$N$_2$)FeCl is distinct from the putative OH$_x$ ligand present in Fe-N-C materials.

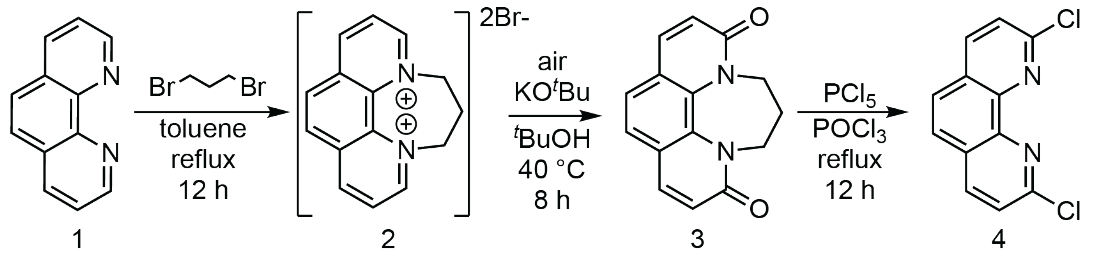

Supplementary Figure 2. Synthesis of 2,9-dichloro-1,10-phenanthroline (4) from 1,10-phenanthroline (1). 2,9-dichloro-1,10-phenanthroline was synthesized by a three-step process starting from 1,10-phenanthroline (**1**). Alkylation of **1** with 1,3-dibromopropane gave 6,7-dihydro-5*H*-1,4-diazepino[1,2,3,4-*lmn*][1,10]phenanthroline-4,8-diium bromide (**2**). Exposure of **2** to KO^{t}Bu in the presence of air gave 6,7-dihydro-5*H*-1,4-diazepino[1,2,3,4-*lmn*][1,10]phenanthroline-3,9-dione (**3**). Conversion of **3** to 2,9-dichloro-1,10-phenanthroline (**4**) was accomplished with PCl_5 in $POCl_3$. Full procedures for each synthesis are given in the Methods section.

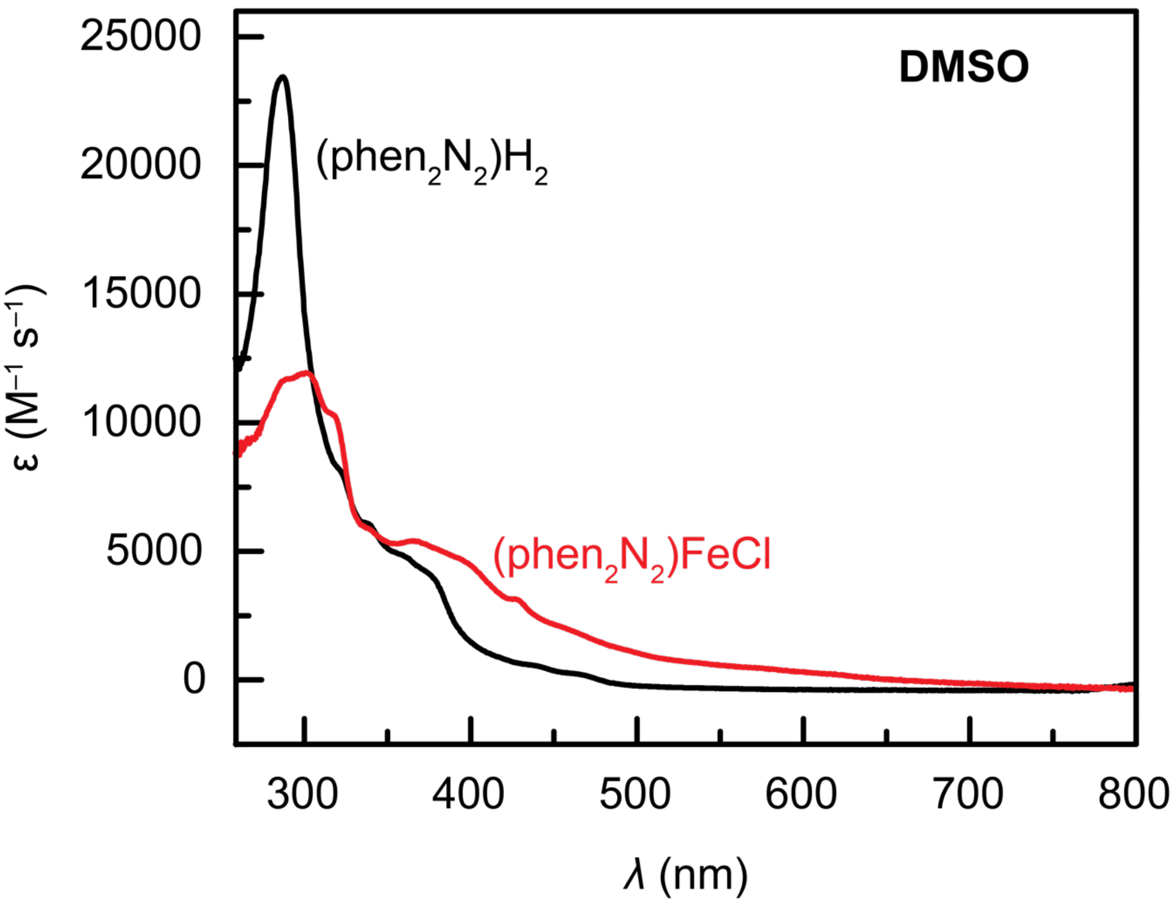

Supplementary Figure 3. Optical spectra of $(phen_2N_2)H_2$ and $(phen_2N_2)FeCl$. $(phen_2N_2)H_2$ (black, 26 μM) and $(phen_2N_2)FeCl$ (red, 15 μM) are shown in DMSO. Upon metalation, the ligand peaks at 360 at 376 nm broaden and increase substantially in intensity relative to the main $\pi \rightarrow \pi^*$ peak upon metalation. A new peak appears at 429 nm, while the broad, low-intensity ligand peaks at 439 and 467 nm vanish. The resulting spectrum is similar to spectra of tetraaza[14]annulene iron(III) chloride complexes in DMSO.[5] No optical transitions were observed beyond 800 nm to the instrumentation limit of 1100 nm.

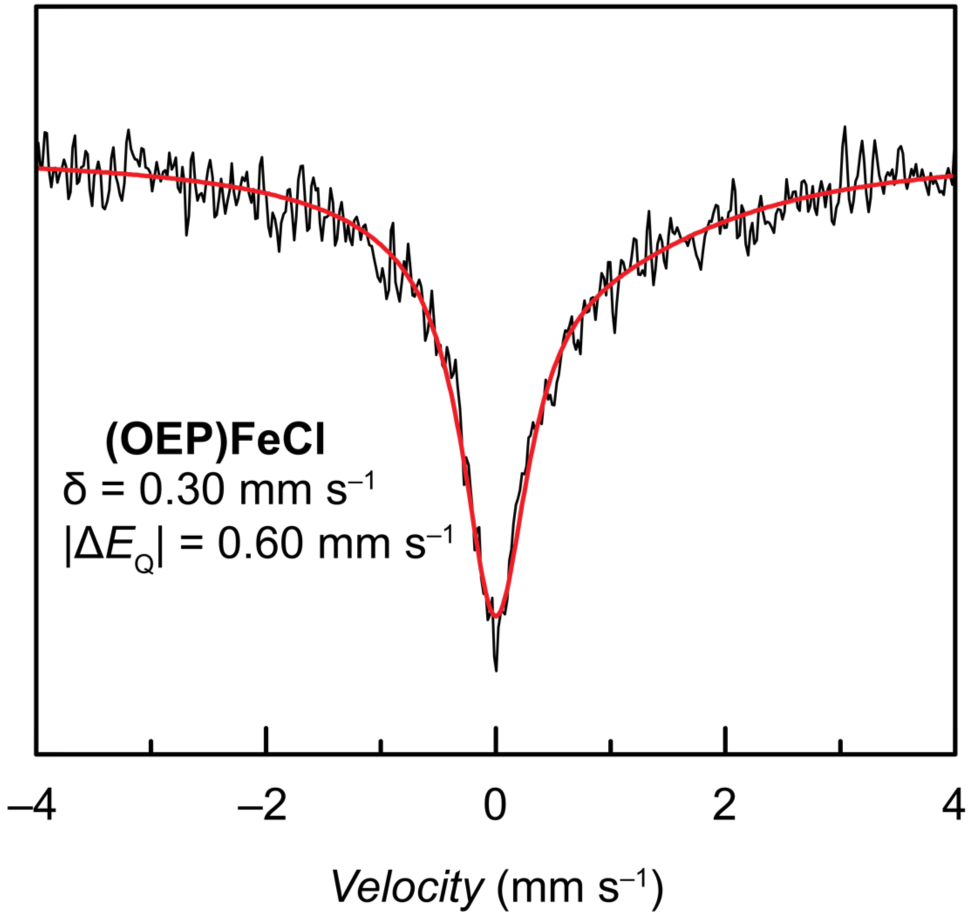

Supplementary Figure 4. Zero-field ^{57}Fe Mössbauer spectrum of (OEP)FeCl. The experimental spectrum recorded at 90 K was fit as an asymmetric doublet (δ = 0.30 mm s^{-1}, $|\Delta E_Q|$ = 0.60 mm s^{-1}, Γ_L=0.69 mm s^{-1} and Γ_R=3.35 mm s^{-1}).

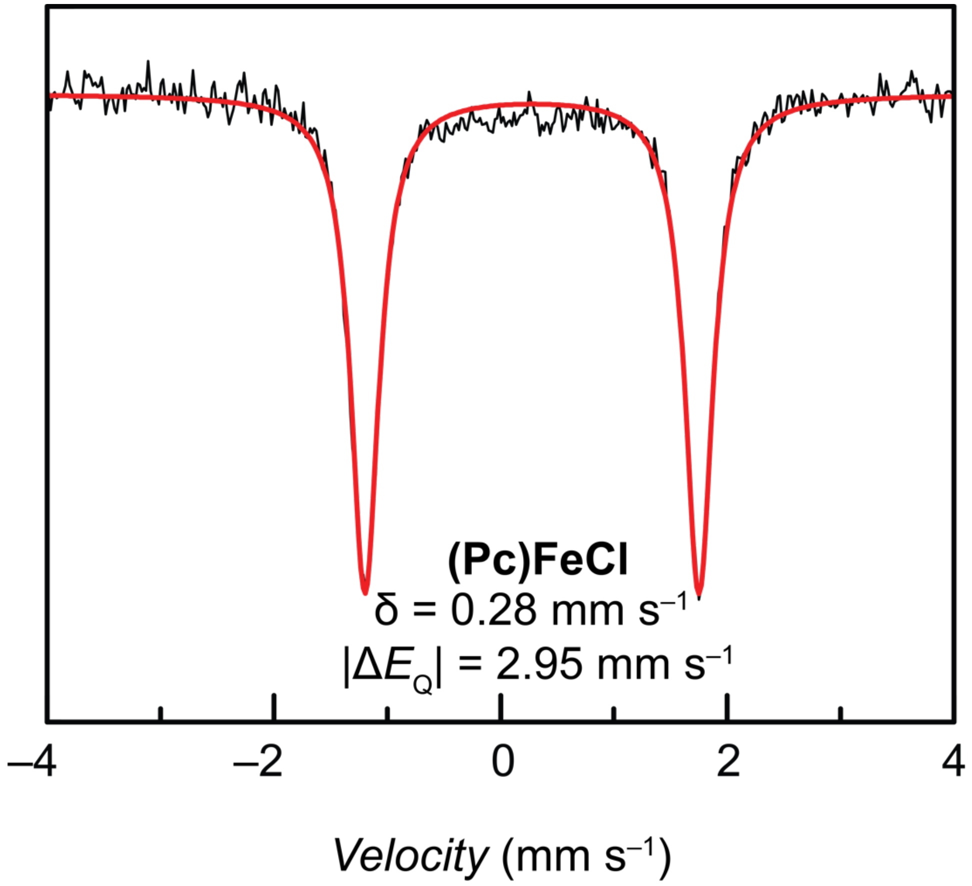

Supplementary Figure 5. Zero-field ^{57}Fe Mössbauer spectrum of (Pc)FeCl. The experimental spectrum at 90 K was fit as a symmetric doublet (δ = 0.28 mm s^{-1}, $|\Delta E_Q|$ = 2.95 mm s^{-1}).

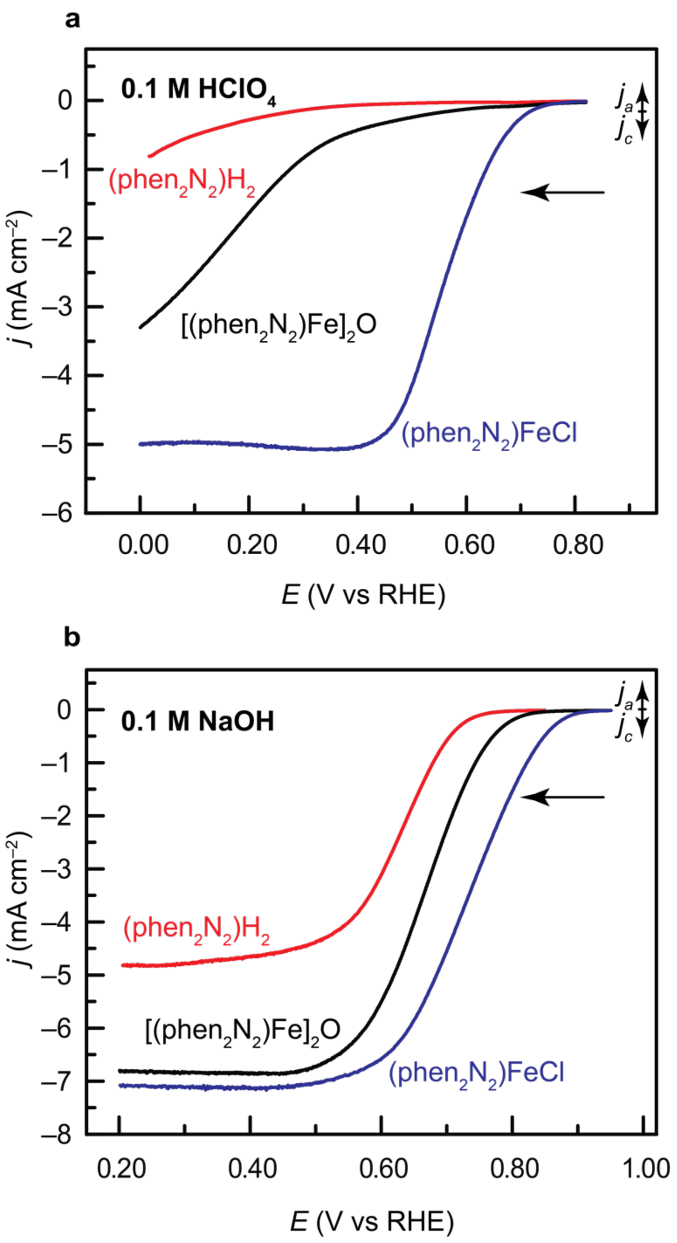

Supplementary Figure 6. ORR linear sweep voltammogram traces of adsorbed (phen$_2$N$_2$) compounds. Scans of (phen$_2$N$_2$)FeCl (blue), (phen$_2$N$_2$)H$_2$ (red) and [(phen$_2$N$_2$)Fe]$_2$O (black) are shown in 0.1 M $HClO_4$ (**a**) and 0.1 M NaOH (**b**). The data were recorded at 2000 rpm rotation rate and a scan rate of 5 mV s^{-1}.

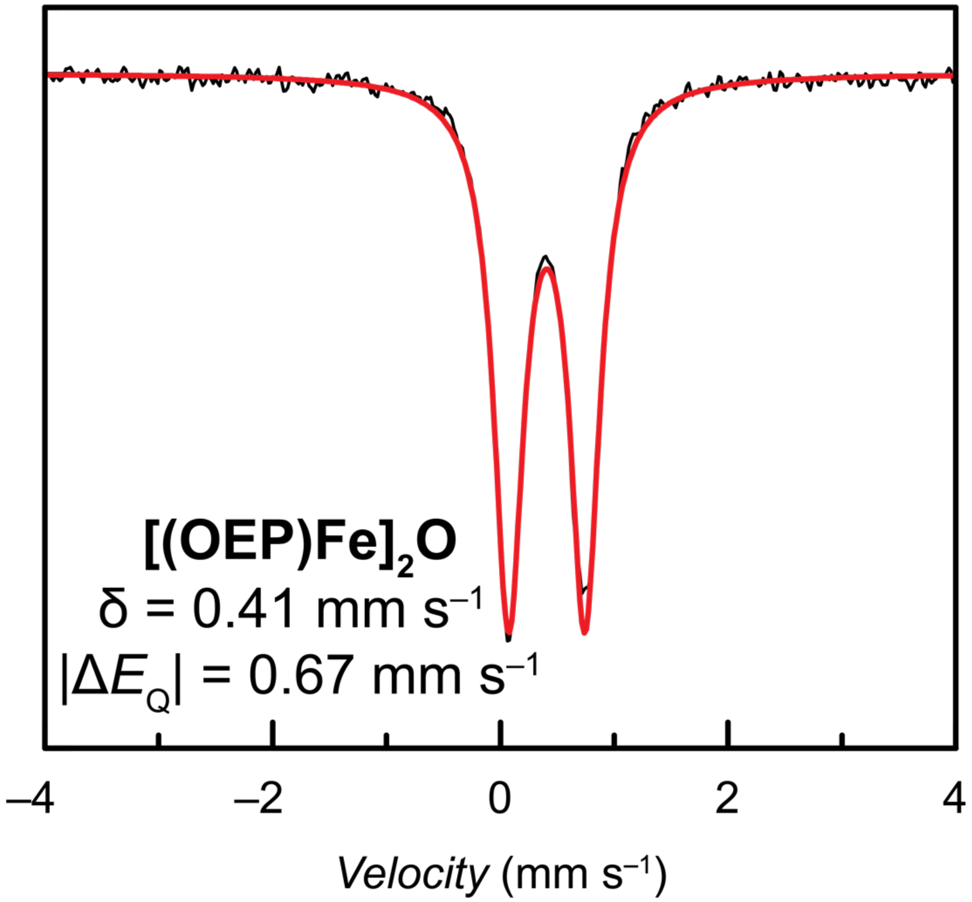

Supplementary Figure 7. Zero-field ^{57}Fe Mössbauer spectrum of [(OEP)Fe]$_2$O. The experimental spectrum was recorded at 90 K and fit as a symmetric doublet (δ = 0.41 mm s^{-1}, $|\Delta E_Q|$ = 0.67 mm s^{-1}).

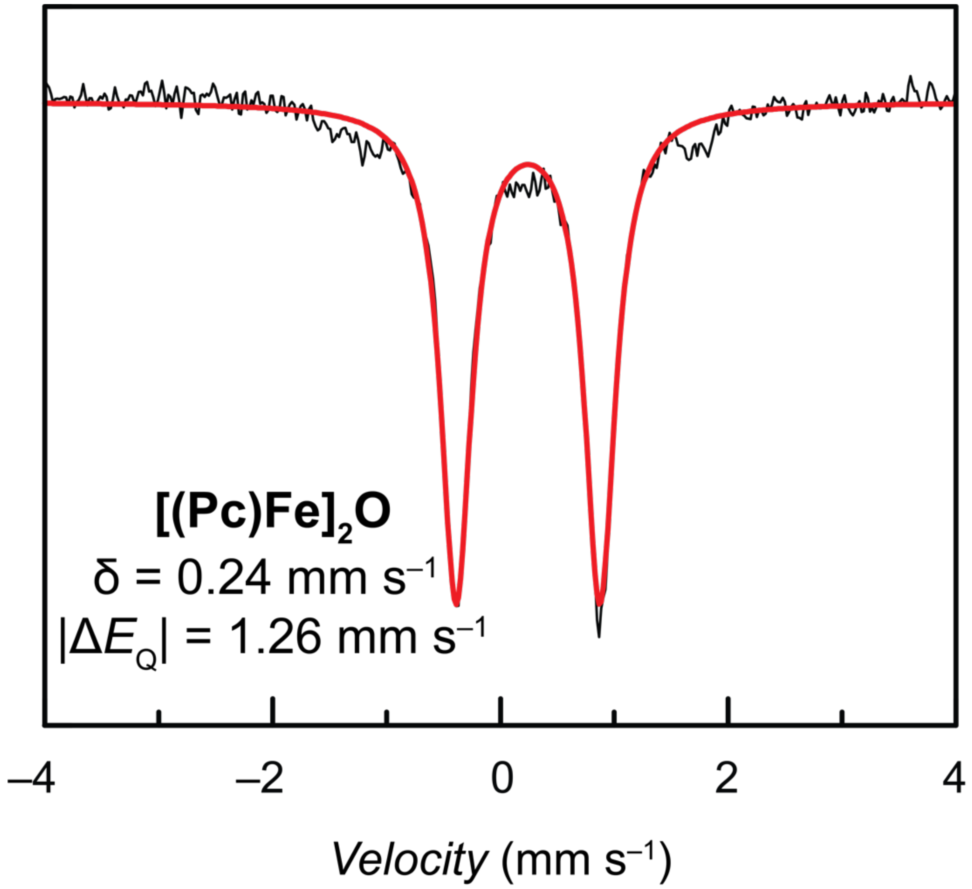

Supplementary Figure 8. Zero-field ^{57}Fe Mössbauer spectrum of $[(Pc)Fe]_2O$. The experimental spectrum was recorded at 90 K and fit as a symmetric doublet ($\delta = 0.24$ mm s^{-1}, $|\Delta E_Q| = 1.26$ mm s^{-1}).

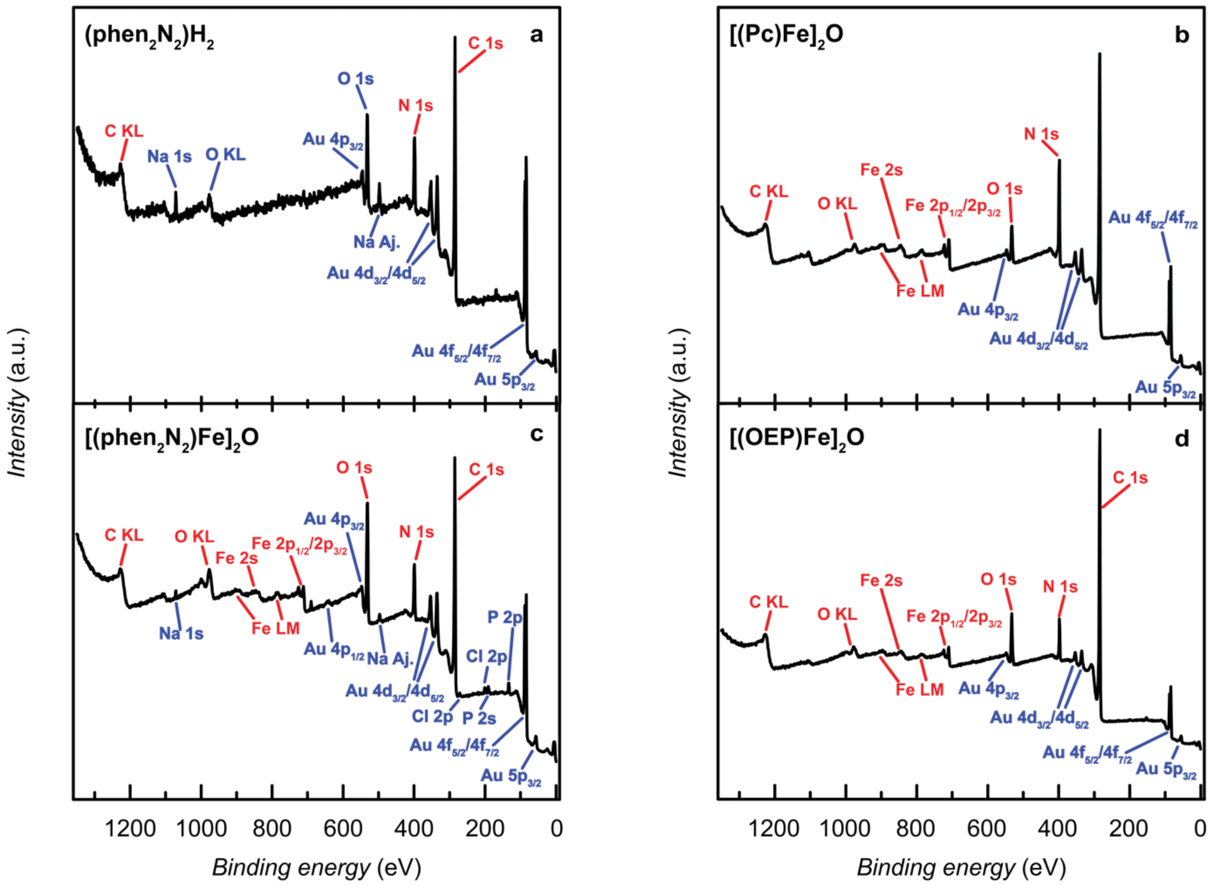

Supplementary Figure 9. XPS survey spectra of $(phen_2N_2)H_2$ and μ-oxo model complexes. $(phen_2N_2)H_2$ (**a**), $[(Pc)Fe]_2O$ (**b**), $[(phen_2N_2)Fe]_2O$ (**c**), and $[(OEP)Fe]_2O$ (**d**) powders were mixed with Au powder and pressed onto carbon tape. Red labels refer to elements present in the molecular formula of the analyte while blue labels denote other elements.

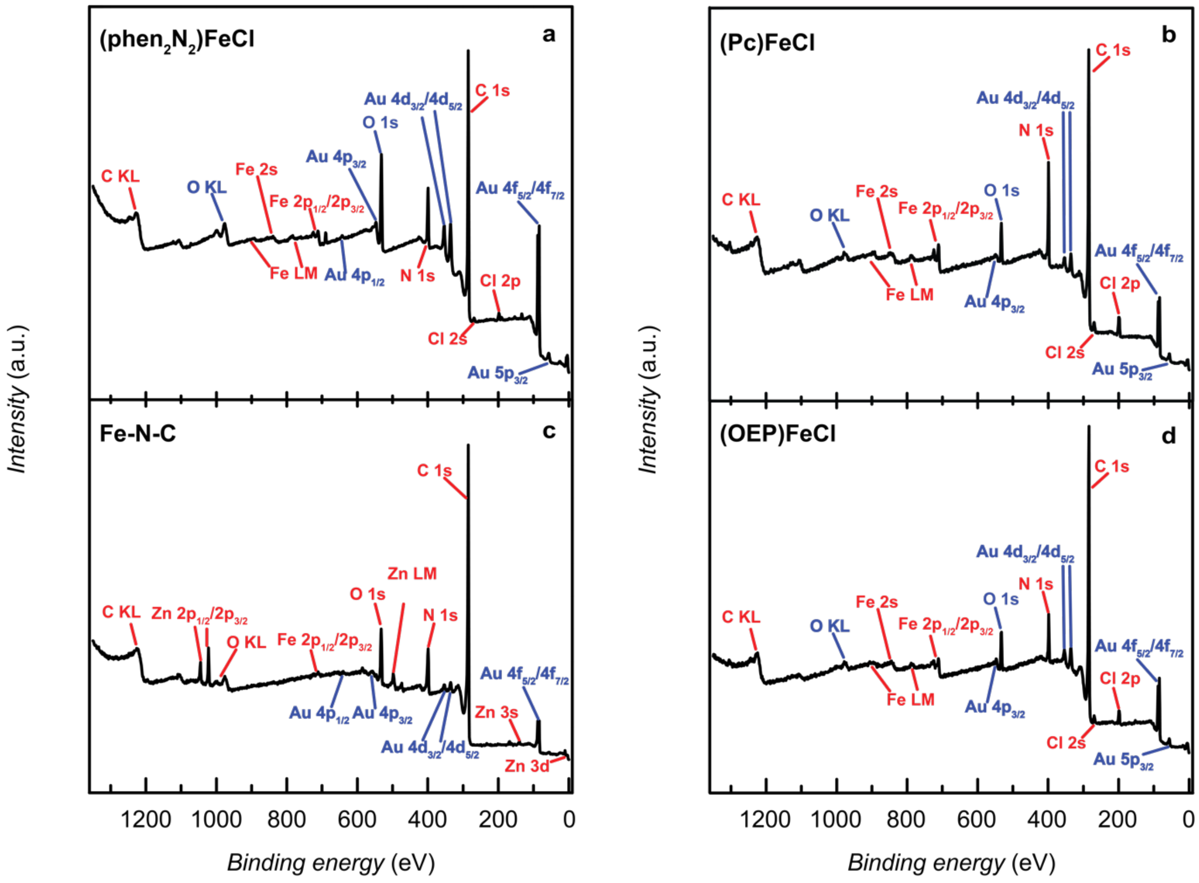

Supplementary Figure 10. XPS survey spectra of Fe-N-C and the ORR catalyst complexes. (phen$_2$N$_2$)FeCl (**a**), (Pc)FeCl (**b**), Fe-N-C (**c**), and (OEP)FeCl (**d**) powders were mixed with Au powder and pressed onto carbon tape. Red labels refer to elements present in the molecular formula of the analyte while blue labels denote other elements.

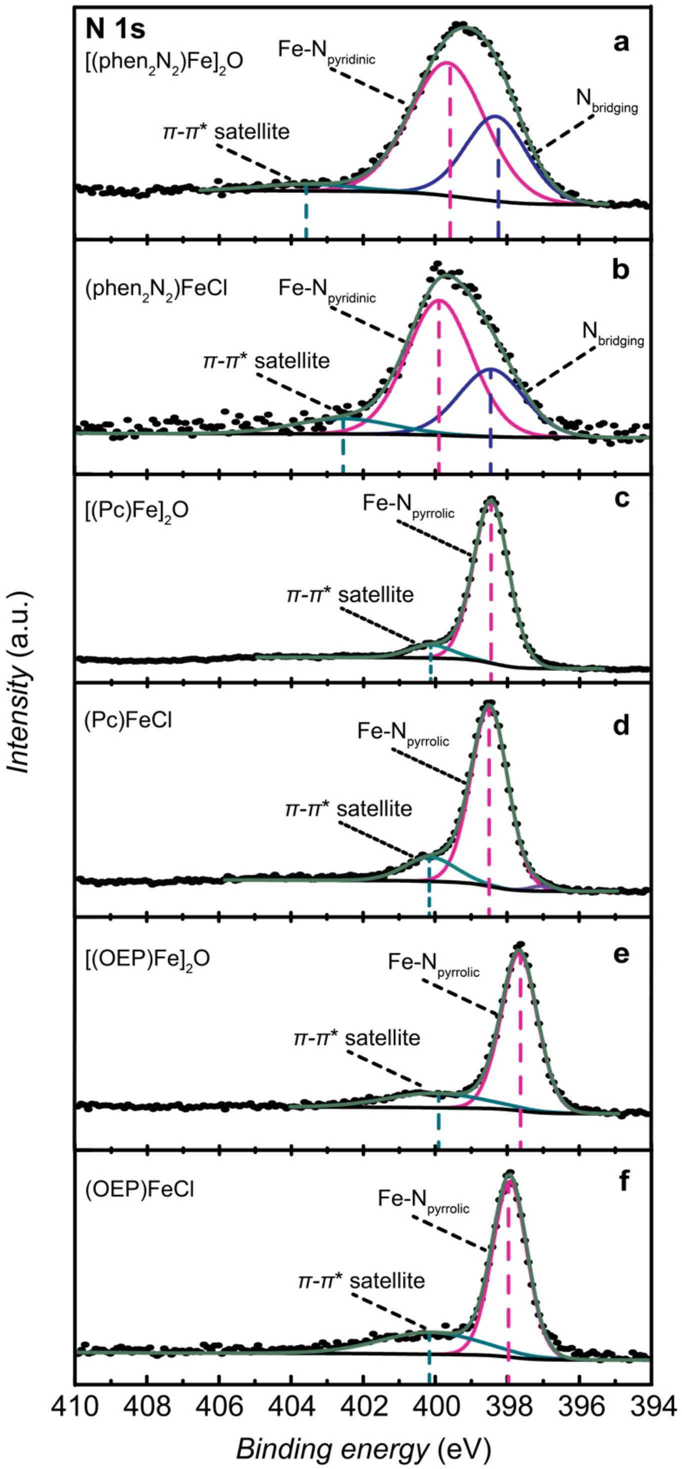

Supplementary Figure 11. XPS high resolution N 1s spectra of the molecular iron complexes. [(phen$_2$N$_2$)Fe]$_2$O (**a**), (phen$_2$N$_2$)FeCl (**b**), [(Pc)Fe]$_2$O (**c**), (Pc)FeCl (**d**), [(OEP)Fe]$_2$O (**e**), and (OEP)FeCl (**f**) were mixed with Au powder and pressed onto conductive carbon tape. The bridging and metal-coordinated N environments are shown as blue and magenta components, respectively, while the π satellites are represented as dark cyan components.

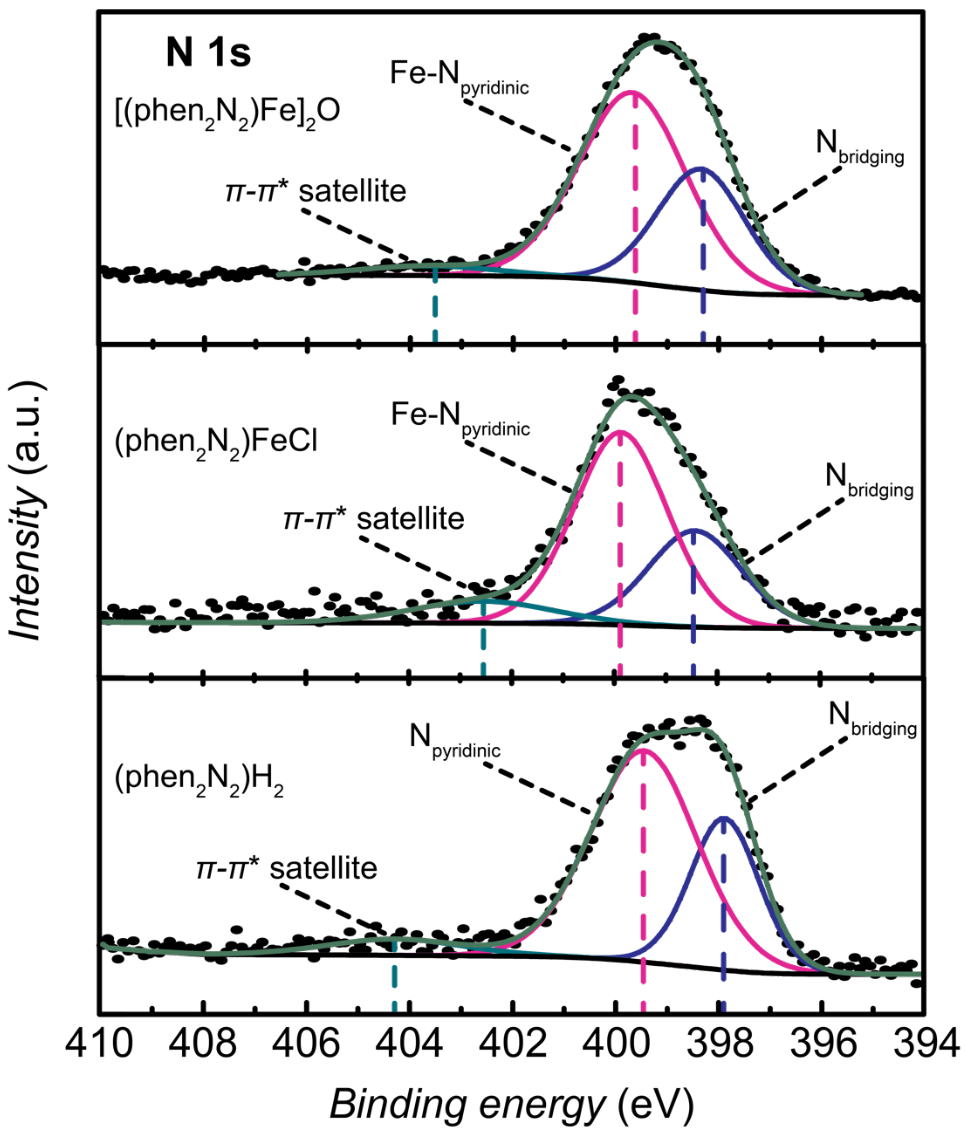

Supplementary Figure 12. XPS high-resolution N 1s spectra of ($phen_2N_2$) compounds. [($phen_2N_2$)Fe]$_2$O (**top**), ($phen_2N_2$)FeCl (**middle**) and ($phen_2N_2$)H_2 (**bottom**) powders were mixed with Au powder and pressed onto conductive carbon tape. The bridging and pyridinic N environments are shown as blue and magenta components, respectively, while the π satellites are represented as dark cyan components.

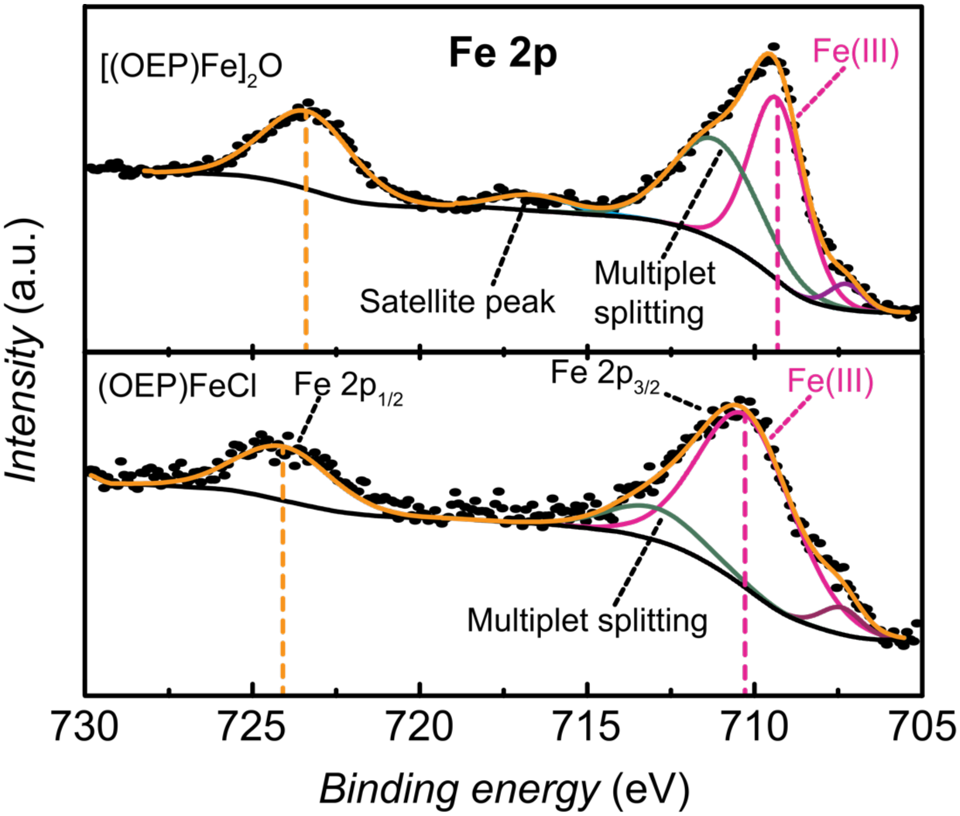

Supplementary Figure 13. XPS high resolution Fe 2p spectra of (OEP)Fe complexes. $[(OEP)Fe]_2O$ (**top**) and (OEP)FeCl (**bottom**) were mixed with Au powder and pressed onto conductive carbon tape. Asymmetry in the Fe $2p_{3/2}$ peak manifold was fit by three components: a pre-peak (purple), a main peak (magenta), and a multiplet peak (green).

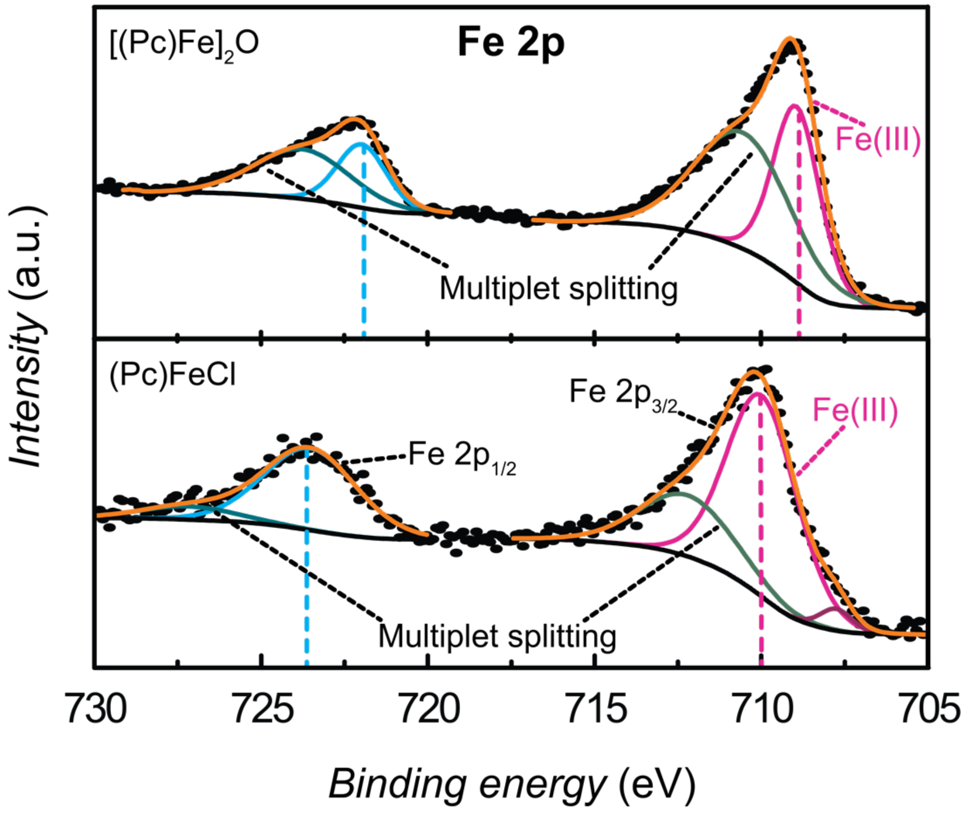

Supplementary Figure 14. XPS high resolution Fe 2p spectra of (Pc)Fe complexes. [(Pc)Fe]$_2$O (**top**) and (Pc)FeCl (**bottom**) were mixed with Au powder and pressed onto conductive carbon tape. Asymmetry in the Fe 2p$_{3/2}$ peak was fit by two or three components: a pre-peak (purple), a main peak (magenta) and a multiplet peak (green).

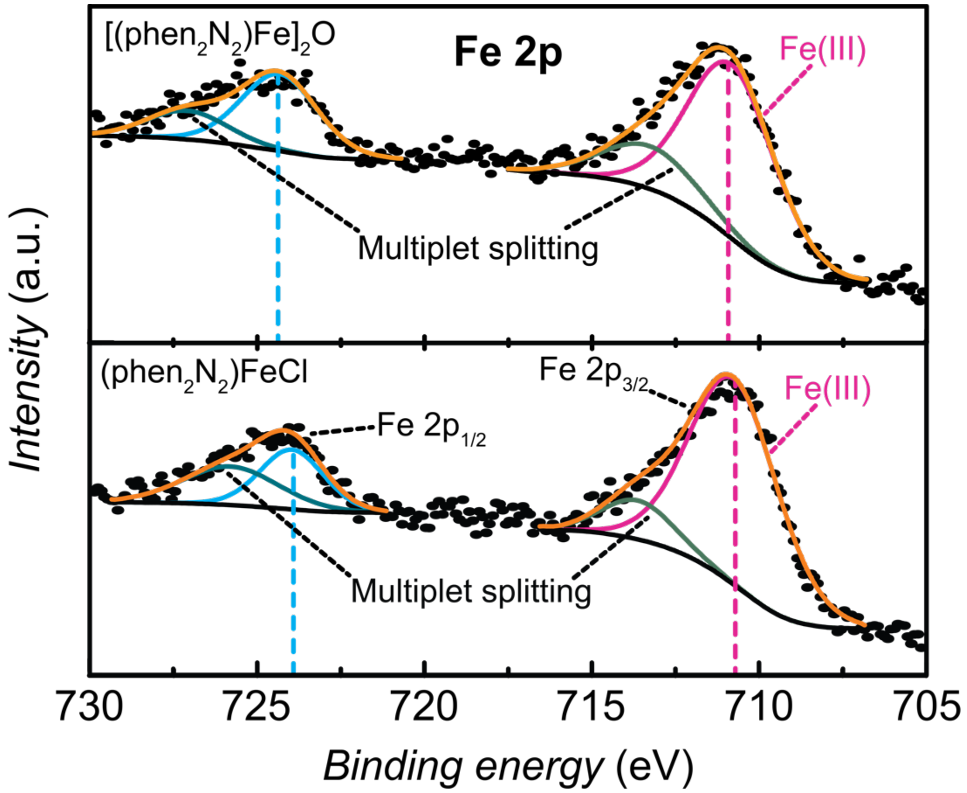

Supplementary Figure 15. XPS high resolution Fe 2p spectra of ($phen_2N_2$)Fe complexes. [($phen_2N_2$)Fe]$_2$O (**top**) and ($phen_2N_2$)FeCl (**bottom**) were mixed with Au powder and pressed onto conductive carbon tape. Asymmetry in the Fe $2p_{3/2}$ peak was fit by two components: a main peak (magenta) and a multiplet peak (green).

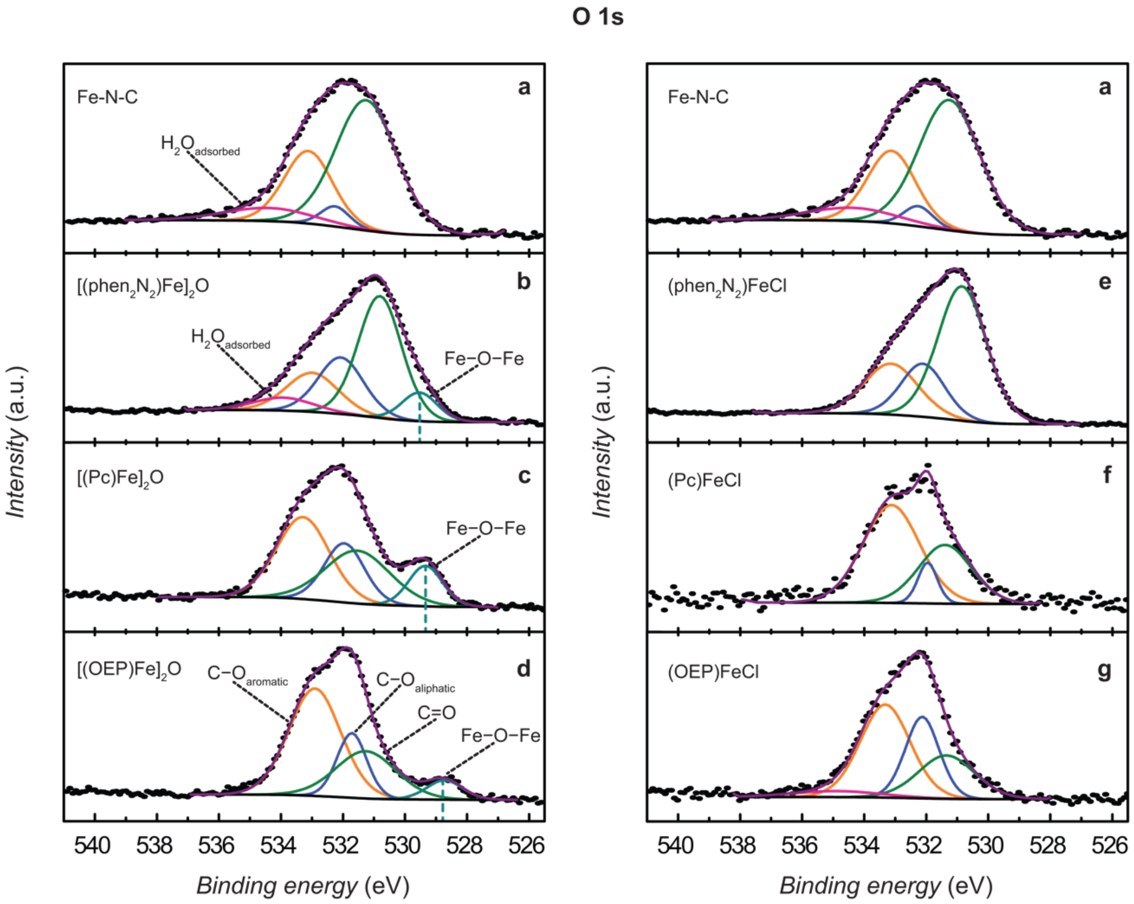

Supplementary Figure 16. XPS high resolution O 1s spectra of all iron containing materials. Fe-N-C (**a**), [(phen$_2$N$_2$)Fe]$_2$O (**b**), [(Pc)Fe]$_2$O (**c**), [(OEP)Fe]$_2$O (**d**), (phen$_2$N$_2$)FeCl (**e**), (Pc)FeCl (**f**), and (OEP)FeCl (**g**) were mixed with Au powder and pressed onto conductive carbon tape.

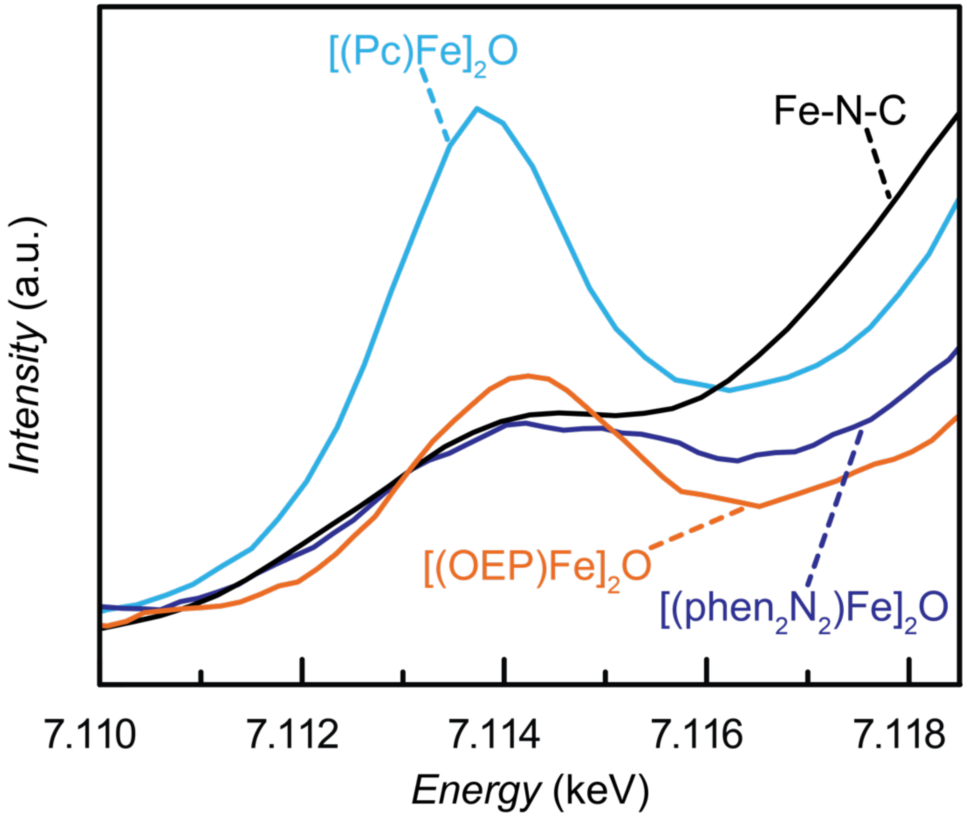

Supplementary Figure 17. Iron K-edge pre-edge features of the model complexes and Fe-N-C. Iron K-edge XANES spectra of $[(phen_2N_2)Fe]_2O$ (blue), Fe-N-C (black), $[(OEP)Fe]_2O$ (orange) and $[(Pc)Fe]_2O$ (aqua) in the region of the pre-edge feature (named feature A in the text).

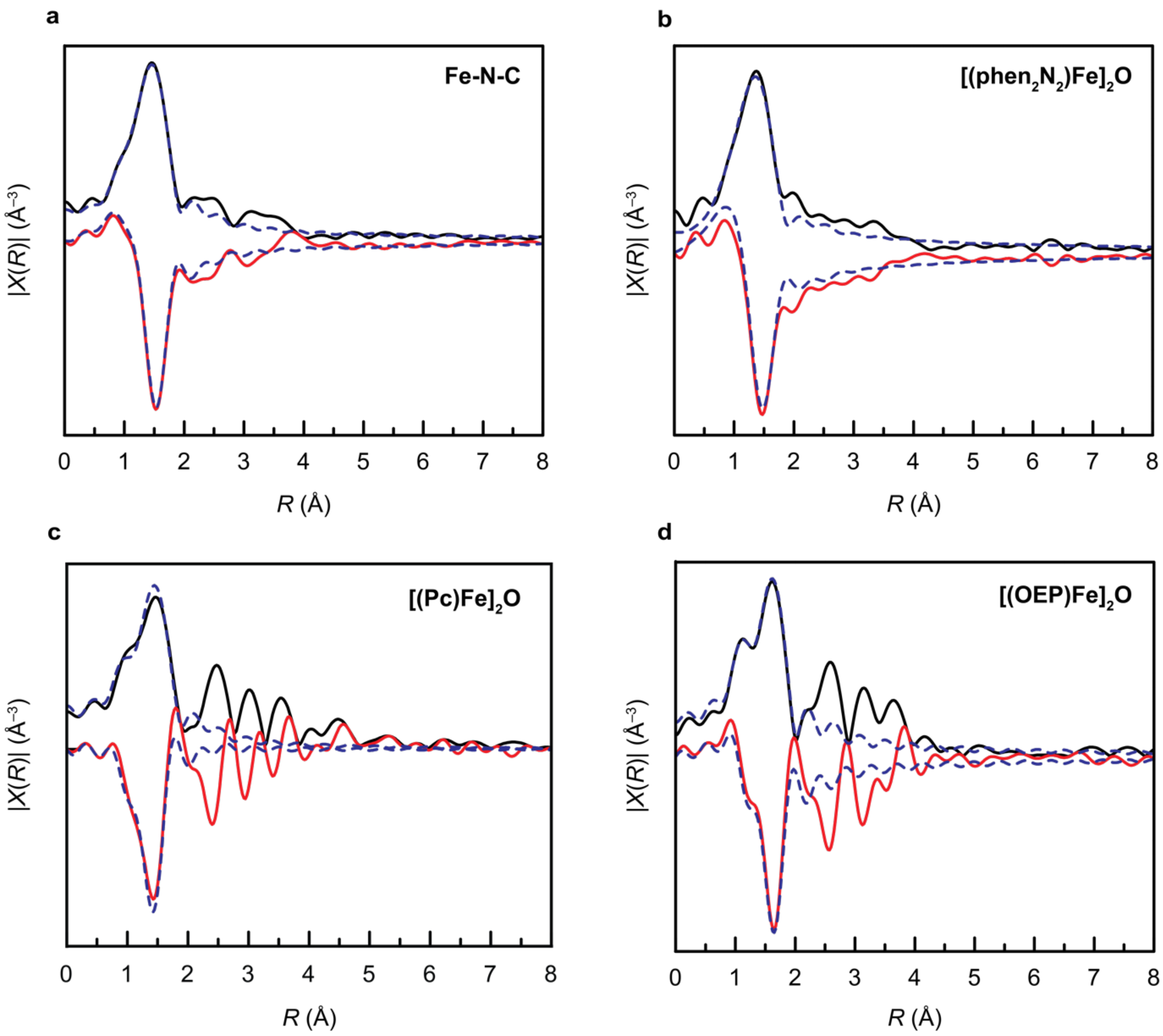

Supplementary Figure 18. Fits of EXAFS data for Fe-N-C and the model iron complexes. Fe-N-C (**a**), [(phen$_2$N$_2$)Fe]$_2$O (**b**), [(Pc)Fe]$_2$O (**c**), and [(OEP)Fe]$_2$O (**d**). Real and imaginary components are shown as black and red traces respectively with fits given as dashed blue traces.

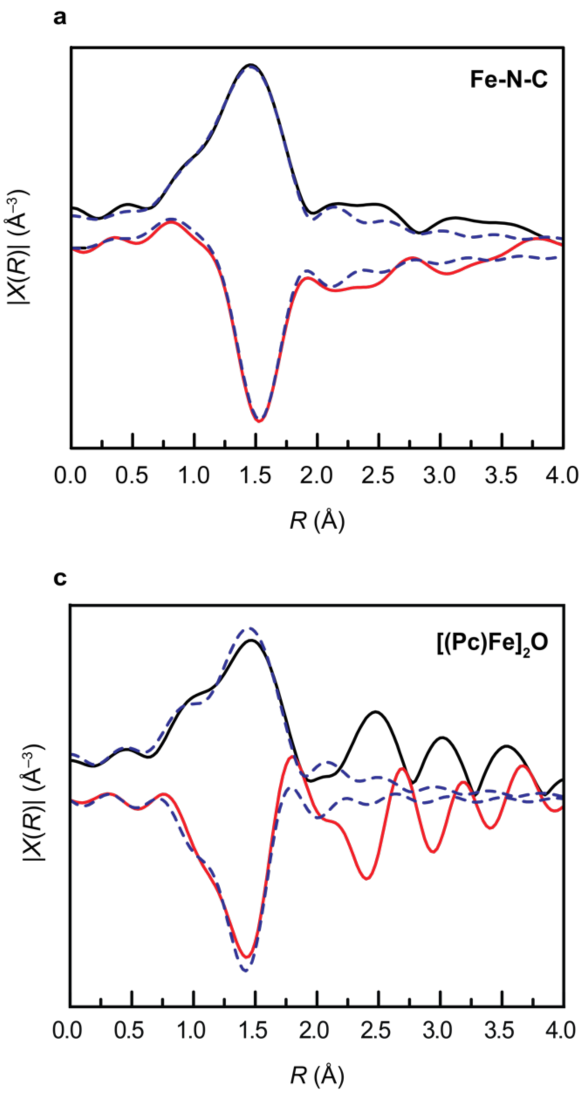

Supplementary Figure 19. Expanded view of fits of EXAFS data for Fe-N-C and the model iron complexes. Fe-N-C (**a**), [(phen$_2$N$_2$)Fe]$_2$O (**b**), [(Pc)Fe]$_2$O (**c**), and [(OEP)Fe]$_2$O (**d**). Real and imaginary components are shown as black and red traces respectively with fits given as dashed blue traces.

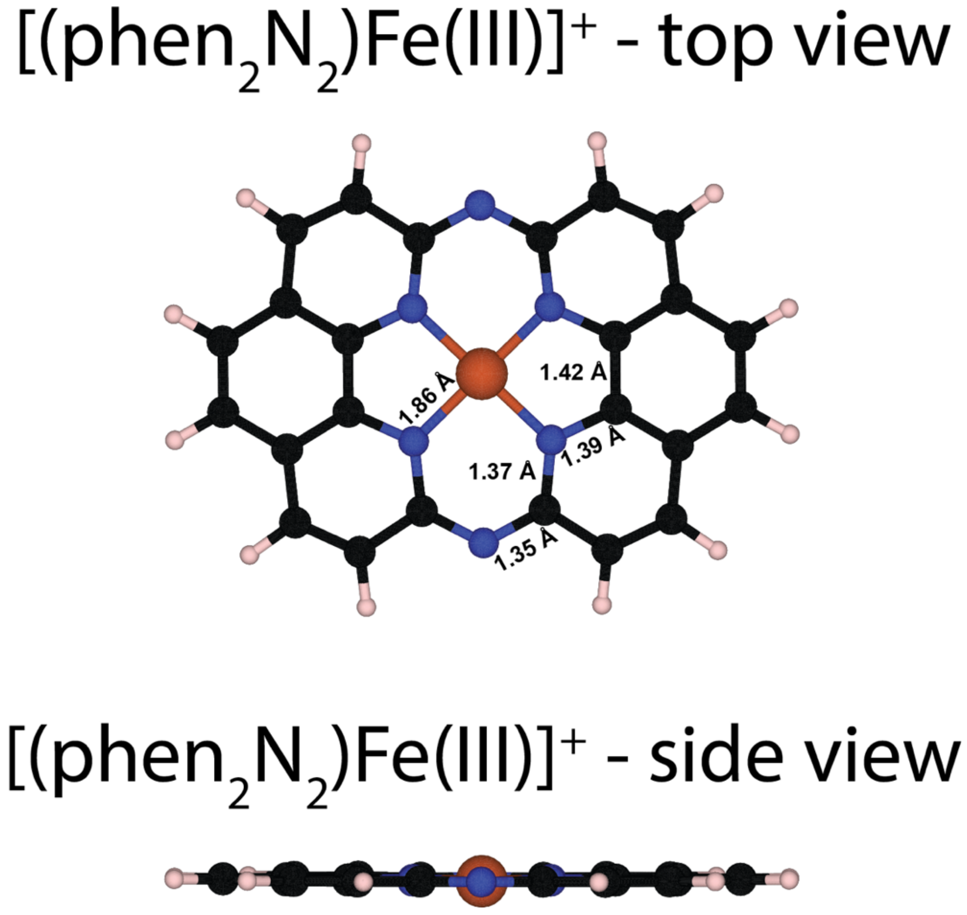

Supplementary Figure 20. Calculated structure of $[(phen_2N_2)Fe(III)]^+$. DFT-computed structure of $[(phen_2N_2)Fe(III)]^+$ (S = 3/2) visualized from the top and side.

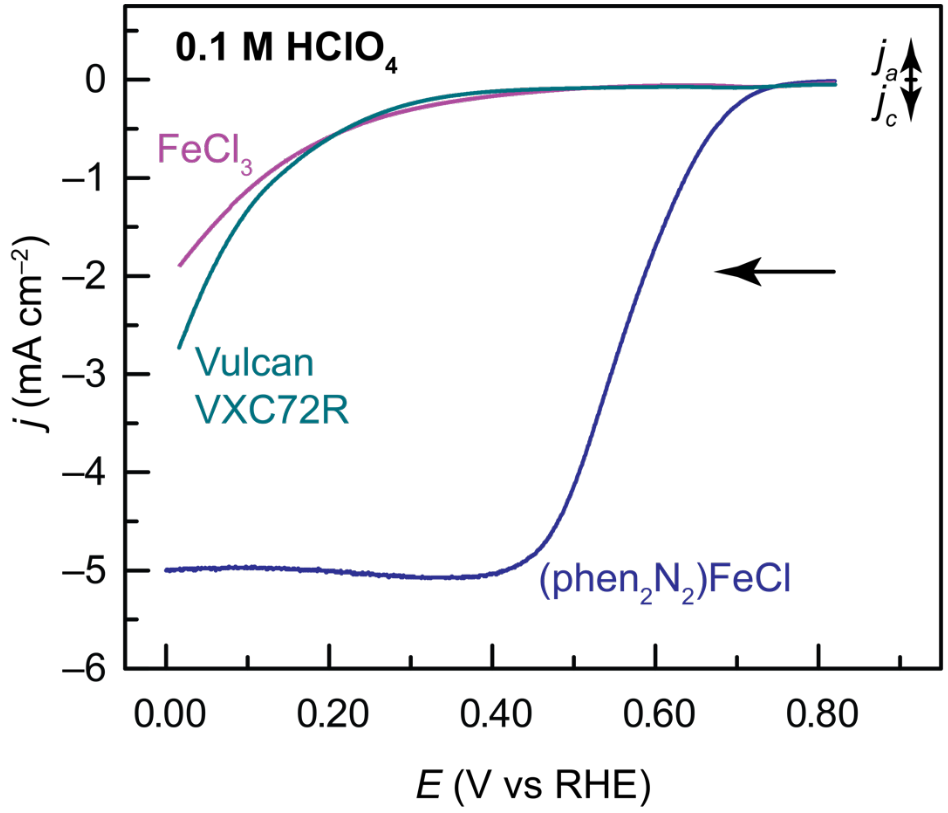

Supplementary Figure 21. ORR performance of ($phen_2N_2$)FeCl compared to background and iron-contaminated carbon. Linear sweep voltammograms of ($phen_2N_2$)FeCl (blue), Vulcan carbon (green) and $FeCl_3$/Vulcan (purple) inks polarized in O_2-saturated 0.1 M $HClO_4$ electrolyte. All data were recorded at a rotation rate of 2000 rpm at a scan rate of 5 mV s^{-1}.

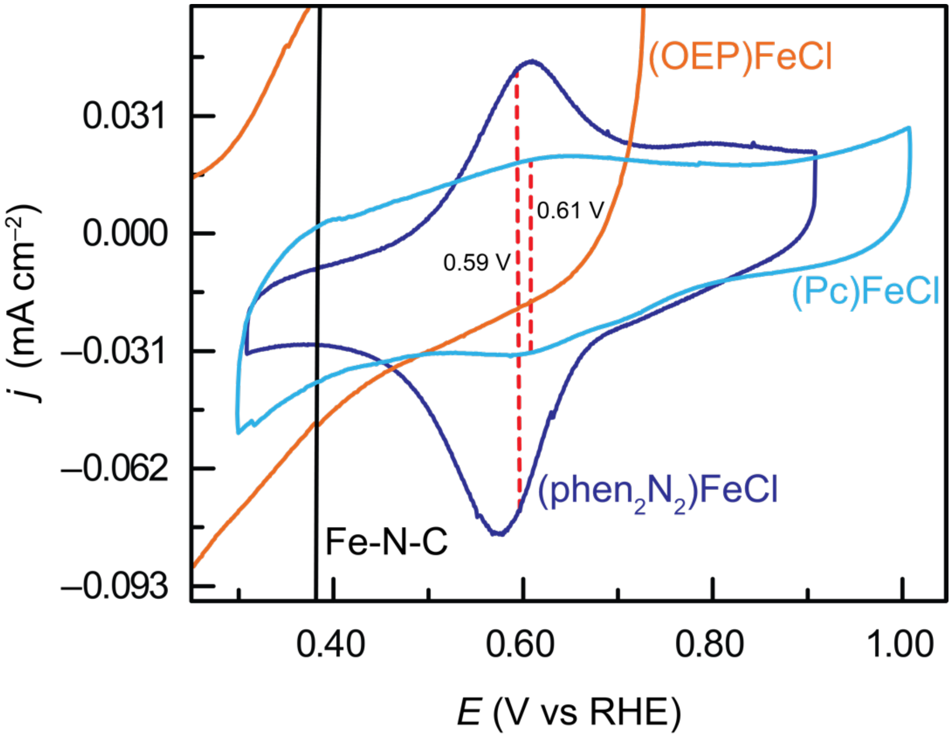

Supplementary Figure 22. Figure 6 expansion. Expansion of Figure 6 to show the (phen$_2$N$_2$)FeCl and (Pc)FeCl Fe(III/II) redox waves. The cyclic voltammograms of (phen$_2$N$_2$)FeCl (blue), Fe-N-C (black), (Pc)FeCl (aqua), and (OEP)FeCl (orange) inks were obtained in O_2-free 0.1 M $HClO_4$ electrolyte. All data were recorded at a rotation rate of 2000 rpm at a scan rate of 5 mV s^{-1}.

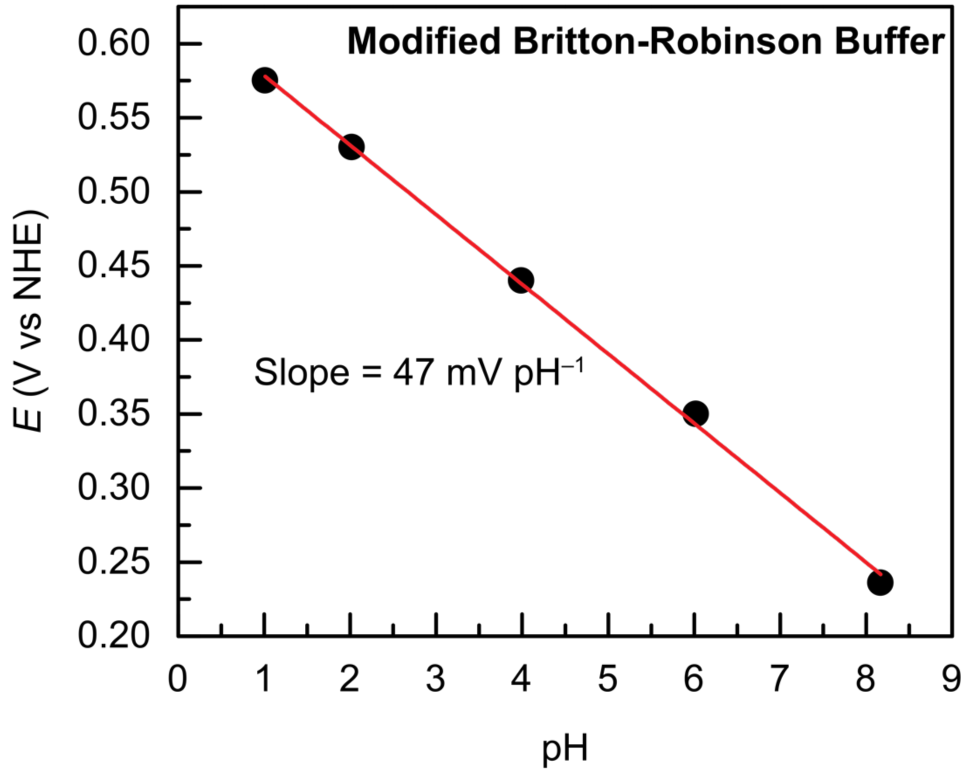

Supplementary Figure 23. pH dependence of the (phen$_2$N$_2$)FeCl Fe(III/II) redox couple. The quasi-Nernstian pH dependence of the Fe(III/II) redox potential of the (phen$_2$N$_2$)FeCl/Nafion/Vulcan ink is shown across a range of pH values in a modified aqueous Britton-Robinson buffer (50 mM each of $NaHSO_4$, Na_2HPO_4 and $B(OH)_3$). pH values were adjusted by adding concentrated aqueous $HClO_4$ and/or NaOH.

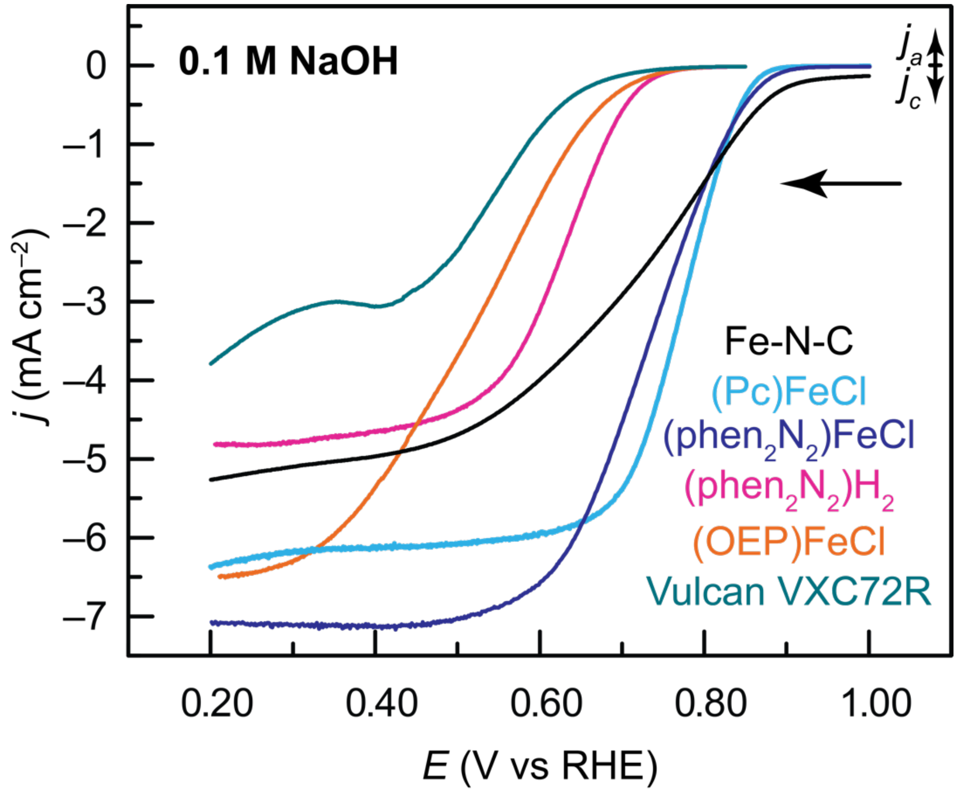

Supplementary Figure 24. Linear voltammograms of molecular catalysts and Fe-N-C. Linear sweep voltammograms of Fe-N-C (black), (phen$_2$N$_2$)FeCl (blue), (Pc)FeCl (aqua), (OEP)FeCl (orange), (phen$_2$N$_2$)H$_2$ (magenta), and Vulcan carbon (teal). Data were recorded in O_2-saturated 0.1 M NaOH at a rotation rate of 2000 rpm and a scan rate of 5 mV s^{-1}.

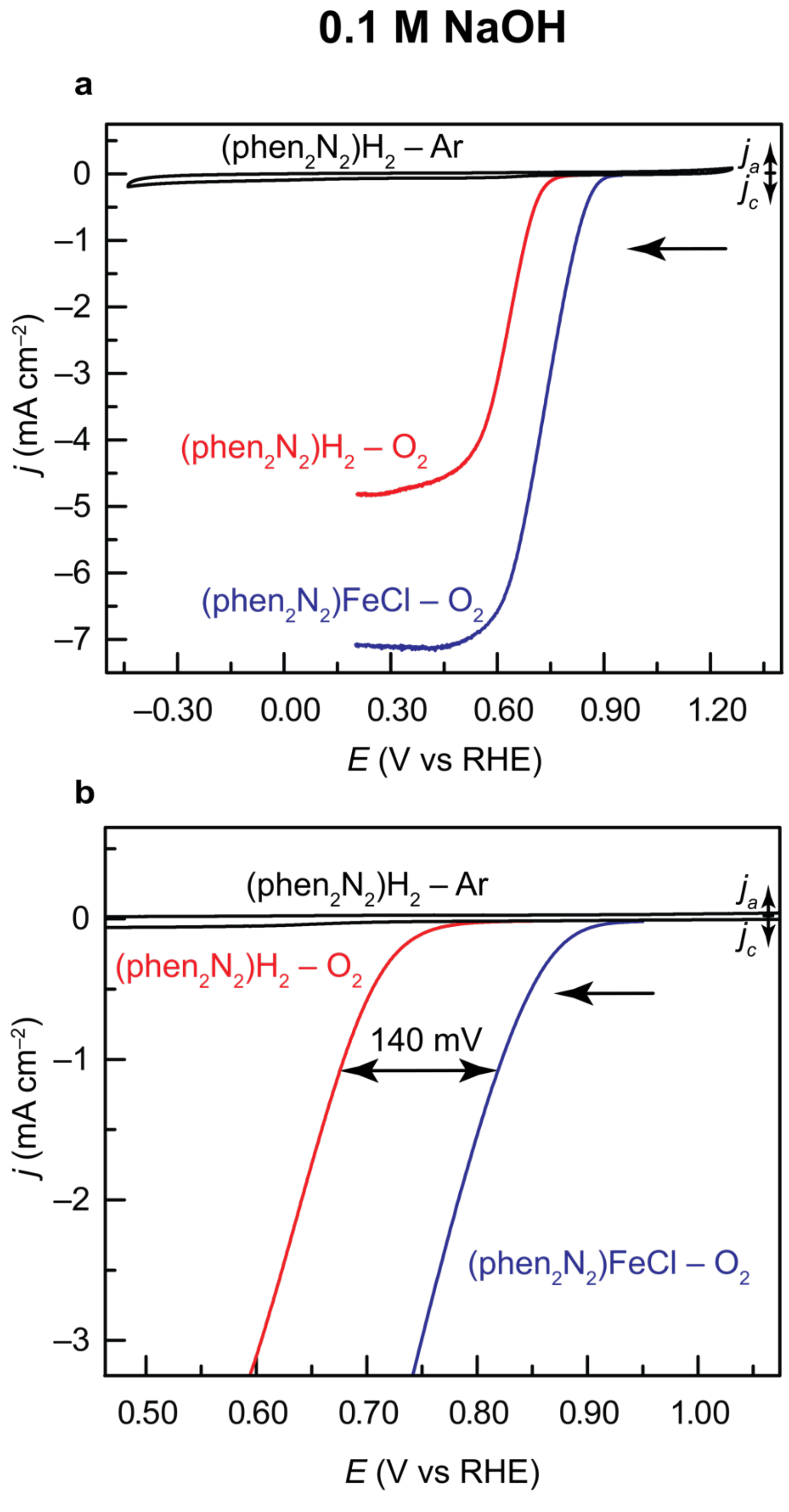

Supplementary Figure 25. Comparative (phen$_2$N$_2$) ORR traces. Linear sweep voltammogram traces for (phen$_2$N$_2$)FeCl and (phen$_2$N$_2$)H$_2$ inks from Supplementary Figure 24 (a). Expansion of (a) in the onset region (b). The data were recorded at a rotation rate of 2000 rpm and a scan rate of 5 mV s^{-1} in 0.1 M NaOH.

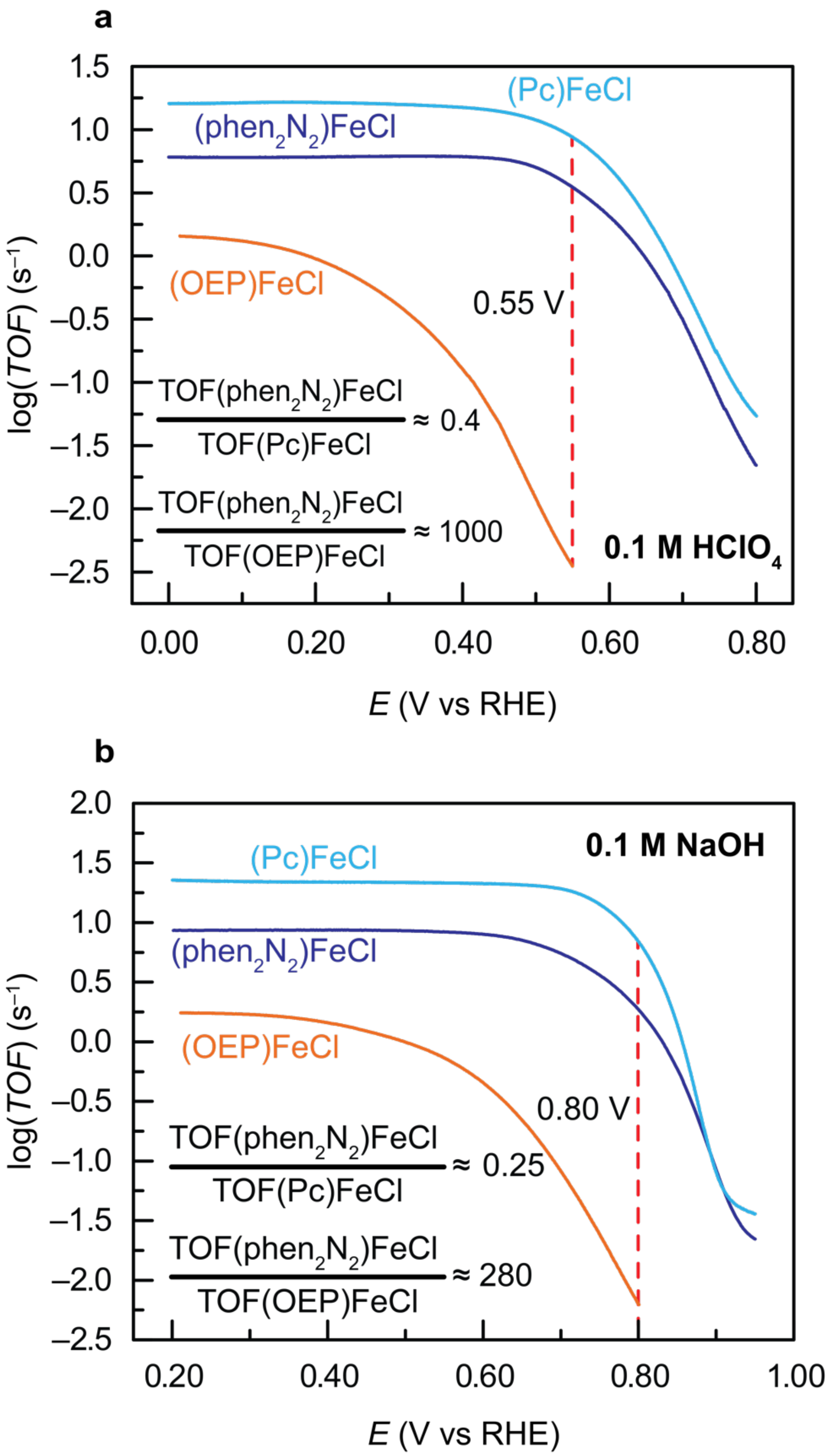

Supplementary Figure 26. Potential-dependent TOF values for molecular ORR catalysts. Per-site TOF values as a function of potential in 0.1 M HClO4 (**a**) and 0.1 M NaOH (**b**) for $(phen_2N_2)FeCl$ (blue), (Pc)FeCl (aqua), and (OEP)FeCl (orange). The data was generated by dividing ORR linear sweep voltammograms by the electroactive catalyst loading (Supplementary Table 12). The data were recorded at a rotation rate of 2000 rpm and a scan rate of 5 mV s^{-1}.

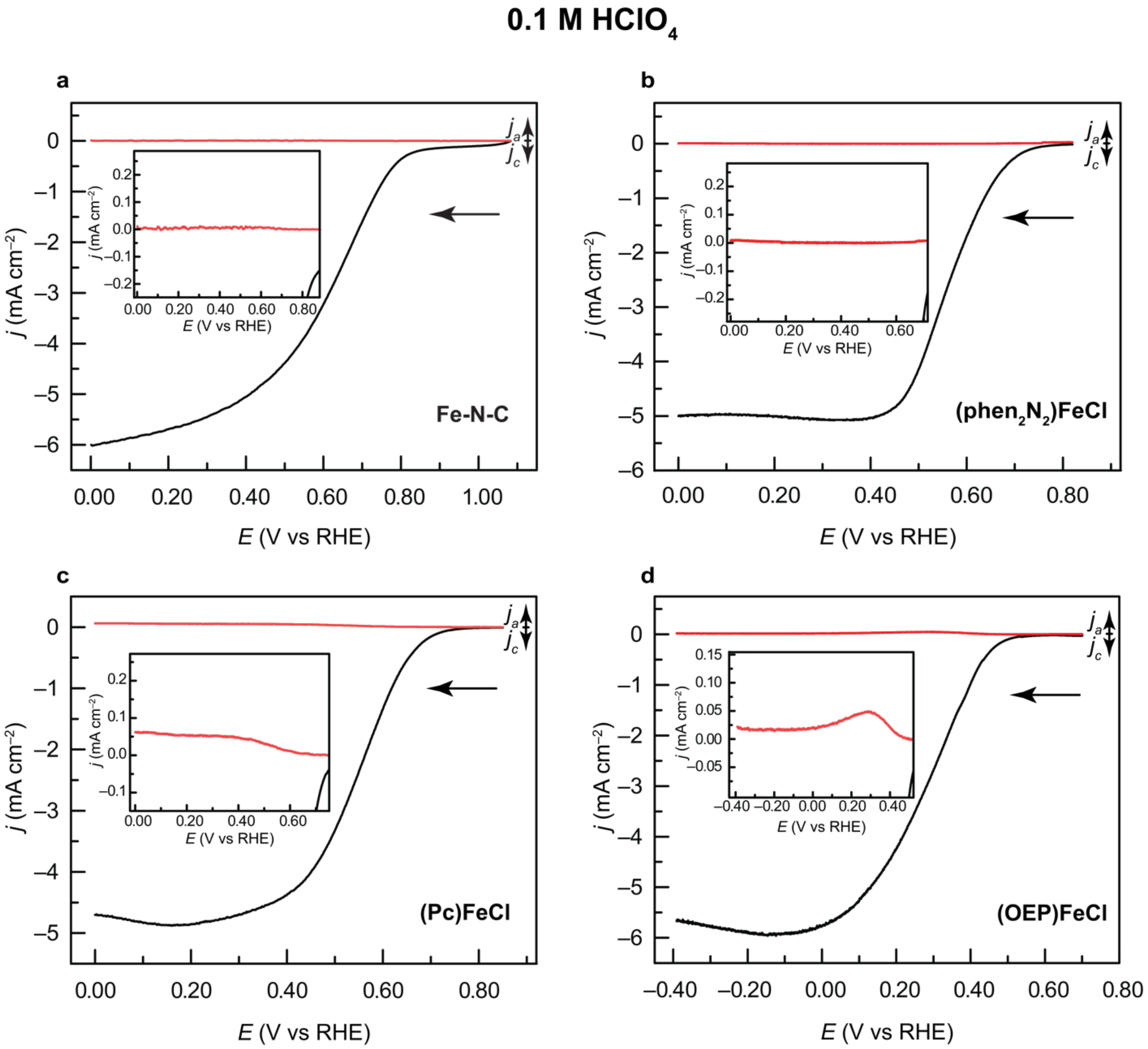

Supplementary Figure 27. Rotating ring disk electrode voltammograms in acid. RRDE plots of Fe-N-C (**a**), (phen$_2$N$_2$)FeCl (**b**), (Pc)FeCl (**c**), and (OEP)FeCl (**d**) in O_2-saturated 0.1 M $HClO_4$ aqueous electrolyte. All data were recorded at a rotation rate of 2000 rpm and 5 mV s^{-1}.

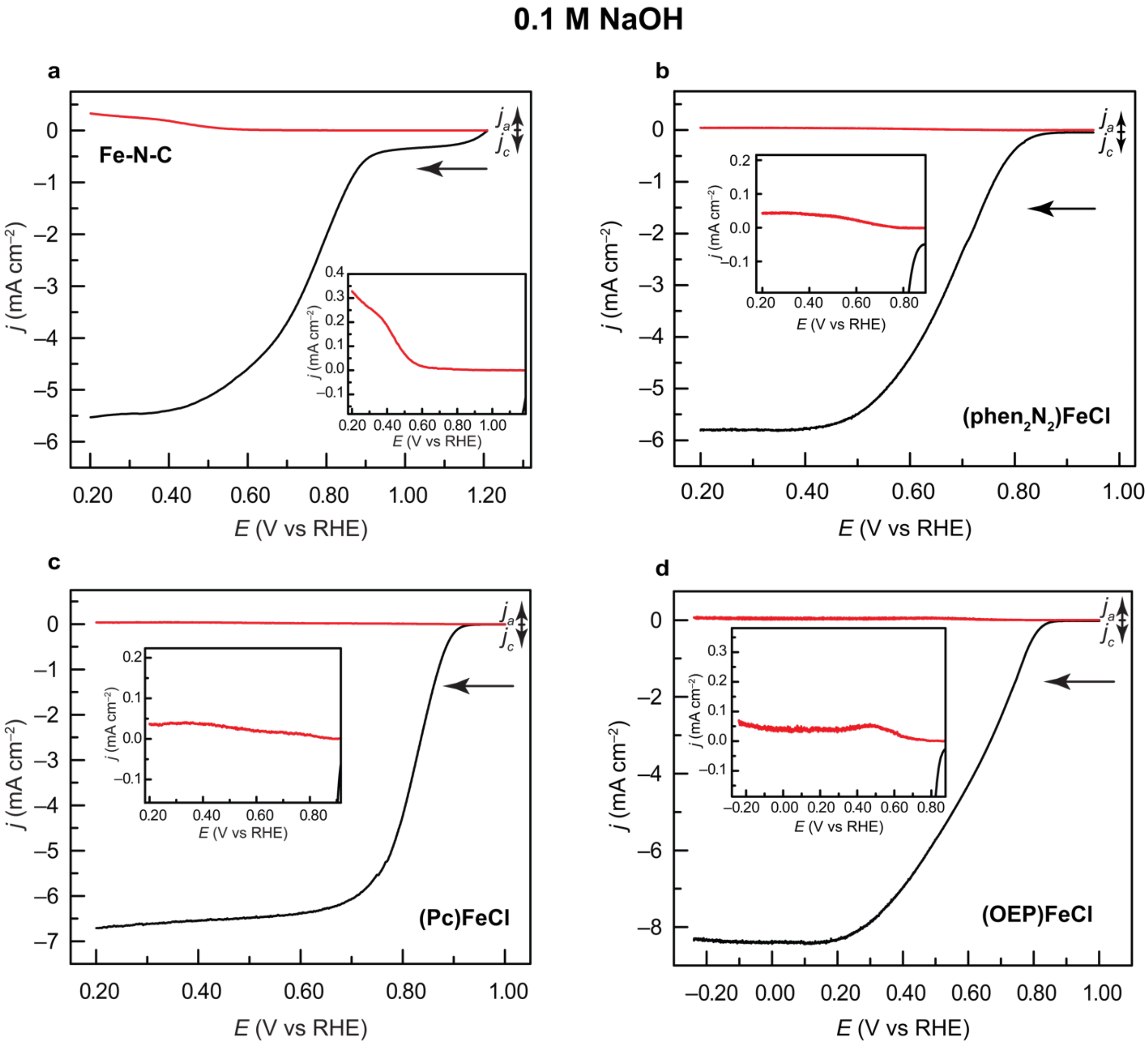

Supplementary Figure 28. Rotating ring disk electrode voltammograms in base. RRDE plots of Fe-N-C (**a**), $(phen_2N_2)$FeCl (**b**), (Pc)FeCl (**c**), and (OEP)FeCl (**d**) in O_2-saturated 0.1 M NaOH aqueous electrolyte. All data were recorded at a rotation rate of 2000 rpm and a scan rate of 5 mV s^{-1}.

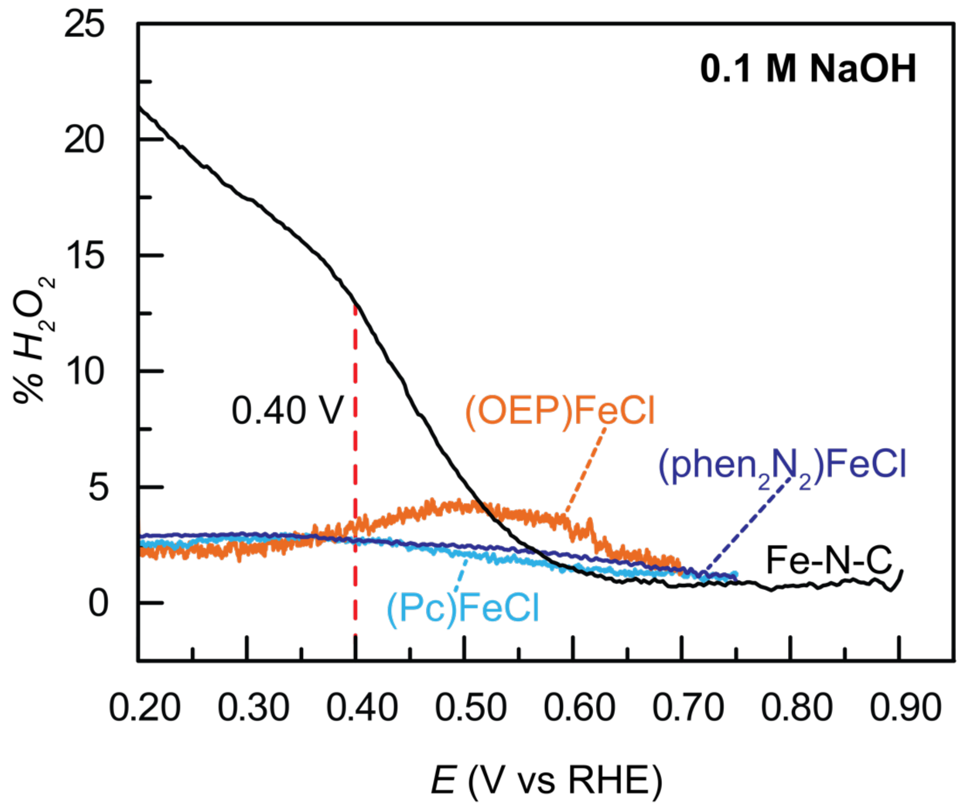

Supplementary Figure 29. Potential-dependent %H_2O_2 production in alkaline media. %H_2O_2 produced during ORR catalysis as a function of potential in alkaline media. The traces are derived from RRDE data in Supplementary Figure 28 for Fe-N-C (black), ($phen_2N_2$)FeCl (blue), (Pc)FeCl (aqua), and (OEP)FeCl (orange) in 0.1 M NaOH aqueous electrolyte.

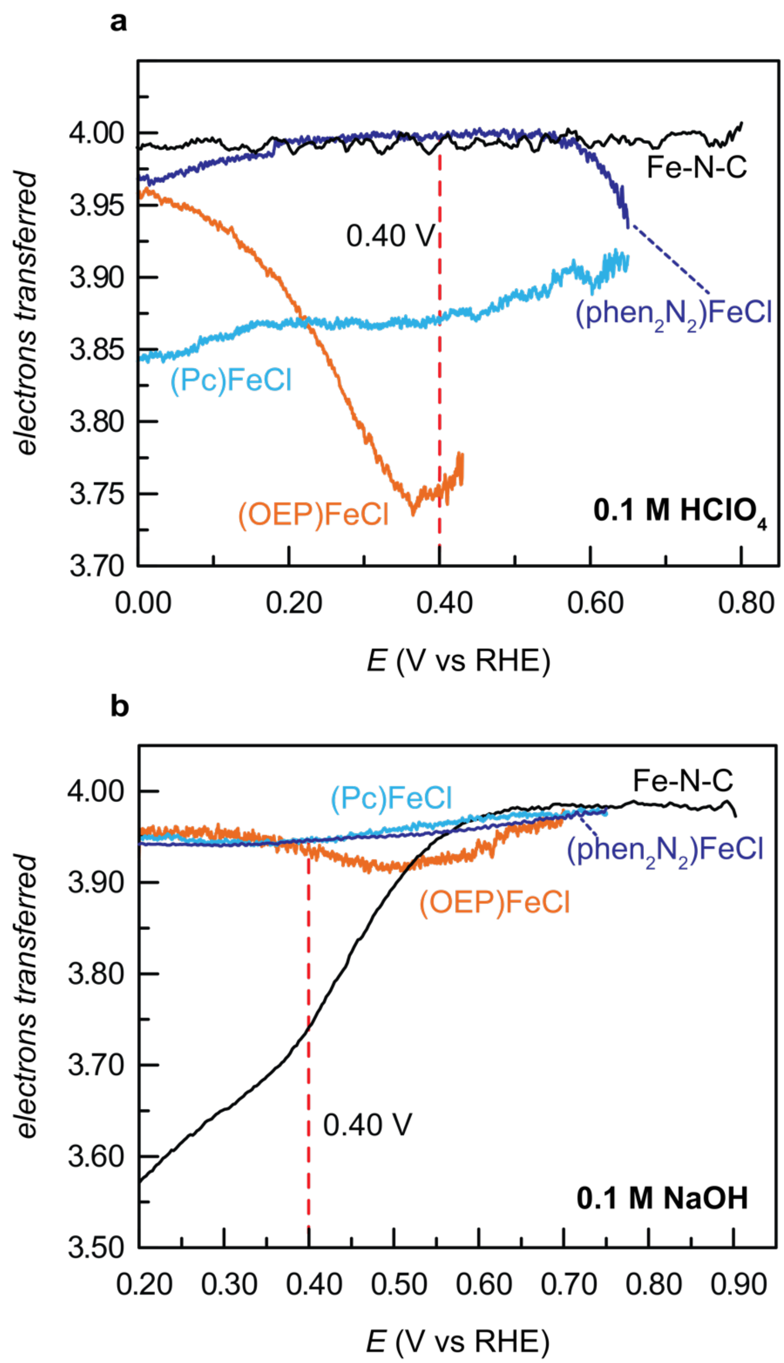

Supplementary Figure 30. Electrons transferred during ORR in alkaline media. Electrons transferred during ORR catalysis as a function of potential. The traces are derived from RRDE data (Supplementary Figures 27 and 28) for Fe-N-C (black), (phen$_2$N$_2$)FeCl (blue), (Pc)FeCl (aqua), and (OEP)FeCl (orange) in 0.1 M $HClO_4$ (**a**) and 0.1 M NaOH (**b**) aqueous electrolyte.

Supplementary Tables

Supplementary Table 1. Zero-Field ^{57}Fe Mössbauer Parameters for Fe-N-C Materials.

Complex or material (preparative method)	Spectral Component	Structural Assignment[a]	δ^{b} / mm s^{-1}	$\lvert\Delta E_Q\rvert$, / mm s^{-1}	T / K	Proposed Active Site for ORR	Reference
Fe-N-C (pyrolyzed ZIF-8 MOF)	D1 (71%)	–	0.46	1.08	90	Fe-N_4	This work
	D2 (29%)	–	0.37	3.06			
$Fe(OAc)_2$, carbon black (pyrolyzed)[2]	D1	$Fe^{II}N_4$, LS	0.39	1.16	–	Fe-N_4/ N-Fe-N_{2+2}	2
	D2	$Fe^{II}N_{2+2}$, IS	0.36	2.40			
(TPP)FeCl (pyrolyzed)[3]	D1[g]	$Fe^{II}N_4$, LS	0.63	0.96	300	Fe-N_4	3
T(p-OCH_3) PPFeCl, iron oxalate (pyrolyzed)[4]	D1	$Fe^{II}N_4$, LS	0.33	0.90	298	Fe-N_4	4
	D2	$Fe^{II}N_4$, Distorted	0.37	2.65			
Fe-N-C (pyrolyzed ZIF-8 MOF)[6]	D1	$Fe^{II}N_4$, LS	0.36	0.98	293	–	6
	D2	–	0.40	2.59			
Fe-N-C (pyrolyzed Phen/PANI[c])[7]	D1	$Fe^{II}N_4$, LS	0.30	0.86	70	Fe-N_4/ N-Fe-N_{2+2}	7
	D2	$Fe^{II}N_4$, PcFe-like[d]	0.27	2.56			
T(p-OCH_3) PPFe[e], carbon black (pyrolyzed)[8]	D1	$Fe^{II}N_4$, LS	0.41	0.61	77	–	8
(TPP)FeCl[f] (pyrolyzed)[9]	D1	$Fe^{II}N_4$, LS	0.33	1.21	77	–	9
Fe-N-C (pyrolyzed ZIF-8 MOF)[10]	D1	$Fe^{II}N_4$, LS	0.37	0.74	298	Fe-N_4	10
	D2	$Fe^{II}N_4$, IS	0.32	2.63			
T(p-OCH_3) PPFeCl, iron oxalate (pyrolyzed)[11]	D1	$Fe^{II}N_4$, LS	0.32	0.81	298	Fe-N_4	11
	D2	$Fe^{II}N_4$, IS	0.36	2.53			

[a]HS = high spin, IS = intermediate spin, LS = low spin. [b]Relative to metallic iron foil. [c]Polyaniline. [d]iron(II) phthalocyanine. [e]Iron 5,10,15,20-tetra-(4-methoxyphenyl)porphyin. Oxidation state and axial ligand are unspecified. [f]5,10,15,20-tetraphenylporphyin iron(III) chloride. [g]The D1 spectral component is designated as DIII within the paper.

Supplementary Table 2. Zero-Field ^{57}Fe Mössbauer Parameters for Molecular Complexes.

Complex[a] (spin state)	δ^b / mm s^{-1}	$\lvert\Delta E_Q\rvert$ / mm s^{-1}	T / K	Reference
$(phen_2N_2)FeCl$ (S=3/2)	0.39	3.06	90	This work
$[(phen_2N_2)Fe]_2O$ (S=5/2, presumed)	0.45	0.87	90	This work
(OEP)FeCl (S=5/2)	0.30	0.60	90	This work
$[(OEP)Fe]_2O$ (S=5/2)	0.41	0.67	90	This work
(Pc)FeCl (S=3/2)	0.28	2.95	90	This work
$[(Pc)Fe]_2O$ (S=5/2)	0.24	1.26	90	This work
(TADBC)FeCl • 2 THF (S=3/2)[5]	0.22	3.20	80	5
(OEP)FeCl (S=5/2)[12]	0.41	0.93	4.2	12
(TPP)FeCl (S=5/2)[12]	0.30	0.48	4.2	12
(Pc)Fe (S=1)[13]	0.31	2.60	77	13
$(OEP)Fe(ClO_4)$ (S=3/2)[14]	0.37	3.48	115	14
(TEtPrP)FeI (S=5/2)[15]	0.32	0.96	77	15
(EtioPc)FeI (S=3/2)[16]	0.29	3.25	70	16
(OEP)Fe (S=1)[17]	0.62	1.71	4.2	17
(OETPP)FeCl (S=5/2, 3/2) admixture[18]	0.35	0.95	280	18
(Pc)FeCl (S=5/2)[19]	0.28	2.56	77	19
$[(Pc)Fe]_2O$ (S=5/2)[20]	0.26	1.26	4.2	20

[a]OEP, TPP, TEtPrP, EtioPc, OETPP, TADBC, and Pc refer to 2,3,7,8,12,13,17,18-octaethylporphyrin, 5,10,15,20-tetraphenylporphyin, *meso*-tetrakis(1-ethylpropyl)porphyrin, 3,6,13,16-tetraethyl-2,7,12,17-tetramethylporphycene, 2,3,7,8,12,13,17,18-octaethyl-5,10,15,20-tetraphenylporphyrin, 5,14-Dihydro-5,9,14,18-tetraaza-di(2,2-dimethyl-[5,6]benzo-[1,3]dioxolo)[a,h]cyclotetradecene, and phthalocyanine, respectively. [b]Relative to metallic iron foil.

Supplementary Table 3. Ratios of elements in molecular model samples.

Sample	%N : %Fe	%Cl : %Fe
$(phen_2N_2)FeCl$	6.1	0.9
$[(phen_2N_2)Fe]_2O$	6.3	0.5
(OEP)FeCl	4.0	1.3
$[(OEP)Fe]_2O$	4.1	–
(Pc)FeCl	7.6	1.1
$[(Pc)Fe]_2O$	7.8	–

Supplementary Table 4. Relative populations of nitrogen environments in Fe-N-C.

	% Pyridinic N	% M-N	% Pyrrolic/Graphitic N	% Oxidized N
Fe-N-C	42.01	33.19	13.44	11.34

Supplementary Table 5. XPS N 1s Peak Parameters and Assignments.

Sample	Peak Assignment	N 1s Binding Energy / eV	FWHM / eV	Area Ratio
Fe-N-C	Pyridinic N	398.3	1.60	3.72:1
	$M\text{-}N_x$	399.9	2.19	2.95:1
	Graphitic/pyrrolic N	401.3	1.98	1.19:1
	Oxidized N	404.0	3.36	1:1
$(phen_2N_2)H_2$	Bridging N	397.9	1.58	1:1
	Pyridinic N	399.5	2.42	2.14:1
	π Satellite	404.3	3.08	−
$[(phen_2N_2)Fe]_2O$	Bridging N	398.3	2.01	1:1
	Pyridinic N	399.6	2.46	1.92:1
	π Satellite	403.6	3.10	−
$(phen_2N_2)FeCl$	Bridging N	398.5	2.22	1:1
	Pyridinic N	399.9	2.14	1.90:1
	π Satellite	402.6	3.24	−
$[(OEP)Fe]_2O$	Pyrrolic N	397.6	1.20	−
	π Satellite	399.9	3.20	−
(OEP)FeCl	Pyrrolic N	398.0	1.13	−
	π Satellite	400.2	3.28	−
$[(Pc)Fe]_2O$	Pyrrolic N	398.4	1.15	−
	π Satellite	400.1	1.42	−
(Pc)FeCl	Pre-peak	396.8	0.64	−
	Pyrrolic N	398.5	1.21	−
	π Satellite	400.1	1.66	−

Supplementary Table 6. XPS Fe 2p Peak Parameters and Assignments.

Sample	Peak Assignment	Fe 2p Binding Energy / eV	FWHM / eV
Fe-N-C	$2p_{3/2}$ Fe(II) Main peak	709.5	3.84
	$2p_{3/2}$ Fe(III) Main peak	713.9	6.28
$[(phen_2N_2)Fe]_2O$	$2p_{3/2}$ Main peak	710.8	3.07
	$2p_{3/2}$ Multiplet peak	713.3	3.36
	$2p_{1/2}$ Main peak	724.5	2.68
	$2p_{1/2}$ Multiplet peak	727.1	2.83
$(phen_2N_2)FeCl$	$2p_{3/2}$ Main peak	710.5	2.70
	$2p_{3/2}$ Multiplet peak	712.8	3.35
	$2p_{1/2}$ Main peak	723.9	2.26
	$2p_{1/2}$ Multiplet peak	725.8	3.39
$[(OEP)Fe]_2O$	Pre-peak	707.1	1.22
	$2p_{3/2}$ Main peak	709.2	1.92
	$2p_{3/2}$ Multiplet peak	711.1	3.00
	$2p_{3/2}$ Satellite peak	716.5	2.65
	$2p_{1/2}$ Main peak	723.2	3.13
(OEP)FeCl	Pre-peak	707.3	1.47
	$2p_{3/2}$ Main peak	710.1	3.36
	$2p_{3/2}$ Multiplet peak	712.5	2.74
	$2p_{1/2}$ Main peak	724.2	3.37
$[(Pc)Fe]_2O$	$2p_{3/2}$ Main peak	708.9	1.65
	$2p_{3/2}$ Multiplet peak	710.5	3.37
	$2p_{1/2}$ Main peak	722.0	1.75
	$2p_{1/2}$ Multiplet peak	723.8	3.38
(Pc)FeCl	Pre-peak	707.8	1.13
	$2p_{3/2}$ Main peak	710.0	2.4
	$2p_{3/2}$ Multiplet peak	712.2	3.36
	$2p_{1/2}$ Main peak	723.5	3.28
	$2p_{1/2}$ Satellite peak	727.2	3.36

Supplementary Table 7. Proportions of Fe(II) and Fe(III) in Fe-N-C from XPS.

	**% Fe(II)**	**% Fe(III)**
Fe-N-C	56.79	43.21

Supplementary Table 8. XPS O 1s Peak Parameters and Assignments.

Sample	Peak Assignment	O 1s Binding Energy / eV	FWHM / eV
Fe-N-C	Aromatic C=O	531.3	2.41
	Aliphatic C–O	532.3	1.15
	Aromatic C–O	533.1	1.92
	Adsorbed H_2O	534.4	3.50
$[(phen_2N_2)Fe]_2O$	Fe–O–Fe	529.5	1.81
	Aromatic C=O	530.8	1.73
	Aliphatic C–O	532.0	1.81
	Aromatic C–O	533.0	2.05
	Adsorbed H_2O	533.9	2.34
$(phen_2N_2)FeCl$	Aromatic C=O	530.9	1.92
	Aliphatic C–O	532.1	1.73
	Aromatic C–O	533.1	2.14
$[(OEP)Fe]_2O$	Fe–O–Fe	528.7	1.42
	Aromatic C=O	531.3	2.38
	Aliphatic C–O	531.7	1.18
	Aromatic C–O	533.0	2.05
(OEP)FeCl	Aromatic C=O	531.4	2.23
	Aliphatic C–O	532.1	1.34
	Aromatic C–O	533.3	1.97
	Adsorbed H_2O	534.9	3.50
$[(Pc)Fe]_2O$	Fe–O–Fe	529.3	1.34
	Aromatic C=O	531.5	2.58
	Aliphatic C–O	532.0	1.59
	Aromatic C–O	533.3	2.09
(Pc)FeCl	Aromatic C=O	531.4	2.11
	Aliphatic C–O	532.0	0.77
	Aromatic C–O	533.1	2.25

Supplementary Table 9. EXAFS Iron K-edge Fitting Parameters.

Sample	Scattering Path	CN	R / Å	$\Delta\sigma^2$ / $Å^2$	ΔE_o / eV	Pre-edge energy / keV	XANES energy / keV
$[(phen_2N_2)Fe]_2O$	Fe–N	4.0 ± 0.1	1.97 ± 0.08	0.005 ± 0.001	−9.8 ± 4.8	7.1140	7.1280
	Fe–O	1.0 ± 0.4	1.79 ± 0.05	0.001 ± 0.001			
$[(OEP)Fe]_2O$	Fe–N	4.0 ± 0.2	2.06 ± 0.04	0.005 ± 0.001	−3.0 ± 5.6	7.1140	7.1230
	Fe–O	0.9 ± 0.1	1.79 ± 0.06	0.001 ± 0.001			
$[(Pc)Fe]_2O$	Fe–N	4.0 ± 0.5	1.94 ± 0.02	0.005 ± 0.001	−3.6 ± 1.6	7.1120	7.1249
	Fe–O	1.0 ± 0.3	1.73 ± 0.07	0.005 ± 0.001			
Fe-N-C	Fe–N	4.0 ± 0.1	1.94 ± 0.05	0.005± 0.001	−5.5 ± 5.0	7.1142	7.1257
	Fe–O	1.7 ± 0.3	2.04 ± 0.03	0.001 ± 0.001			

Supplementary Table 10. Nominal and Electroactive Catalyst Loadings[a].

Catalyst Ink	Catalyst Mass / mg cm^{-2}	Total Mass / mg cm^{-2}	Moles catalyst / 1 x 10^{-7} mol cm^{-2}	Electroactive Moles[b] / 1 x 10^{-7} mol cm^{-2}
$(phen_2N_2)FeCl$[c]	0.056	0.18	1.2	0.085
(OEP)FeCl[c]	0.056	0.18	0.90	0.39
(Pc)FeCl[d]	0.087	0.23	1.4	0.030
Fe-N-C (11 mg mL^{-1})[e]	0.56	0.56	–	–
Fe-N-C (4.7 mg mL^{-1})[e]	0.24	0.24	–	–
$(phen_2N_2)H_2$[f]	0.046	0.21	1.2	–
Vulcan[g]	0	0.51	–	–

[a]Mass loadings calculated for 10 uL aliquots dropcast on glassy carbon RDEs based upon stock ink concentrations for each catalyst which were in the range of 1-1.5 mg mL^{-1} catalyst and 2.5-4.0 mg mL^{-1} Vulcan carbon powder. [b]Electroactive moles were calculated by integrating the Fe(III/II) redox wave visible in traces recorded under inert atmosphere in 0.1 M $HClO_4$ at a scan rate of 5 mV s^{-1}. [c]$(phen_2N_2)FeCl$ and (OEP)FeCl inks were prepared as 1.1 mg mL^{-1} catalyst mixtures in 2.5 mg mL^{-1} Vulcan carbon. [d](Pc)FeCl inks were prepared as 1.7 mg mL^{-1} catalyst mixtures in 2.9 mg mL^{-1} Vulcan carbon. [e]Fe-N-C catalyst ink was prepared without Vulcan carbon and had a concentration of either 11 or 4.7 mg mL^{-1}. [f]$(phen_2N_2)H_2$ ink was prepared as a 0.9 mg mL^{-1} catalyst mixture in 2.7 mg mL^{-1} Vulcan carbon. [g]Bare Vulcan carbon ink was prepared in a stock concentration of 10 mg mL^{-1}.

Supplementary Table 11. Electrocatalytic Performance Metrics and Metal Loadings.

Material/Complex (Carbon support)	Electrolyte	$E_{1/2}$ / V[a]	Onset / V[b]	Max %H_2O_2	Total Metal Loading / µg cm^{-2}
($phen_2N_2$)FeCl (Vulcan)	0.1 M $HClO_4$	0.59	0.75	1.6	6.6
	0.1 M NaOH	–	0.89	3.0	
(OEP)FeCl (Vulcan)	0.1 M $HClO_4$	0.27	0.45	13.2	5.0
	0.1 M NaOH	–	0.74	3.9	
(Pc)FeCl (Vulcan)	0.1 M $HClO_4$	0.61	0.72	7.8	8.0
	0.1 M NaOH	–	0.89	2.9	
Fe-N-C	0.1 M $HClO_4$	–	0.85	0.6	6.0
	0.1 M NaOH	–	0.90	21.4	
($phen_2N_2$)H_2 (Vulcan)	0.1 M NaOH	–	0.75	26.5	–

[a]V vs RHE. [b]Defined as the potential corresponding to an ORR current density of −0.1 mA cm^{-2}.

Supplementary Table 12. Electrocatalytic Properties of Molecular Catalyst Inks.

Catalyst	Electroactive Moles / 1×10^{-7} mol cm^{-2}	TOF in 0.1 M $HClO_4$ @ 0.55 V vs RHE / s^{-1}	TOF in 0.1 M NaOH @ 0.80 V vs RHE / s^{-1}
($phen_2N_2$)FeCl	0.085	3.5	1.8
(OEP)FeCl	0.39	0.0035	0.0062
(Pc)FeCl	0.030	8.7	6.9

Supplementary Notes

Supplementary Note 1: Cartesian Coordinates for Computed Structures

1: Optimized structure of (phen$_2$N$_2$)Fe(III)Cl, Quartet state, S = 3/2
Energy = −2974.494966 Hartree

C	-3.64212	-0.84553	-2.43012
C	-3.58572	-2.17139	-1.89145
C	-4.68957	-2.95418	-1.43309
C	-2.30127	-2.73214	-1.80308
C	-2.10677	-4.02395	-1.25387
C	-4.49990	-4.21375	-0.89746
C	-3.19303	-4.77919	-0.78253
C	-2.85818	-6.05126	-0.21658
N	-1.15251	-2.08744	-2.19106
N	-0.79875	-4.43757	-1.19197
C	-1.19135	-0.80247	-2.64227
C	-2.49031	-0.19006	-2.78504
N	-0.10909	-0.05641	-2.93483
C	-0.46660	-5.61663	-0.59518
C	-1.54809	-6.44783	-0.12450
N	0.79264	-6.04599	-0.38673
C	1.87364	-5.28650	-0.64754
N	1.84042	-4.06549	-1.25143
C	2.97929	-3.30731	-1.36860
C	4.30654	-5.04085	-0.37669
C	4.25158	-3.72971	-0.94993
C	1.14909	-0.47320	-2.69621
C	2.22065	0.47298	-2.89354
C	3.85932	-1.12232	-2.06074
C	2.78512	-2.01576	-1.91869
N	1.48694	-1.71609	-2.25178
C	5.34486	-2.82584	-1.11958
C	5.15552	-1.56659	-1.65615
C	3.16231	-5.78327	-0.22935
C	3.52325	0.16360	-2.59395
H	-5.69207	-2.53975	-1.50270
H	-5.35352	-4.78805	-0.54657
H	-4.60514	-0.35297	-2.54238
H	-3.64957	-6.69765	0.15565
H	6.33852	-3.13952	-0.80999
H	5.26121	-5.44084	-0.04313
H	-1.27069	-7.39626	0.32255
H	-2.50812	0.82295	-3.17207
H	1.94085	1.44886	-3.27523
H	4.30661	0.90237	-2.74625

H	3.17762	-6.76838	0.22413
H	6.00059	-0.89205	-1.76773
Fe	0.35929	-3.25387	-2.14333
Cl	0.43570	-4.12643	-4.21615

2: Optimized structure of (phen$_2$N$_2$)Fe(III)Cl, Doublet state, S = 1/2
Energy = −2974.483751 Hartree

C	-3.65207	-0.84891	-2.44479
C	-3.60095	-2.17603	-1.90535
C	-4.70371	-2.96655	-1.46066
C	-2.31688	-2.73361	-1.79893
C	-2.12262	-4.02385	-1.25020
C	-4.51373	-4.22845	-0.92404
C	-3.20742	-4.78963	-0.79384
C	-2.86707	-6.06173	-0.22801
N	-1.16124	-2.08709	-2.16461
N	-0.80984	-4.42103	-1.17175
C	-1.19741	-0.80264	-2.62952
C	-2.49604	-0.19201	-2.78624
N	-0.10961	-0.06437	-2.91484
C	-0.47380	-5.60884	-0.58535
C	-1.55399	-6.44862	-0.12579
N	0.78805	-6.02661	-0.37800
C	1.87548	-5.27325	-0.63784
N	1.84143	-4.03586	-1.21588
C	2.98964	-3.29756	-1.35167
C	4.31533	-5.05107	-0.39467
C	4.26476	-3.73523	-0.95689
C	1.15309	-0.47475	-2.68033
C	2.22330	0.46600	-2.90257
C	3.87126	-1.12016	-2.07059
C	2.79568	-2.00764	-1.90090
N	1.49153	-1.71042	-2.20613
C	5.35814	-2.83637	-1.14603
C	5.16842	-1.57488	-1.68331
C	3.16424	-5.78449	-0.24110
C	3.53087	0.16003	-2.61416
H	-5.70846	-2.55890	-1.53866
H	-5.37035	-4.80474	-0.58364
H	-4.61405	-0.35780	-2.57122
H	-3.65630	-6.71699	0.13305
H	6.35592	-3.15319	-0.85300
H	5.27014	-5.46512	-0.07935
H	-1.27584	-7.39998	0.31480

H	-2.51314	0.81859	-3.17986
H	1.94263	1.43510	-3.30081
H	4.31270	0.89537	-2.78884
H	3.17885	-6.77712	0.19595
H	6.01836	-0.90883	-1.80897
Fe	0.34901	-3.19017	-1.98984
Cl	0.50488	-4.03434	-4.02279

3: Optimized structure of (phen$_2$N$_2$)Fe(III)Cl, Sextet state, S = 5/2
Energy = −2974.474072 Hartree

C	-3.62744	-0.82637	-2.39000
C	-3.56098	-2.15341	-1.85393
C	-4.65352	-2.90132	-1.32094
C	-2.28292	-2.74274	-1.84765
C	-2.08689	-4.04397	-1.29435
C	-4.46370	-4.16083	-0.78525
C	-3.16845	-4.75860	-0.74605
C	-2.84339	-6.03219	-0.17655
N	-1.15749	-2.11538	-2.30738
N	-0.79797	-4.50260	-1.29258
C	-1.19519	-0.81766	-2.72034
C	-2.48861	-0.18267	-2.80288
N	-0.10836	-0.07521	-3.01483
C	-0.46573	-5.66298	-0.66044
C	-1.54262	-6.46530	-0.13224
N	0.79716	-6.09024	-0.45642
C	1.88240	-5.33145	-0.71235
N	1.85296	-4.12846	-1.35140
C	2.96368	-3.33179	-1.40704
C	4.28853	-5.02613	-0.33521
C	4.22375	-3.71625	-0.91159
C	1.15324	-0.48702	-2.77386
C	2.21613	0.47950	-2.91153
C	3.83215	-1.11142	-2.02190
C	2.76812	-2.03071	-1.96107
N	1.49382	-1.74165	-2.36635
C	5.30048	-2.78426	-1.00584
C	5.11134	-1.52520	-1.54289
C	3.16116	-5.80129	-0.23615
C	3.50567	0.17814	-2.55370
H	-5.64663	-2.45909	-1.32951
H	-5.30764	-4.70818	-0.37285
H	-4.58913	-0.32154	-2.44649
H	-3.63640	-6.64674	0.24342

H	6.28032	-3.07442	-0.63485
H	5.23805	-5.39499	0.04586
H	-1.27168	-7.41006	0.32667
H	-2.51307	0.83469	-3.17829
H	1.94220	1.46127	-3.28254
H	4.28707	0.92834	-2.65104
H	3.18213	-6.78101	0.22895
H	5.94263	-0.82646	-1.59402
Fe	0.37380	-3.41823	-2.53526
Cl	0.45025	-4.26783	-4.56446

4: Optimized structure of [(phen$_2$N$_2$)Fe(III)]$^+$, Quartet state, S = 3/2
Energy = −2514.071912 Hartree

C	-3.76538	-1.10294	-2.89686
C	-3.62586	-2.50380	-2.62471
C	-4.65848	-3.49470	-2.60183
C	-2.31801	-2.93037	-2.35430
C	-2.03014	-4.28937	-2.07394
C	-4.37735	-4.82172	-2.32825
C	-3.04200	-5.25963	-2.05658
C	-2.60207	-6.59288	-1.76537
N	-1.21961	-2.09777	-2.34210
N	-0.69899	-4.55547	-1.83482
C	-1.33895	-0.76219	-2.61077
C	-2.66787	-0.27330	-2.88941
N	-0.30877	0.11112	-2.63436
C	-0.26626	-5.82474	-1.56659
C	-1.27254	-6.85863	-1.53177
N	1.02102	-6.16508	-1.33977
C	2.05096	-5.29147	-1.36174
N	1.92795	-3.95079	-1.60189
C	3.03096	-3.12453	-1.62510
C	4.48552	-4.96199	-1.13855
C	4.34497	-3.55965	-1.40218
C	0.97834	-0.22896	-2.40611
C	1.98956	0.79813	-2.47895
C	3.76116	-0.80385	-1.97068
C	2.74312	-1.76555	-1.90551
N	1.40736	-1.49305	-2.10908
C	5.38258	-2.57560	-1.46341
C	5.10149	-1.24862	-1.73723
C	3.38472	-5.78709	-1.12071
C	3.32238	0.52781	-2.27079
H	-5.68268	-3.19230	-2.80637

H	-5.18269	-5.55220	-2.31988
H	-4.74976	-0.69416	-3.11359
H	-3.32841	-7.40182	-1.73154
H	6.41109	-2.88400	-1.29222
H	5.47398	-5.37653	-0.95360
H	-0.93052	-7.86505	-1.31417
H	-2.76375	0.78720	-3.09757
H	1.64929	1.80215	-2.70993
H	4.05289	1.33100	-2.33668
H	3.48230	-6.84994	-0.92578
H	5.91120	-0.52415	-1.77918
Fe	0.35171	-3.02089	-1.95311

5: Optimized structure of $[(phen_2N_2)Fe(III)]^+$, Doublet state, S = 1/2
Energy = −2514.063397

C	-3.76841	-1.09266	-2.89832
C	-3.62502	-2.49163	-2.62648
C	-4.64324	-3.48620	-2.60096
C	-2.31023	-2.92049	-2.35502
C	-2.01877	-4.29558	-2.07348
C	-4.36006	-4.82189	-2.32604
C	-3.03601	-5.27015	-2.05549
C	-2.60060	-6.60335	-1.76367
N	-1.22567	-2.09834	-2.34321
N	-0.70414	-4.55884	-1.83802
C	-1.34891	-0.75950	-2.61287
C	-2.66986	-0.26209	-2.89165
N	-0.30613	0.09898	-2.63287
C	-0.27378	-5.83203	-1.56594
C	-1.27013	-6.86965	-1.52863
N	1.01853	-6.15378	-1.34102
C	2.06067	-5.29479	-1.35957
N	1.93448	-3.95146	-1.60399
C	3.02349	-3.13508	-1.62636
C	4.48849	-4.97212	-1.13681
C	4.34407	-3.57178	-1.40185
C	0.98633	-0.22210	-2.40652
C	1.98678	0.80923	-2.48104
C	3.75544	-0.79320	-1.97303
C	2.73209	-1.75999	-1.90791
N	1.41314	-1.49084	-2.10902
C	5.36699	-2.58336	-1.46423
C	5.08418	-1.24763	-1.73928
C	3.38684	-5.79816	-1.11762

C	3.32082	0.53847	-2.27194
H	-5.66994	-3.19446	-2.80356
H	-5.17205	-5.54363	-2.31945
H	-4.75233	-0.68546	-3.11408
H	-3.32732	-7.41032	-1.72977
H	6.39791	-2.88096	-1.29411
H	5.47642	-5.38473	-0.95178
H	-0.92725	-7.87479	-1.30964
H	-2.76398	0.79798	-3.09952
H	1.64512	1.81212	-2.71189
H	4.05144	1.34001	-2.33678
H	3.48286	-6.86028	-0.92145
H	5.90040	-0.53176	-1.77850
Fe	0.35180	-3.02111	-1.95361

6: Optimized structure of $[(phen_2N_2)Fe(III)]^+$, Sextet state, S = 5/2
Energy = −2514.035495 Hartree

C	-3.72029	-1.11016	-2.98679
C	-3.56849	-2.50312	-2.68107
C	-4.54787	-3.52152	-2.85513
C	-2.30529	-2.88293	-2.18414
C	-2.01362	-4.25966	-1.90071
C	-4.26587	-4.85211	-2.58133
C	-2.98439	-5.25917	-2.11376
C	-2.55608	-6.60329	-1.85566
N	-1.27528	-2.00627	-2.00689
N	-0.74139	-4.52693	-1.48754
C	-1.37715	-0.69731	-2.39423
C	-2.66417	-0.24154	-2.85389
N	-0.34138	0.16659	-2.41154
C	-0.29334	-5.81207	-1.33992
C	-1.25949	-6.87040	-1.48806
N	0.99481	-6.13971	-1.11071
C	2.03159	-5.27699	-1.13458
N	1.89534	-3.92063	-1.25681
C	2.97500	-3.11224	-1.46151
C	4.46441	-4.98790	-1.23275
C	4.30386	-3.58247	-1.46884
C	0.94792	-0.16250	-2.18979
C	1.96627	0.82361	-2.44609
C	3.71948	-0.82649	-2.03835
C	2.68305	-1.73569	-1.74527
N	1.36110	-1.40007	-1.77585
C	5.33979	-2.64166	-1.73073

C	5.05763	-1.31141	-2.00591
C	3.37072	-5.80488	-1.07707
C	3.30042	0.50482	-2.36796
H	-5.53112	-3.24819	-3.22758
H	-5.03140	-5.60590	-2.74233
H	-4.67654	-0.74655	-3.35331
H	-3.26218	-7.42007	-1.97931
H	6.37169	-2.98130	-1.73117
H	5.46449	-5.41215	-1.20380
H	-0.91223	-7.88508	-1.32802
H	-2.75362	0.80608	-3.11897
H	1.63543	1.81574	-2.73237
H	4.05048	1.26109	-2.58354
H	3.47680	-6.87493	-0.93740
H	5.87173	-0.62431	-2.21891
Fe	0.23365	-2.85766	-1.03740

Supplementary References:

1. Mineva, T. *et al.* Understanding active sites in pyrolyzed Fe-N-C catalysts for fuel cell cathodes by bridging density functional theory calculations and ^{57}Fe Mössbauer spectroscopy. *ACS Catal.* **9**, 9359–9371 (2019).
2. Kramm, U. I. *et al.* Structure of the catalytic sites in Fe/N/C-catalysts for O_2-reduction in PEM fuel cells. *Phys. Chem. Chem. Phys.* **14**, 11673–11688 (2012).
3. Bouwkamp-Wijnoltz, A. L. *et al.* On active-site heterogeneity in pyrolyzed carbon-supported iron porphyrin catalysts for the electrochemical reduction of oxygen: an *in situ* Mössbauer study. *J. Phys. Chem. B* **106**, 12993–13001 (2002).
4. Koslowski, U. I., Abs-Wurmbach, I., Fiechter, S. & Bogdanoff, P. Nature of the catalytic centers of porphyrin-based electrocatalysts for the ORR: a correlation of kinetic current density with the site density of Fe−N_4 centers. *J. Phys. Chem. C* **112**, 15356–15366 (2008).
5. Sustmann, R. *et al.* Fe^{III} complexes of 1,4,8,11-tetraaza[14]annulenes as catalase mimics. *Inorg. Chem.* **46**, 11416–11430 (2007).
6. Zitolo, A. *et al.* Identification of catalytic sites for oxygen reduction in iron- and nitrogen-doped graphene materials. *Nat. Mater.* **14**, 937–942 (2015).
7. Kramm, U. I., Lefèvre, M., Larouche, N., Schmeisser, D. & Dodelet, J.-P. Correlations between mass activity and physicochemical properties of Fe/N/C catalysts for the ORR in PEM fuel cell via ^{57}Fe Mössbauer spectroscopy and other techniques. *J. Am. Chem. Soc.* **136**, 978–985 (2014).
8. van Veen, J. A. R., van Baar, J. F. & Kroese, K. J. Effect of heat treatment on the performance of carbon-supported transition-metal chelates in the electrochemical reduction of oxygen. *J. Chem. Soc. Faraday Trans. 1* **77**, 2827–2843 (1981).
9. Blomquist, J., Lång, H., Larsson, R. & Widelöv, A. Pyrolysis behaviour of metalloporphyrins. Part 2.—A Mössbauer study of pyrolysed Fe^{III} tetraphenylporphyrin chloride. *J. Chem. Soc. Faraday Trans.* **88**, 2007–2011 (1992).
10. Kramm, U. I., Lefèvre, M., Bogdanoff, P., Schmeißer, D. & Dodelet, J.-P. Analyzing structural changes of Fe–N–C cathode catalysts in PEM fuel cell by Mößbauer spectroscopy of complete membrane electrode assemblies. *J. Phys. Chem. Lett.* **5**, 3750–3756 (2014).
11. Kramm, U. I. *et al.* On an easy way to prepare metal–nitrogen doped carbon with exclusive presence of MeN_4-type sites active for the ORR. *J. Am. Chem. Soc.* **138**, 635–640 (2016).
12. Dolphin, D. H., Sams, J. R., Tsin, T. B. & Wong, K. L. Moessbauer-Zeeman spectra of some octaethylporphyrinato- and tetraphenylporphinatoiron(III) complexes. *J. Am. Chem. Soc.* **100**, 1711–1718 (1978).
13. Blomquist, J., Moberg, L. C., Johansson, L. Y. & Larsson, R. Mössbauer measurements on iron phthalocyanines. *J. Inorg. Nucl. Chem.* **43**, 2287–2292 (1981).
14. Reed, C. A. *et al.* The missing heme spin state and a model for cytochrome *c'*. The mixed $S = 3/2, 5/2$ intermediate spin ferric porphyrin: perchlorato(meso-tetraphenylporphinato)iron(III). *J. Am. Chem. Soc.* **101**, 2948–2958 (1979).
15. Sakai, T. *et al.* Electronic structures of five-coordinate iron(III) porphyrin complexes with highly ruffled porphyrin ring. *Inorg. Chem.* **43**, 5034–5043 (2004).
16. Ohgo, Y. *et al.* Molecular structures of five-coordinated halide ligated iron(III) porphyrin, porphycene, and corrphycene complexes. *Inorg. Chem.* **41**, 4627–4629 (2002).
17. Strauss, S. H. *et al.* Comparison of the molecular and electronic structures of (2,3,7,8,12,13,17,18-octaethylporphyrinato)iron(ii) and (*trans*-7,8-dihydro-2,3,7,8,12,13,17,18-octaethylporphyrinato)iron(ii). *J. Am. Chem. Soc.* **107**, 4207–4215 (1985).
18. Schünemann, V. *et al.* The 5/2,3/2 spin admixture in the chloroiron(III) derivative of the sterically crowded 2,3,7,8,12,13,17,18-octaethyl-5,10,15,20-tetraphenylporphyrin. *Angew. Chem. Int. Ed.* **38**, 3181–3183 (1999).
19. Nemykin, V. N. & Hadt, R. G. Influence of Hartree−Fock Exchange on the Calculated Mössbauer Isomer Shifts and Quadrupole Splittings in Ferrocene Derivatives Using Density Functional Theory. *Inorg. Chem.* **45**, 8297–8307 (2006).
20. Ercolani, C., Gardini, M., Murray, K. S., Pennesi, G. & Rossi, G. Crystalline isomerism in (μ-Oxo)bis[(phthalocyaninato)iron(III)]: further characterization of the isomer having a linear or quasi-linear Fe–O–Fe bond system (μ-Oxo(2)). *Inorg. Chem.* **25**, 3972–3976 (1986).
